# Logistic Stewardship: Supporting Antimicrobial Stewardship Programs Based on Antibiotics Goods Flow

**DOI:** 10.3390/antibiotics14010043

**Published:** 2025-01-06

**Authors:** Bianca Leistner, Dominic Rauschning, Ralf Matthias Hagen, Franziska Srečec, Nico Tom Mutters, Ruth Weppler, Christina Mutschnik, Manuel Döhla

**Affiliations:** 1 Institute for Hygiene and Public Health, Medical Faculty, University of Bonn, Haus 33, 53113 Bonn, Germany; biancaleistner@bundeswehr.org (B.L.);; 2 Department XVI of Laboratory Medicine, Bundeswehr Central Hospital Koblenz, 56070 Koblenz, Germany; 3 Clinic Ia of Internal Medicine, Bundeswehr Central Hospital Koblenz, 56070 Koblenz, Germany; 4 Department XXI of Microbiology and Hospital Hygiene, Bundeswehr Central Hospital Koblenz, 56070 Koblenz, Germany; 5 Department XXIV of Hospital Pharmacy, Bundeswehr Central Hospital Koblenz, 56070 Koblenz, Germany

**Keywords:** antimicrobial stewardship, quality indicator, defined daily dose, prescribed daily dose, antibiotic resistance, antibiotic consumption, infection control, clinical decision making, surveillance

## Abstract

Background/Objectives: Antimicrobial resistance is a global threat to safe health care, and a reduction in antibiotic consumption seems to be an appropriate preventive measure. In Germany, the reporting of hospital antibiotics consumption to an independent institution is only voluntary. Although a high level of willingness to improve can be assumed in the case of participation, the median consumptions of reporting hospitals change only slightly. This study examines the question of whether the logistical consumption figures adequately reflect real consumption, and if not, how to optimize the use of logistical data for clinical decisions. Methods: Four selected wards were analyzed during six months. A retrospective analysis of patient case files was performed to receive “prescribed daily doses” (PDDs). These were compared to “defined daily doses” (DDDs) from logistical data. Additional inventories were performed to calculated stored antibiotics. Antibiotics goods flows were presented via waterfall diagrams to identify logistic patterns that could explain PDD/DDD quotients. Antimicrobial stewardship (AMS) quality indicators were analyzed to give advice for optimized clinical AMS measures. Results: The total PDD/DDD quotient was 0.69. Four logistical patterns were identified. Optimized prophylaxis, AMS consultations and reevaluation of therapy seem to be the most useful measures to reduce PDDs. Conclusions: If AMS programs rely solely on DDDs, measures cannot be optimal. A complete consideration of antibiotic goods flows supports clinical decisions, but is very costly in terms of data collection. The consideration of logistical data can help to identify areas of focus for AMS programs. Therefore, specialists of antibiotics logistics should complement clinical AMS teams.

## 1. Introduction

Antimicrobial resistance (AMR) is one of the biggest global healthcare challenges of the 21st century. Evaluations by the Antimicrobial Resistance Collaborators revealed an estimated 8.9 million deaths worldwide due to infectious diseases in 2019, and 4.95 million of these deaths were associated with AMR. It is assumed that AMR was the cause of death in 1.27 million of these cases [1]. An increase of 1.19 million deaths per year due to AMR and an additional 8.22 million deaths per year associated with AMR could be expected by 2050 [2]. 

At the beginning of the 21st century, politicians recognized that the development and spread of AMR must be stopped. To this end, member states of the United Nations Foundation made commitments at an international level to a coordinated approach, which was reflected in the establishment of antimicrobial stewardship (AMS) in human medicine. Since then, action plans have been drawn up at a national level, which have been further developed in line with the One Health approach and repeatedly underline the topicality of the importance of the rational use of antibiotics [3,4,5,6].

Rational use of antibiotics does not necessarily mean that antibiotic prescriptions must also be reduced. However, quantitatively high antibiotic consumption has already been identified as a stand-alone risk factor for the development of AMR [7,8].

As early as the 1960s, the World Health Organization (WHO) recognized the need for the use of drugs to be comparable between different countries. To this end, a working group drew up guidelines on relevant data and their collection modalities [9]. The WHO has defined a standard for recording drug consumption using the “ATC (anatomical therapeutic chemical)/DDD (defined daily dose)” classification in order to ensure standardization and thus comparability of data. The classification uses the number of daily doses of an active substance standardized to community or hospital activity data (e.g., DDD per 1000 inhabitants or DDD per 100 patient days) as a parameter [10].

In the European Union, the ESCA-Net of the European Centre for Disease Prevention and Control (ECDC) can be used to compare the collected national data for the consumption of antibiotics [11]. Data from Germany were included here for the year 2022 as community consumption only; no data for the hospital sector were reported. This is because there is no mandatory reporting of antibiotic consumption data from hospitals to a central body in Germany. 

In Germany, the Infection Protection Act § 23 paragraph 4 [12] obliges hospitals and outpatient surgery facilities to manage their antibiotic consumption. To meet this objective, the consumption of antibiotics is to be recorded and evaluated, conclusions are to be drawn and the necessary adjustments to antibiotic use are to be implemented. The Robert Koch Institute (RKI) specifies the form in which the data should be prepared and presented; the use of the pharmacies’ delivery data and of the WHO ATC-DDD-classification are recommended [13].

**Figure 1:**
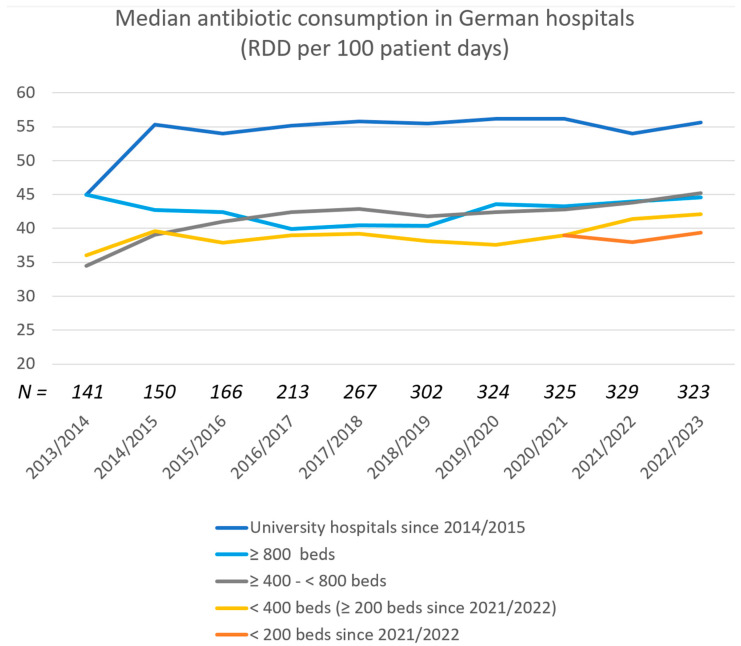
Use of antibiotics in German hospitals according to the ADKA-if-DGI-Project [16,20,21,22,23,24,25,26,27,28] X-scale: year (second half of year one, first half of year two). N: number of reporting hospitals. Y-scale: median RDD/100 patient days for different hospital sizes.

However, there is no legal requirement to report consumption data to an independent institution. Voluntary reporting of these data to a surveillance system is possible; there are the RKI’s Antibiotic Consumption Surveillance [14] and the German Society for Infectious Diseases’ Anti-infective Surveillance Project (ADKA-if-DGI) [15] that both allow benchmarking of reported data with other participants. The ADKA-if-DGI project, which has existed since 2007, publishes a report annually in the third quarter that covers the second quarter of the year before last and the first quarter of the previous year (the 2022/2023 report was published in September 2024) [16]. Median consumption of antibiotics of the participating hospitals is shown in Figure 1. 

The German S3 guideline for the rational use of antibiotics in hospitals has existed since 2013 [17] and was revised in 2019 [18]. Zeidler et al. [19] were able to show that implementing this guideline can positively influence a hospital’s antibiotic consumption. It is therefore surprising that the consumption of the hospitals participating in the ADKA-if-DGI project have remained almost unchanged and show no downward trend (Figure 1). Assuming that the voluntary participation of hospitals in a benchmark is mainly perceived by hospitals that rate themselves as good and promote an active AMS, a downward trend in consumption should be recognizable over a longer period of time.

This paper therefore aims to answer the question of whether antibiotic consumption, which is calculated using logistics data, differs from actual antibiotic consumption. The hypothesis is that the course of antibiotic consumption seen, for example, in ADKA-if-DGI does not correspond to the actual consumption of antibiotics. If this is the case, this study should investigate whether reasons for these deviations can be identified and whether, based on this knowledge, better AMS measures could be derived than on the basis of consumption data.

## 2. Methods

The Bundeswehr Central Hospital Koblenz is categorized as a 400–800 bed hospital according to ADKA-if-DGI. In 2012, an AMS commission was set up, and in 2021 an interdisciplinary AMS team was formed, consisting of clinical infectious disease specialists, microbiologists, clinical pharmacists and hygienists [19]. 

In the June 2022 ADKA-if-DGI report for 2020/2021, the hospital was ranked first in its benchmark group, despite extensive AMS measures [19]. Therefore, four wards in the hospital were selected to answer the research question, where the movement of antibiotics was to be examined more closely in parallel with the consumption data over a study period of 6 months (July 2022 to December 2022). These wards were

1. a conservative peripheral ward (CPW);
2. a conservative intensive care ward (CICW);
3. a surgical intensive care ward (SICW);
4. a surgical peripheral ward (SPW).

The selection was based on previous antibiotic consumption data analyses in which the selected wards showed a high consumption of antibiotics. Furthermore, these four wards were the only wards in the hospital that were exempt from the restricted use of reserve antibiotics.

### 2.1. Recording of Documented Consumption

The total number of cases treated, and the proportion of cases treated with antibiotics on the selected wards were identified on the basis of a review of all case files of the affected wards using the hospital information system (NEXUS HIS Release 8 SR24-2 Rev 9.19.06, NEXUS Deutschland GmbH). The paper files of the identified cases were then requested from the hospital archive. A manual study of the available files was carried out. In addition to patient charts, documents on interventions, operations and premedication protocols were also examined. For the intensive care wards, additional digital patient charts, kept in the data management system ICCA J.01.00 (Philips Medizin Systeme, Böblingen, Germany), were also examined. All extracted data were stored and processed anonymously. 

For each antibiotic administration, the name and total dose in milligram of the antibiotic were obtained. At the peripheral wards, documented indications and further AMS quality indicators were recorded. These data were not recorded from the intensive care units because a massive bias towards false-positive findings was expected due to the weekly AMS visits to all patients in the intensive care units [19]. In regard of logistic aspects, the following AMS quality indicators were defined with regard to the German S3 guideline for the rational use of antibiotics in hospitals [18]:

5. compliance with the in-house antibiotic guideline for prophylaxis;
6. compliance with the in-house antibiotic guideline for calculated therapy;
7. request for an AMS consultation;
8. therapy reevaluation after 72 h;
9. performance of microbiological diagnostics;
10. indication of a therapy limitation;
11. use of therapeutic drug monitoring (TDM).

### 2.2. Recording of Logistical Data

The documented receipts of each antibiotic were determined on the basis of deliveries, return deliveries and prescription deliveries via the output from the warehouse management system (SASPF (SAP SE), Walldorf, Germany) for each selected ward per calendar month of the study period. All quantities were converted into milligrams.

### 2.3. Inventories on the Wards

During the study period, inventories were carried out at all selected wards on the 1st of each month from July to December and on the 31st of December. All antibiotics with ATC codes J01, J02 and J04 were recorded in different dosage forms with the corresponding batch numbers and expiry dates according to the smallest tangible units (e.g., tablet, ampoule, capsule, etc.). The quantity of active ingredient units was determined in milligrams from LAUER-TAXE^®^ Online 4.0 (CGM Lauer, Koblenz, Germany), a database for all drugs approved in Germany, and then multiplied by the number of units counted per month.

### 2.4. Evaluation and Presentation

The statistical software Stata IC 15.1 (Stata Corp., Texas, TX, USA) was used for the analysis and graphical presentation.

#### 2.4.1. PDD/DDD Quotients

The doses of antibiotics prescribed on record were converted from milligrams to PDDs for each antibiotic using the German version of the WHO ATC-DDD index for 2022 [29]. This was done individually for each antibiotic in each dosage form for each selected ward for each month of the study period. In the same way, antibiotics consumption based on the logistic data was converted from milligram to DDDs. PDDs and DDDs are mathematically the same unit, but PDD implies documented prescription. The individual antibiotics were summarized into 13 antibiotic groups, which correspond to the antibiotic groups of the ADKA-if-DGI project [16] (Table 1). They will be further referred to as “ADKA groups”.

PDD/DDD quotients were calculated for the four selected wards, as well as total PDD/DDD quotient. A PDD/DDD quotient below 1.00 indicated an over-estimation of antibiotics consumption based on DDDs, and a PDD/DDD above 1.00 indicated an under-estimation of antibiotics consumption based on DDDs.

The distribution of PDDs and DDDs over the 13 ADKA groups was calculated and tested for differences using a nonparametric testing (Chi^2^ test, alpha = 0.05). 

#### 2.4.2. Logistical Patterns

The doses of stored antibiotics seen during the inventories were converted from milligrams to DDDs for each antibiotic using the German version of the WHO ATC-DDD index [29]. This was done individually for each antibiotic in each dosage form for each selected ward for each month of the study period. 

Waterfall diagrams were created for each ADKA group in the selected wards. They were coded using the inventories (dark grey bar starting at 0 and going upwards, axis label “*month*_inventory”), logistical data (green bar starting at the maximum value of the inventory bar and going upwards, axis label “*month*_logistics”) and documented consumption data (green bar starting at the maximum value of the logistical data and going downwards, axis label “*month*_consumption”) for each month. Virtual inventories were then calculated as

Virtual inventory_(next month)_ = Inventory_(month)_ + logistical data_(month)_ − documented consumption_(month)_ (1)

Virtual inventories were represented as light grey bars starting at 0 and going upwards (axis label “*month*_virtual”). The difference between the virtual inventory of a month and the real inventory of the following month was represented as a red bar that started on the maximum value of the virtual inventory and ended on the maximum value of the real inventory of the following month (axis label “*month*_delta”); it could go upwards or downwards, indicating more existing inventory or less inventory as calculated, respectively. 

#### 2.4.3. AMS Key Findings

The distributions of documented indications at the peripheral wards were calculated based on hospital stays (HSs) and on PDDs. Chi^2^ testing was used to calculate significant differences between HSs and PDDs per ward, as well as differences between HSs on CPW and SPW (alpha = 0.05); PDDs were rounded for this purpose. The proportions of correct HSs and PDDs were calculated for each AMS quality indicator per ward.

## 3. Results

### 3.1. PDD/DDD Quotients

Within the study period, 2289 HSs at the selected wards were identified, which could be assigned to 2105 cases. The difference between HSs and cases is explainable due to transfers between wards. Antibiotic administration was documented in 1115 HSs. A total of 5763.04 PDDs and 8377.19 DDDs were calculated. Table 2 gives further details.

HSs at conservative wards led less frequently to the administration of antibiotics than stays at surgical wards (about one-third and two-thirds, respectively), while the difference between peripheral ward and intensive care unit within a specialty was less pronounced. However, when antibiotics are administered, more antibiotics per hospital stay were administered at the conservative wards and more PDDs were generated. Comparing the PDDs with the DDDs, the DDDs overestimated actual consumption overall and at CPW, CICW and SPW, while they underestimated the actual consumption at SICW. 

Table 3 shows the PDD/DDD quotients of all 13 ADKA groups per selected ward and in total. Four PDD/DDD quotients could not be calculated due to a DDD of 0.0. The median of the other 48 PDD/DDD quotients is 0.69 [IQR: 0.51–0.98; Min: 0.00; Max: 8.06] and identical to the calculated total PDD/DDD quotient from Table 2.

Table 3 also shows the absolute PDDs and DDDs for the 13 ADKA groups across the selected stations and in total as well as the relative PDDs and DDDs per ward. All PDD distributions differed significantly from the DDD distributions. CPW most often consumed broad-spectrum penicillin (ADKA group 2, 20.99%) according to PDDs, while folate antagonists (ADKA group 12, 23.40%) generated the greatest consumption according to DDDs. CICW most often consumed carbapenems (ADKA group 3, 24.46%) according to PDDs, while macrolides and clindamycin (ADKA group 10, 23.40%) generated the greatest consumption according to DDDs. SICW most often consumed carbapenems (ADKA group 3) according to PDDs as well as DDDs (27.98% and 24.79%, respectively). SPW most often consumed 1st and 2nd generation cephalosporins (ADKA group 4) according to PDDs as well as to DDDs (28.58% and 27.89%, respectively). Overall, the selected wards most often consume broad-spectrum penicillin (ADKA group 2) according to PDDs as well as to DDDs (21.97% and 15.76%, respectively).

### 3.2. Logistical Patterns

A total of 52 waterfall diagrams, one for each ADKA group per selected ward, were generated (Figures S1–S52). Regardless of the specific pathways of the waterfall, it can be seen that undocumented inflows and outflows of antibiotics essentially take place continuously.

Four notable logistical patterns can be identified here:

12. “Payback”, where selectively documented inflow in one month is followed by an undocumented outflow in another month or vice versa (Figures S5, S6, S10–S12, S19, S21, S23, S28, S29, S40 and S45);
13. “Cross-supply”, where regularly documented inflow is followed by a smaller documented outflow and higher virtual than real inventory in the following month (Figures S2, S3, S5, S15, S16, S18, S19, S21, S23, S41, S43, S44 and S52);
14. “Blackbox”, where undocumented inflow and outflow occur over time, parallel to regular documented inflow and outflow (Figures S4, S7, S9, S16, S19, S20, S24, S26, S32 and S34);
15. “Hoarding”, where the inventory of each month is far higher of the outflow, while still seeing documented inflow (Figures S17–S19, S20, S23, S35, S36, S40, S46, S49 and S52).

### 3.3. AMS Key Findings

#### 3.3.1. Indications

Table 4 shows the distribution of HSs with antibiotic administration and PDDs at the two peripheral wards. For both wards, the relative frequencies of the indications differed significantly between HSs and the generated PDDs (CPW: Chi^2^ (7) = 84.2839, *p* = 0.000; SPW: Chi^2^ (7) = 440.9425, *p* = 0.000). At both wards, most PDDs were generated by the calculated therapy (46.09% and 64.05% respectively); at CPW, this corresponded with the highest relative HSs (46.97%), while at SPW, the highest relative HSs were generated by prophylaxis (57.56%).

At CPW, about 15% of all HSs generated about one-third of all PDDs on a microbiological basis (targeted therapy, microbiological de-escalation and microbiological escalation), while at SPW about 5% of all HSs generated less than 10% of PDDs. For prophylaxis, relative HSs and generated PDDs were only about half as frequent at CPW as at SPW (HSs: 24.21% and 57.56%, respectively; PDD: 8.77% and 16.84%, respectively). At both wards, about 8% of the relative HSs and generated PDDs was based on clinical decisions, with escalations more common at the conservative ward and de-escalations more common at the surgical ward.

#### 3.3.2. AMS Quality Indicators

Detailed information for the statistical tests between HSs and PDDs for each quality indicator for both peripheral wards is given in Table 5.

The antibiotic prophylaxis for HSs at both wards corresponded to a high degree with the in-house guideline (94.7% and 90.2%, respectively) without statistically significant differences (Chi^2^ (1) = 1.5743, *p* = 0.210). However, significantly fewer PDDs were administered correctly (72.8% and 85.1%, respectively).

Calculated initial treatment was significantly more often performed correctly at CPW than at SPW (82.7% and 71.5%, respectively, Chi ^2^ (1) = 6.5065, *p* = 0.011), the proportion of generated PDDs of correct treatment did not differ significantly at both two wards.

Significantly fewer AMS consultations were performed at CPW than at SPW (1.7% and 11.3%, respectively; Chi^2^ (1) = 23.1830, *p* = 0.000); the PDDs generated by AMS consultations were significantly higher at CPW (20.8%) and, even if not significant, also higher at SPW (2.8%). 

A re-evaluation was documented significantly less often at CPW than at SPW (23.0% and 45.7%, respectively; Chi^2^ (1) = 32.5025, *p* = 0.000); in both cases, significantly more correct PDDs (31.1% and 55.1%, respectively) were generated. 

Microbiological diagnostic was performed significantly more often at CPW than at SPW (47.0% and 34.8%, respectively; Chi^2^ (1) = 8.8376, *p* = 0.003). The generated PDDs did not differ significantly from the proportion of HSs on both wards (52.9% and 38.3%, respectively).

A defined limit of therapy was set with equal frequency on both wards (31.2% and 25.4%, respectively; Chi^2^ (1) = 2.4370; *p* = 0.119). The PDDs generated from the correct therapy limits were not significantly different at both wards compared to the proportion of hospitalizations (26.9% and 21.1%, respectively).

TDM was used significantly more often at CPW than at SPW (39.9% and 7.1%, respectively; Chi^2^ (1) = 54.0087, *p* = 0.000). The proportion of HSs with TDM and the proportion of PDDs generated (48.0% and 8.7%, respectively) did not differ on the two wards.

## 4. Discussion

### 4.1. PDD/DDD Quotient at SICW

When comparing the documented consumption of antibiotics to the logistic data, an overall PDD/DDD quotient of 69% was calculated. This indicates an overestimation of antibiotic consumption when using DDDs, which has already been described several times [30,31]. The difference is usually explained in the studies carried out for this purpose by standardizing the DDDs to a 70 kg adult without impaired organ function [30].

The overestimation has so far been described in the context of point prevalence studies. This study, on the other hand, covered a period of six months at four selected wards and makes it possible to identify logistical patterns that can provide further explanations for the overrepresentation of DDDs and thus derivations for further AMS measures. 

**Figure 2:**
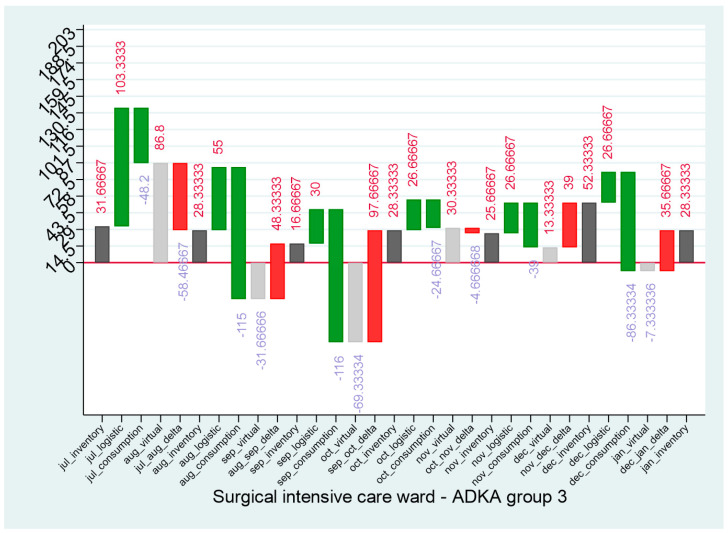
Waterfall diagram of SICW—ADKA 3.

For example, it was shown that the DDDs of the SICW, against the trend, underestimated the documented consumption by 42%. When the distribution of this consumption is considered in terms of the ADKA groups (Table 3), a quantitative excess of consumption can be seen, but no qualitative indication of the cause of this underestimation can be derived. Here, a look at the waterfall charts (Figures S27–S39) can be helpful, for example for carbapenems (ADKA group 3, Figure 2). It turns out that the virtual stocks in September, October and at the end of December are negative, which means that more carbapenem was consumed at the ward than was physically present. This is accompanied by large deltas with an inexplainable increase in antibiotics, which can also be seen for other antibiotics (especially in ADKA groups 2 (Figure S28), 4 (Figure S30), 5 (Figure S31), 6 (Figure S32)).

The waterfall diagrams therefore show logistical irregularities that cannot be explained by the data themselves. Further research revealed in the case of this particular SICW a close cooperation with an associated intermediate care ward. Both wards have individual cost centers, but medication is often ordered jointly from the pharmacy. Therefore, it can be assumed that orders were primarily placed via the cost center of the intermediate care ward. These assumptions are supported on the one hand by the very low order quantities of the intermediate care ward according to the warehouse management system, and on the other hand by general statements of the personnel of both wards on their ordering behavior.

So, these orders are not reflected in the DDD consumption data of the SICW. Assuming that the antibiotics for both wards were provided exclusively via the SICW cost center, and that both wards have similarly high real consumption, the corrected PDD/DDD quotient would be 1.42/2 = 0.71, which corresponds to the PDD/DDD quotient of 0.69 for the entire hospital. 

For future surveillance and for deriving AMS measures such as training or consumption restrictions, this peculiarity requires attention, and the SIWC must be considered together with the intermediate care station. Alternatively, a better separation of the data from both stations can be aimed for in the future by increasing cost center discipline.

### 4.2. PDD/DDD Quotients at CPW, CICW and SPW

Apart from this special case, the data from this study show an overestimation of antibiotic consumption when using DDDs. In the following, possible factors that lead to this overestimation of real consumption will be discussed on the basis of the logistics chain from ordering to taking the antibiotic. 

In addition, measures in the sense of logistic stewardship will be described that could be used as part of an AMS program to control or eliminate these factors.

#### 4.2.1. Ordered Is Not Delivered

A delivery bill or other documentation of pharmacy dispensing is not the same as the actual receipt of goods on the ward. Deliveries may reach the wrong addressee or get lost in transit. However, as this would be noticed by the ordering ward in the case of critical deliveries, which include antibiotics, this error factor can be virtually ruled out.

As a logistic stewardship measure, a well-functioning complaints management system is useful for correcting any incorrect deliveries.

#### 4.2.2. Delivered Is Not Stocked

On the basis of the logistical patterns with a lot of undocumented inflow and outflow of antibiotics on all selected wards for most of the ADKA groups, it can be deduced that delivered antibiotics are lent and returned immediately or over time between wards. The “payback” logistical pattern shows that although anti-infectives are delivered to wards, they are not necessarily administered there, but are also lent out and returned between wards. This cross-supply may be possible in difficult supply situations due to supply bottlenecks. During the period under review, there were supply bottlenecks for folate antagonists, which are frequently used for prophylaxis by the CPW [32,33,34]. At this ward, this logistical pattern was evident for this class of antibiotics in the sense of the lending party. In addition, the “payback” pattern was also observed for other ADKA groups. It is assumed that “artificial” bottlenecks occurred on the ward for organizational reasons, with insufficient order quantities in relation to actual demand. In turn, the wards that increasingly dispense antibiotics from their stock to other wards exhibit the logistical pattern of “cross-supply” in certain antibiotic classes, where undocumented consumption was regularly high. One example is macrolides in the CICW. The lending stations could also be found in the “black box” pattern, where unusually high undocumented accesses could also be observed. Prior to the review, it was assumed that there was primarily a cross-supply of the reserve antibiotics defined in the hospital, which may only be regularly ordered by the wards defined by the AMS commission, and an order by another ward is only accepted after an AMS consultation and corresponding justification by a specialist on the ward. However, all the wards in question were authorized to order reserve antibiotics directly from the pharmacy. This meant that they could order any reserve antibiotics at all times. It was therefore not possible to examine other stations not authorized to do so and whether this logistical pattern can be found again.

From the logistic stewardship perspective, it is important to maintain strict cost center discipline and good logistics planning. Although cross-supplies may be necessary in exceptional cases, they should not become the norm. A bonus–malus system for cost center managers, for example in the form of bonus payments or special leave for good cost center management, could be an appropriate measure here.

#### 4.2.3. Stocked Is Not Used 

For some antibiotics, a logistical pattern of “hoarding” can be seen (as in Figures S10 and S40) There is a clear overstocking behavior, with ranges over half a year despite low consumption. This type of storage behavior entails the risk that the anti-infectives are not available to other areas of the hospital. There is no conclusive justification for this request behavior, since according to Section 30 of the Pharmacy Operating Regulations [35], the hospital pharmacy is obliged to keep the average requirement of medicines and medical products in stock for two weeks. In addition, the wards in the hospital in question are supplied at least once a week in line with demand and, in accordance with Section 33 of the Pharmacy Operating Regulations, a standby service also ensures that demand is met at short notice outside of opening hours. In the evaluation of antibiotic consumption, these ward stocks are critical, as an increased number of expired antibiotics, which are often not returned to the pharmacy, are counted as consumption for the cost center and thus significantly increase the DDDs. Although the regulations for returning expired medicines for safe disposal or medicines that are no longer required for re-use on other wards are regulated at the hospital in question, this option is rarely used. In general, expired antibiotics are often disposed of via solid waste and wastewater [36,37,38,39,40]. While hospital waste is usually incinerated, antibiotics in wastewater can accumulate and lead to the development of resistance in the environmental microbiome [41,42,43,44,45].

In this regard, logistic stewardship could re-examine and adapt the modalities of the return management system, for example via training, including creating an understanding of this measure. Another measure could be the introduction of an active return management system, in which antibiotics that are no longer needed are explicitly returned by the wards to the pharmacy. This can save costs and avoid drug waste.

#### 4.2.4. Used Is Not Prescribed

The dispensing of discharge medication by a hospital pharmacy is permitted in accordance with Section 14 (7) of the Pharmacy Act 24 [46] in order to ensure treatment until the next possible outpatient contact with a doctor. However, in Germany military patients are subject to free medical care by military doctors; it cannot be ruled out that these patients are given more generous quantities of oral medication, especially if antibiotic therapy has already been started in hospital and the period of administration has not yet elapsed. These outflows to the outpatient sector are not documented separately and are reflected as apparent inpatient consumption. These discharges are not documented in the patient files and could only be estimated; they should be object for further research for a better quantification of this goods flow.

Logistic stewardship should therefore enforce a discharge management and the comprehensive training of medical and nursing staff in dealing with the discharge measure. This is not only sensible from an AMS point of view, but also from the point of view of drug therapy safety, which is why the introduction of discharge management should be specifically aimed for.

#### 4.2.5. Prescribed Is Not Administered

After a prescription has been issued, various reasons may speak against an administration. For example, an antibiotic may be prepared but then discarded because new information is available (antibiogram, blood count) and the medication is therefore adjusted. It is also possible that a preparation is discarded if an error occurs during the preparation process (unhygienic working, uncertainty regarding the correct dosage). It is impossible to estimate the proportion of these antibiotics that are not administered. To achieve a reliable quantification, it would be necessary to observe the process over a longer period of time and across several wards or hospitals.

Logistic stewardship could ensure that changes are documented in the patient file to make these “consumptions” traceable and thus assessable for further AMS measures.

#### 4.2.6. Administered Is Not Documented

Since the data for the peripheral wards were obtained from paper files, the documentation quality in a paper file represents a potential source of error for the traceability of the medication process. Not only the legibility of the entries, but also the entry as such influence the complete recording of the PDDs. Furthermore, documentation on perioperative prophylaxis, for example, could be found in the progress curve, in the anesthesia protocol or in the surgical protocol in this project. In addition to under-documentation, multiple documentation of the same antibiotic could even be found in several places; this could lead to an overestimation of the PDDs, at least for certain antibiotics, such as 1st and 2nd generation cephalosporins (ADKA group 4). In fact, a higher proportion of ADKA group 4 was seen in PDDs compared to DDDs on CPW, SICW and SPW. However, since the evaluation of the paper files was carried out by one person in each case, multiple counts of the same antibiotic can be largely excluded, so this is unlikely to explain this phenomenon.

But, to counter these problems via logistic stewardship, the introduction of digital patient files is mandatory. Until then, training in correct and legible documentation can improve data quality. 

#### 4.2.7. Documented Is Not Taken

This factor is particularly important because the PDDs ultimately represent the documented doses. However, the PDDs will also overestimate the actual consumption in terms of tablets actually taken. There are various reasons why a patient may not take their medication despite a prescription, administration and documentation. This may be intentional or unintentional. 

Logistic stewardship cannot contribute to this case. From a general AMS point of view, clinical-pharmaceutical support measures (“pharmacists on the ward”) can be considered for this discrepancy between documentation and actual intake. Psychiatric and demented patients must receive optimal medication. Potential interactions of the basic medication with the antibiotics given should be considered. Therapeutic drug monitoring can also make a valuable contribution here.

### 4.3. AMS Key Findings

#### 4.3.1. Comparison of Both Wards

The proportion of guideline-compliant prophylaxis was roughly the same in percentage terms at the two peripheral wards. However, a direct comparison seems difficult, as these tend to be used as immune-prophylaxis on the CPW and as perioperative antibiotic prophylaxis on the SPW. The guideline compliance for calculated initial therapies, the request behavior for microbiological diagnostics, AMS consultations and TDM on the CPW are significantly better. On the other hand, the re-evaluation of the calculated antibiotic therapy after 72 h is carried out significantly more frequently on the SPW. A series of point prevalence analyses could be used to check whether this is a specialty-specific problem (conservative vs. surgical) and, if necessary, to work out in which clinical diagnoses deviations occur more frequently. Training measures must be carried out accordingly. 

In addition, a differentiated evaluation of whether there is a deviation from the house guideline due to ignorance or due to patient-specific reasons would be necessary. For this purpose, a justified deviation would have to be documented or, in the case of a digital patient file, queried in order to be able to collect the corresponding data. 

Since this study is based on the file documentation, with no integration of the microbiological findings into the hospital information system, an under-collection can be assumed here, but it can nevertheless be assumed that microbiological diagnostics as a tool in AMS is significantly underestimated [47]. Awareness of the specific requirement for microbiological diagnostics for the optimization of antibiotic therapy is to be increased through training measures. The integration of microbiological findings in the hospital information system was established as a result of this study, so a significantly higher documentation rate can be expected in the future. This could be verified using point prevalence rates. 

The higher proportion of TDM and AMS consultation on the CPW can be explained by the patient population, which is likely to be more multimorbid on the CPW, and the frequent use of prolonged to continuous intravenous administration. The benefits of prolonged and continuous administration of antibiotics, especially in critically ill patients, have already been demonstrated in the literature [48,49,50].

The initial limitation of therapy duration is a very easy-to-record and efficient AMS tool; nevertheless, it was only used rarely on both peripheral wards, despite the additional use of an implemented memory system. The primary purpose of this documentation is to ensure that antibiotics are not administered unnecessarily beyond the necessary duration of treatment [51]. Further training measures for both medical and nursing staff would appear to make sense here. Success is to be checked over time by means of point prevalence analyses and, if compliance falls, training measures are to be repeated.

The availability of data plays a relevant role in verifying compliance with AMS measures. Various media were used to collect data in this study. In addition, different types of documentation were found, which made manual data analysis more difficult and prone to errors. In order to achieve a consistently good data situation, the introduction of a comprehensive digital patient record would be of great benefit. In addition to automated data collection, this software can also be used to store a set of rules for the use of antibiotics in order to directly and indirectly support compliance with AMS measures.

#### 4.3.2. Comparison of HSs and PDD

An analysis of the fulfillment rates in relation to the HSs and PDDs shows that there is significantly less compliance with the guidelines in relation to the PDDs. On the CPW, approximately 5% of incorrect prophylaxis in the cases lead to approximately 28% of incorrect PDDs. For the SPW, the discrepancy of 10% in the cases and 15% in the PDDs is not as great, but is also significant. Studies analyzing AMS quality indicators conclude that the guidelines for calculated therapy are important [47,52,53], but from a logistic point of view the focus on correct prophylaxis seems to be a more important measure.

AMS consultations led to significantly more adequate PDDs compared to the proportion of cases. One possible explanation is that a consultation is primarily requested for complex cases [54] that require longer treatment durations and higher dosages, so that the correct PDDs accumulate from an initial consultation decision. AMS consultations as a special form of an infectious diseases specialist consultation may also optimize the generated PDDs in the long term [55].

Periodical re-evaluations also led to significantly more correct PDDs compared to the proportion of cases. This can be explained by the fact that mandatory revalidation makes it possible to discontinue a therapy earlier and to switch to a targeted therapy with a narrower spectrum earlier. These logistic findings are in line with the clinical benefits of a shorter antibiotics therapy [56,57].

Other quality indicators showed no significant differences between the proportion of cases and PDDs generated. This finding does not indicate that other quality indicators do not have any benefit at all, as various studies show [53,54,55,58].

But, for a disproportionate PDD optimization in this study, improved guideline compliance of the prophylaxis, an increase in the proportion of AMS consultations and a strict re-evaluation after 72 h should be prioritized. This applies irrespective of the CPW/SPW specialization.

### 4.4. General Opportunities and Threats of Logistic Stewardship

#### 4.4.1. Training of the AMS Team Members

Logistic stewardship, similar to diagnostic stewardship [59,60,61], is a subset of AMS that is outside the classic focus of AMS. In Germany, for example, training as an AMS expert takes 200 h without any training on logistical issues [62]. AMS teams consist of physicians and pharmacists [18], so that within such a team the pharmacists are predestined for logistic stewardship. However, for pharmacists there is no targeted training for “logistics”, neither for antibiotics nor in general [63]. 

Most pharmacists working in clinical AMS teams have completed a specialization in “clinical pharmacy” which requires a 3-year practical activity and several theoretical seminars [63,64]. Basic knowledge about AMS is taught as part of a 12-h-long theory block on “microbiology/antimicrobial therapy”, but also without any training on logistical issues [64]. Due to the potential of logistic stewardship shown in this study, it is therefore useful to develop possibilities for the formal qualification of AMS team members in the field of logistics. These team members can then act as multipliers in order to carry out the various discussed trainings of staff in the wards.

#### 4.4.2. Technical and Legal Aspects

However, a full introduction of logistic stewardship is a challenge even with appropriately qualified and trained staff. Even within a medium-sized hospital, where central logistics is provided by a single pharmacy, the complete recording of all goods flows is only possible under high time expenditure and remains, as shown, incomplete. Further investigations would have to answer the question of how logistic stewardship programs should be designed in order to be as efficient as possible.

In addition, legal hurdles may also exist. If the consumptions collected are not evaluated cumulatively, as in the present study, but on an individual level, a corresponding consent and consent of patients may be required [65]; if too many patients refuse to do so, the database is correspondingly incomplete.

#### 4.4.3. Public and Global Logistic Stewardship

In the EU, 20.0 DDD antibiotics per 1000 inhabitants were consumed in 2023, of which 1.61 DDDs per 1000 inhabitants were consumed in hospitals [11]. Community consumption therefore comprises about 92% of the total antibiotic consumption. Sales data and/or reimbursement data are used as sources for the calculation of these consumptions, depending on the country [11]. Further data sources with specific advantages and disadvantages exist worldwide: import records, domestic manufacturers, public sector procurement, wholesalers, donations, prescriber records, dispensing records, insurance records and/or commercial data sources are used, depending on the country [66]. 

All these data sources have the fact that they generate DDDs, not PDDs in common. The points discussed in this study therefore apply to all these data sources. So, it can be assumed that logistic stewardship can also contribute to the improvement of AMS measures at the public and global health level. In “high-income countries”, the focus might thereby be on the avoidance of overuse and misuse as well as the prevention of waste and environmental inputs. In “low-income and middle-income countries”, the focus might be more on stabilization of supply chains, optimization of storage conditions and fair distribution to avoid lack of access [67]. Logistic stewardship can represent an important building block in a multi-disciplinary approach.

## 5. Conclusions

If AMS programs rely solely on DDD consumption data, derived measures may not be optimal. A complete consideration of antibiotics goods flows (DDDs, PDDs, inventories) can support clinical decisions, but is very laborious in terms of data collection, at least when using paper-based documentation. When considering AMS quality indicators, the integration of logistical data can improve the purely case-oriented consideration and identify areas of focus for AMS programs. Specialists in logistic stewardship can therefore effectively support clinical AMS teams, since clinical AMS specialists often do not have the knowledge or skills to collect and analyze logistical data appropriately.

## Figures and Tables

**Table 1:** ADKA groups and assigned antibiotics.

ADKA Group	Group Title	Antibiotics
1	Third and fourth generation cephalosporins	Cefotaxime, Ceftriaxone, Cefpodoxime, Ceftazidime, Ceftazidime with Tazobactam
2	Broad spectrum penicillin	Piperacillin, Piperacillin with Tazobactam
3	Carbapenems	Ertapenem, Imipenem, Meropenem
4	First and Second generation cephalosporins	Cefazolin, Cefalexin, Cefuroxime
5	Aminopenicillins with beta-lactamase inhibitors	Ampicillin with Sulbactam, Amoxicillin with Clavulanic acid
6	Narrow spectrum penicillin	Ampicillin, Amoxicillin, Flucloxacillin, Penicillin G/V
7	Fluoroquinolone	Ciprofloxacin, Levofloxacin, Moxifloxacin
8	Glycopeptides and daptomycin	Vancomycin, Dalbavancin, Daptomycin
9	Aminoglycosides	Gentamicin, Tobramycin
10	Macrolides and clindamycin	Azithromycin, Clarithromycin, Erythromycin, Roxithromycin, Clindamycin
11	Tetracyclines	Doxycycline, Tetracycline, Tigecycline
12	Folate antagonists	Cotrimoxazole
13	Others	Aztreonam, Colistin, Fidaxomicin, Fosfomycin, Linezolid, Metronidazole, Nitrofurantoin, Nitroxoline, Rifampicin

**Table 2:** Cases, hospital stays, administration of antibiotics and generated PDD/DDD per selected ward and in total. CPW: conservative peripheral ward. CICW: conservative intensive care ward. SICW: surgical intensive care ward. SPW: surgical peripheral ward. DDD: defined daily dose. PDD: prescribed daily dose.

Item	CPW	CICW	SICW	SPW	Total
Cases	506	462	291	846	2105
Hospital stays (Hospital stays per case)	527 (1.04)	509 (1.10)	329 (1.13)	924 (1.09)	2289 (1.09)
Hospital stays with antibiotics administration	180	124	231	580	1115
Number of administrated antibiotics (Antibiotics per administration)	347 (1.93)	263 (2.12)	382 (1.65)	820 (1.41)	1812 (1.63)
PDDs (PDD per administration)	1471.38 (8.17)	1100.30 (8.87)	1533.76 (6.64)	1657.60 (2.86)	5763.04 (5.17)
DDDs (DDD per administration)	2735.54 (15.20)	1703.09 (17.73)	1082.36 (4.69)	2856.20 (4.92)	8377.19 (7.51)
PDD/DDD quotient	0.54	0.65	1.42	0.58	0.69

**Table 3:** PDDs and DDDs for all 13 ADKA groups per selected ward and in total. Yellow: highest relative PDDs per selected ward. Grey: highest relative DDDs per selected ward. CPW: conservative peripheral ward. CICW: conservative intensive care ward. SICW: surgical intensive care ward. SPW: surgical peripheral ward. DDD: defined daily dose. PDD: prescribed daily dose. ADKA groups according to Table 1.

ADKA Group	CPW		CICW		SICW		SPW		Total	
	PDD	DDD	PDD	DDD	PDD	DDD	PDD	DDD	PDD	DDD
	(%)	(%)	(%)	(%)	(%)	(%)	(%)	(%)	(%)	(%)
	PDD/DDD		PDD/DDD		PDD/DDD		PDD/DDD		PDD/DDD	
1	90.5	110	50.42	30	13.5	30	170	242.5	324.42	412.5
	(6.15)	(4.02)	(4.58)	(1.76)	(0.88)	(2.77)	(10.26)	(8.49)	(5.63)	(4.92)
	0.82		1.68		0.45		0.70		0.79	
2	308.86	357.14	260.71	262.86	398	228.57	298.68	471.43	1266.25	1320
	(20.99)	(13.06)	(23.69)	(15.43)	(25.95)	(21.12)	(18.02)	(16.51)	(21.97)	(15.76)
	0.86		0.99		1.74		0.63		0.96	
3	182.83	270	269.17	360	429.2	268.33	72	93.33	953.2	991.66
	(12.43)	(9.87)	(24.46)	(21.14)	(27.98)	(24.79)	(4.34)	(3.27)	(16.54)	(11.84)
	0.68		0.75		1.60		0.77		0.96	
4	98.17	88.33	20.83	41	270.08	135	473.83	796.67	862.91	1061
	(6.67)	(3.23)	(1.89)	(2.41)	(17.61)	(12.47)	(28.58)	(27.89)	(14.97)	(12.67)
	1.11		0.51		2.00		0.59		0.81	
5	151.58	303.33	33.33	128.33	30.33	30	263	495.33	478.24	956.99
	(10.30)	(11.09)	(3.03)	(7.54)	(1.98)	(2.77)	(15.87)	(17.34)	(8.30)	(11.42)
	0.50		0.26		1.01		0.53		0.50	
6	173.53	288.6	187.03	201.37	173.44	195.13	17.75	38.27	551.75	723.37
	(11.79)	(10.55)	(17.00)	(11.82)	(11.31)	(18.03)	(1.07)	(1.34)	(9.57)	(8.63)
	0.60		0.93		0.89		0.46		0.76	
7	64.25	85	36.5	60	9.5	7.5	66.88	125	177.13	277.5
	(4.37)	(3.11)	(3.32)	(3.52)	(0.62)	(0.69)	(4.03)	(4.38)	(3.07)	(3.31)
	0.76		0.61		1.27		0.54		0.64	
8	55.04	62.14	70.04	57.14	22.86	17.5	11.13	12.5	159.07	149.28
	(3.74)	(2.27)	(6.37)	(3.36)	(1.49)	(1.62)	(0.67)	(0.44)	(2.76)	(1.78)
	0.89		1.23		1.31		0.89		1.07	
9	26.83	3.33	0	0	5.46	8.33	3.5	0	35.79	11.66
	(1.82)	(0.12)	(0.00)	(0.00)	(0.36)	(0.77)	(0.21)	(0.00)	(0.62)	(0.14)
	8.06		n.a.		0.66		n.a.		3.07	
10	151.67	284.67	88.65	412	68.33	46.67	21.47	98.67	330.12	842.01
	(10.31)	(10.41)	(8.06)	(24.19)	(4.46)	(4.31)	(1.30)	(3.45)	(5.73)	(10.05)
	0.53		0.22		1.46		0.22		0.39	
11	0	160	0	5	0	0	0	25	0	190
	(0.00)	(5.85)	(0.00)	(0.29)	(0.00)	(0.00)	(0.00)	(0.88)	(0.00)	(2.27)
	0.00		0.00		n.a.		0.00		0.00	
12	104	640	12.5	35	1.5	0	14.21	37.5	132.21	712.5
	(7.07)	(23.40)	(1.14)	(2.06)	(0.10)	(0.00)	(0.86)	(1.31)	(2.29)	(8.51)
	0.16		0.36		n.a.		0.38		0.19	
13	64.13	83	71.13	110.39	111.55	115.33	245.17	420	491.98	728.72
	(4.36)	(3.03)	(6.46)	(6.48)	(7.27)	(10.66)	(14.79)	(14.70)	(8.54)	(8.70)
	0.77		0.64		0.97		0.58		0.68	
Chi^2^(12) (p)	367.4062 (**0.00**)		200.0319 (**0.00**)		66.5261 (**0.00**)		54.1628 (**0.00**)		647.6227 (**0.00**)	

**Table 4:** Indications of administered antibiotics for both peripheral wards and generated PDDs. CPW: conservative peripheral ward. SPW: surgical peripheral ward. HSs: Hospital stays with antibiotic administration. PDD: prescribed daily dose.

Indication	CPW		SPW	
	HSs (%)	PDD (%)	HSs (%)	PDD (%)
Prophylaxis	84 (24.21)	129.00 (8.77)	472 (57.56)	279.10 (16.84)
Calculated therapy	163 (46.97)	678.11 (46.09)	246 (30.00)	1061.62 (64.05)
Clinical de-escalation	8 (2.31)	20.92 (1.42)	36 (4.39)	102.08 (6.16)
Clinical escalation	19 (5.48)	91.52 (6.22)	7 (0.85)	39.29 (2.37)
Target therapy	41 (11.82)	359.38 (24.43)	13 (1.59)	47.09 (2.84)
Microbiologically supported de-escalation	3 (0.86)	24.29 (1.65)	12 (1.46)	30.67 (1.85)
Microbiologically supported escalation	12 (3.46)	94.17 (6.40)	11 (0.34)	50.50 (3.05)
Unknown	17 (4.90)	73.99 (5.03)	28 (2.80)	47.26 (2.85)
Total	347 (100)	1471.38 (100)	820 (100)	1657.60 (100)

**Table 5:** Selected AMS quality indicators for the conservative and surgical peripheral ward (CPW and SPW). Given is the proportion of correct hospital stays (HSs) in comparison with the proportion of correct generated prescribed daily doses (PDD).

Quality Indicator	CPW		SPW	
	HSs (%)	PDD (%)	HSs (%)	PDD (%)
Prophylaxis following inhouse guideline	71/75 ^1^ (94.7)	93.92/129.00 (72.8)	421/467 ^2^ (90.2)	237.49/279.10 (85.1)
Chi^2^ (1) (p)	14.5742 (**0.000**)		4.5454 (**0.033**)	
Calculated therapy following inhouse guideline	129/156 ^3^ (82.7)	548.73/678.11 (80.9)	173/242 ^4^ (71.5)	743.11/1061.62 (70.0)
Chi^2^ (1) (p)	0.2464 (0.620)		0.2194 (0.640)	
AMS consultation, excluding prophylaxis	29/257 ^5^ (11.3)	279.54/1342.38 (20.8)	6/348 (1.7)	37.95/1378.50 (2.8)
Chi^2^ (1) (p)	12.6983 (**0.000**)		1.1908 (0.275)	
Reevaluation after 72h, excluding prophylaxis	59/257 ^6^ (23.0)	417.24/1342.83 (31.1)	152/333 ^7^ (45.7)	759.53/1378.50 (55.1)
Chi^2^ (1) (p)	6.7954 (**0.009**)		9.6567 (**0.002**)	
Microbiological diagnostics, excluding prophylaxis	118/251 ^8^ (47.0)	709.48/1342.38 (52.9)	116/333 ^9^ (34.8)	527.79/1378.50 (38.3)
Chi^2^ (1) (p)	2.8688 (0.090)		1.3635 (0.243)	
Defined limit of therapy, excluding prophylaxis	79/253 ^10^ (31.2)	360.43/1342.38 (26.9)	84/331 ^11^ (25.4)	290.49/1378.50 (21.1)
Chi^2^ (1) (p)	2.0656 (0.151)		2.9531 (0.086)	
Therapeutic drug monitoring, excluding prophylaxis	59/148 ^12^ (39.9)	409.13/852.94 ^13^ (48.0)	14/196 ^14^ (7.1)	78.10/895.54 ^13^ (8.7)
Chi^2^ (1) (p)	3.3104 (0.069)		3.8763 (**0.049**)	

Note: ^1^ 9 missing, ^2^ 5 missing, ^3^ 7 missing, ^4^ 4 missing, ^5^ 6 missing, ^6^ 17 missing, ^7^ 6 missing, ^8^ 17 missing, ^9^ 12 missing, ^10^ 15 missing, ^11^ 10 missing, ^12^ 16 missing. ^13^ Only for AB, that are suitable for TDM; ^14^ 152 missing.

## Data Availability

Dataset available on request from the authors.

## Supplementary Materials

The following supporting information can be downloaded at: https://www.mdpi.com/article/10.3390/antibiotics14010043/s1, Figure S1: Waterfall diagram of CPW—ADKA 1; Figure S2: Waterfall diagram of CPW—ADKA 2; Figure S3: Waterfall diagram of CPW—ADKA 3; Figure S4: Waterfall diagram of CPW—ADKA 4; Figure S5: Waterfall diagram of CPW—ADKA 5; Figure S6: Waterfall diagram of CPW—ADKA 6; Figure S7: Waterfall diagram of CPW—ADKA 7; Figure S8: Waterfall diagram of CPW—ADKA 8; Figure S9: Waterfall diagram of CPW—ADKA 9; Figure S10: Waterfall diagram of CPW—ADKA 10; Figure S11: Waterfall diagram of CPW—ADKA 11; Figure S12: Waterfall diagram of CPW—ADKA 12; Figure S13: Waterfall diagram of CPW—ADKA 13; Figure S14: Waterfall diagram of CICW—ADKA 1; Figure S15: Waterfall diagram of CICW—ADKA 2; Figure S16: Waterfall diagram of CICW—ADKA 3; Figure S17: Waterfall diagram of CICW—ADKA 4; Figure S18: Waterfall diagram of CICW—ADKA 5; Figure S19: Waterfall diagram of CICW—ADKA 6; Figure S20: Waterfall diagram of CICW—ADKA 7; Figure S21: Waterfall diagram of CICW—ADKA 8; Figure S22: Waterfall diagram of CICW—ADKA 9; Figure S23: Waterfall diagram of CICW—ADKA 10; Figure S24: Waterfall diagram of CICW—ADKA 11; Figure S25: Waterfall diagram of CICW—ADKA 12; Figure S26: Waterfall diagram of CICW—ADKA 13; Figure S27: Waterfall diagram of SICW—ADKA 1; Figure S28: Waterfall diagram of SICW—ADKA 2; Figure S29: Waterfall diagram of SICW—ADKA 3; Figure S30: Waterfall diagram of SICW—ADKA 4; Figure S31: Waterfall diagram of SICW—ADKA 5; Figure S32: Waterfall diagram of SICW—ADKA 6; Figure S33: Waterfall diagram of SICW—ADKA 7; Figure S34: Waterfall diagram of SICW—ADKA 8; Figure S35: Waterfall diagram of SICW—ADKA 9; Figure S36: Waterfall diagram of SICW—ADKA 10; Figure S37: Waterfall diagram of SICW—ADKA 11; Figure S38: Waterfall diagram of SICW—ADKA 12; Figure S39: Waterfall diagram of SICW—ADKA 13; Figure S40: Waterfall diagram of SPW—ADKA 1; Figure S41: Waterfall diagram of SPW—ADKA 2; Figure S42: Waterfall diagram of SPW—ADKA 3; Figure S43: Waterfall diagram of SPW—ADKA 4; Figure S44: Waterfall diagram of SPW—ADKA 5; Figure S45: Waterfall diagram of SPW—ADKA 6; Figure S46: Waterfall diagram of SPW—ADKA 7; Figure S47: Waterfall diagram of SPW—ADKA 8; Figure S48: Waterfall diagram of SPW—ADKA 9; Figure S49: Waterfall diagram of SPW—ADKA 10; Figure S50: Waterfall diagram of SPW—ADKA 11; Figure S51: Waterfall diagram of SPW—ADKA 12; Figure S52: Waterfall diagram of SPW—ADKA 13.
