# Supplementary figures and images for: Logistic Stewardship: Supporting Antimicrobial Stewardship Programs Based on Antibiotics Goods Flow
Source: Antibiotics (Basel). 2025 Jan 6;14(1):43. doi: 10.3390/antibiotics14010043 (PMC11761244; doi:10.3390/antibiotics14010043)

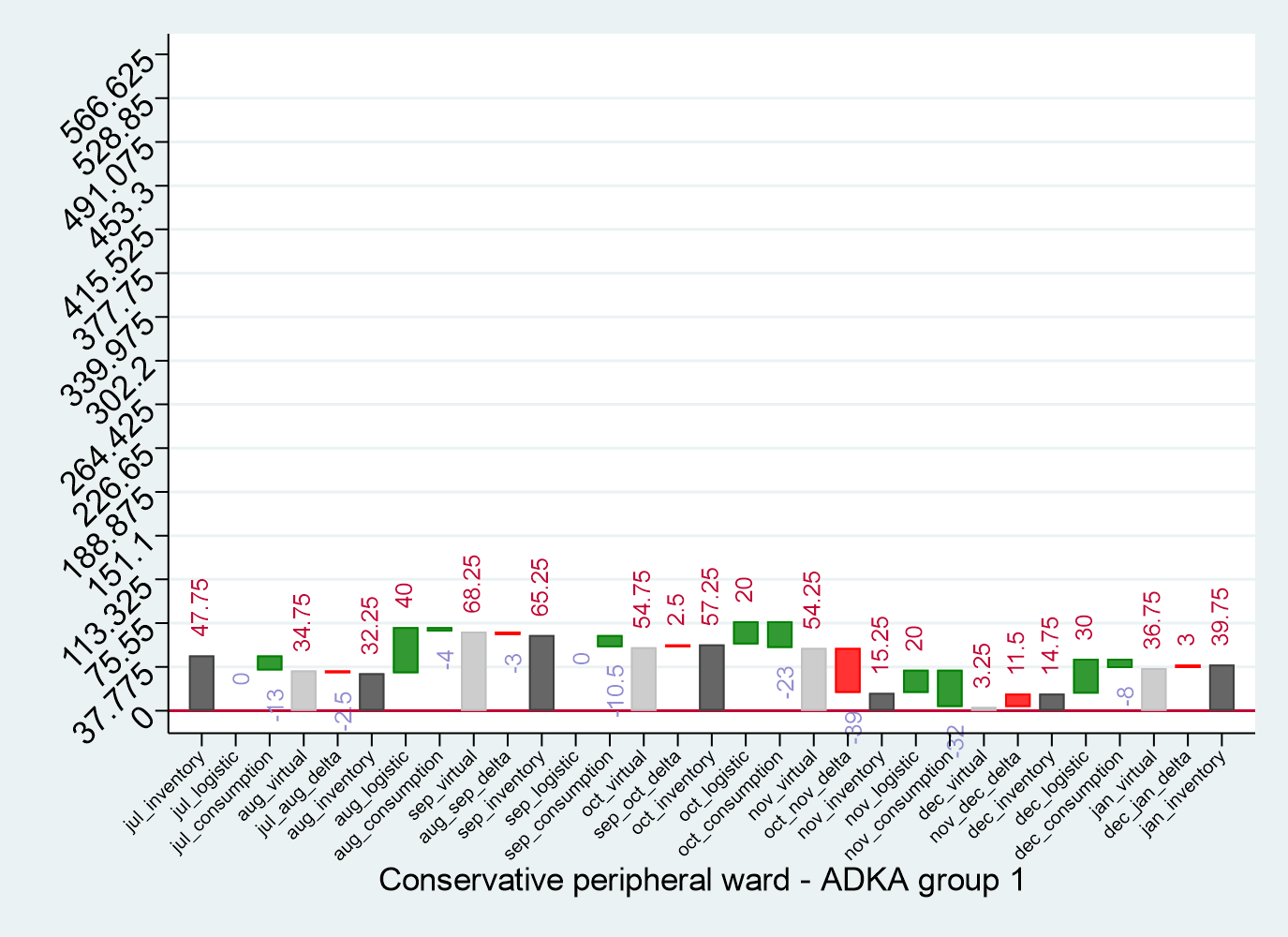

Supplement: Supplementary file 1 [file antibiotics-14-00043-s001.zip › Figure S1 - Conservative peripheral ward - ADKA group 1.png]

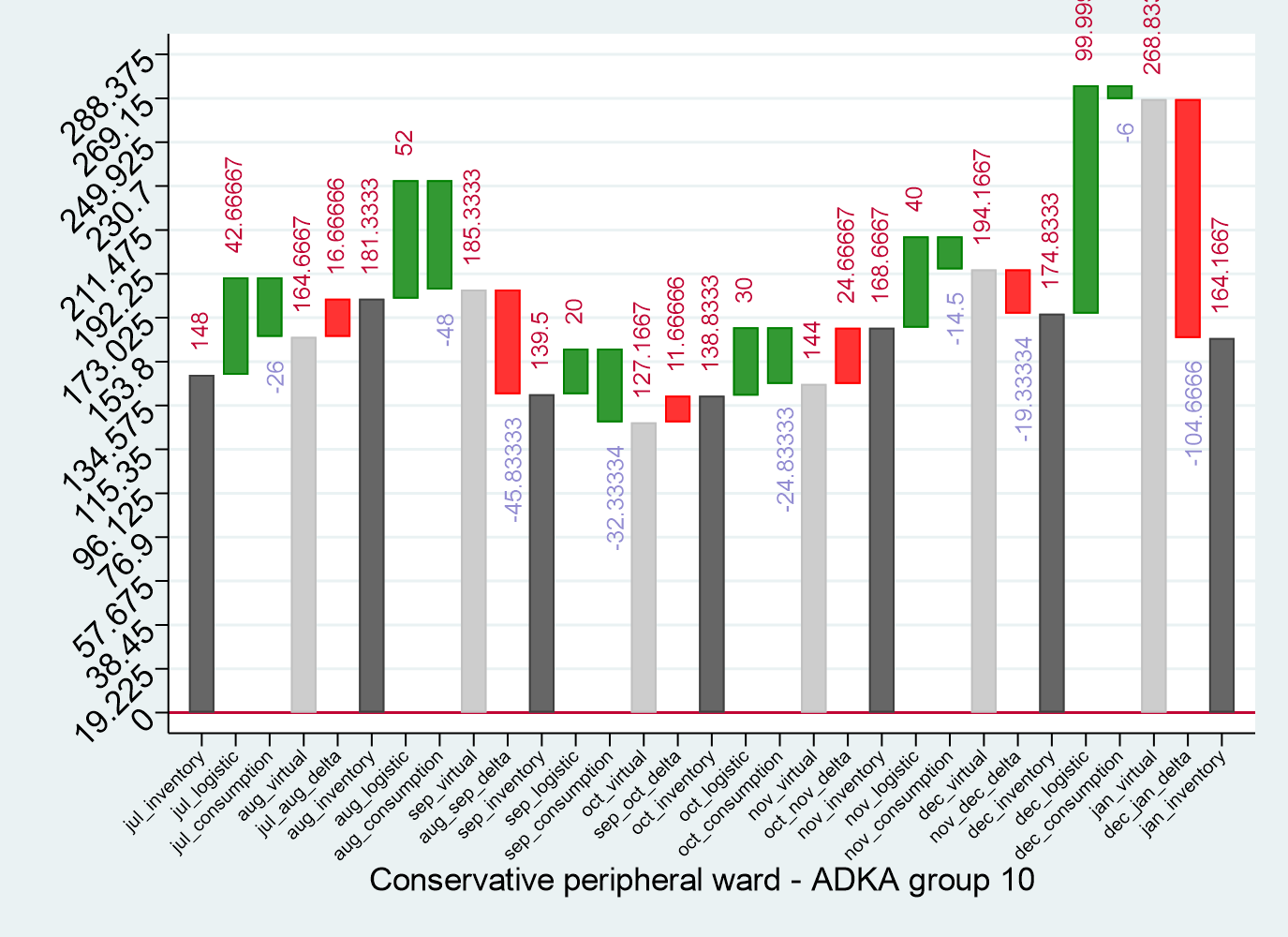

Supplement: Supplementary file 1 [file antibiotics-14-00043-s001.zip › Figure S10 - Conservative peripheral ward - ADKA group 10.png]

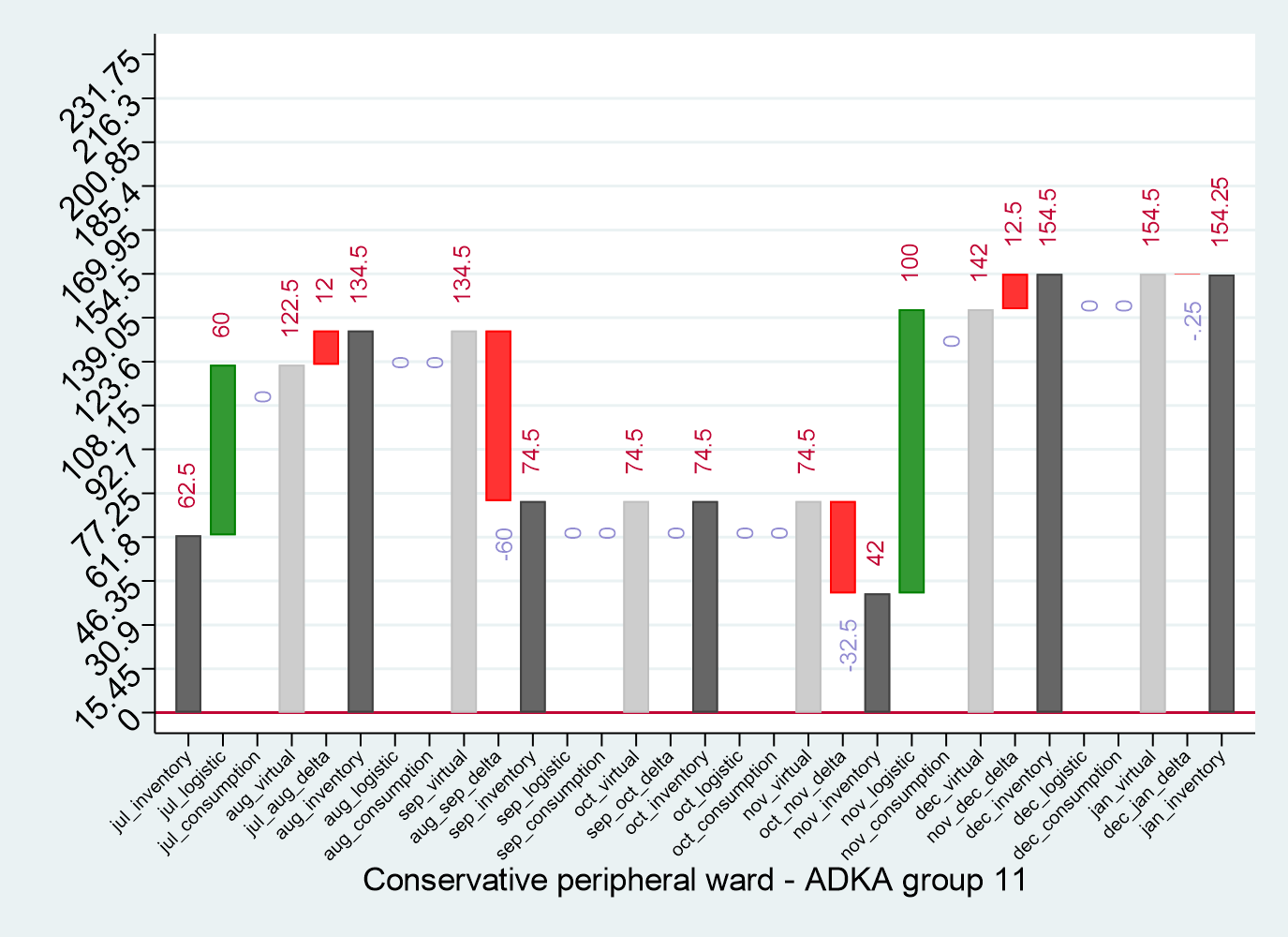

Supplement: Supplementary file 1 [file antibiotics-14-00043-s001.zip › Figure S11 - Conservative peripheral ward - ADKA group 11.png]

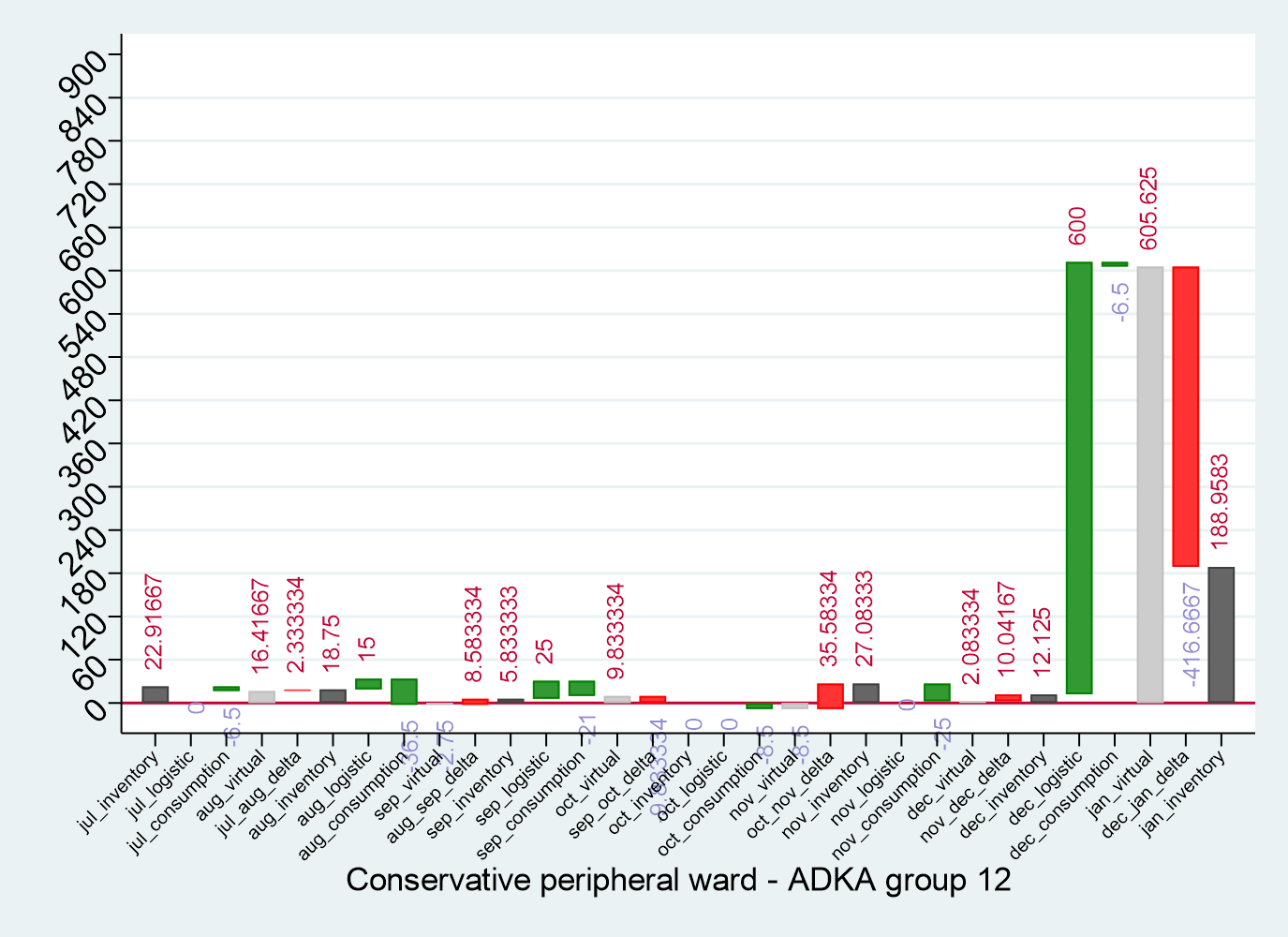

Supplement: Supplementary file 1 [file antibiotics-14-00043-s001.zip › Figure S12 - Conservative peripheral ward - ADKA group 12.png]

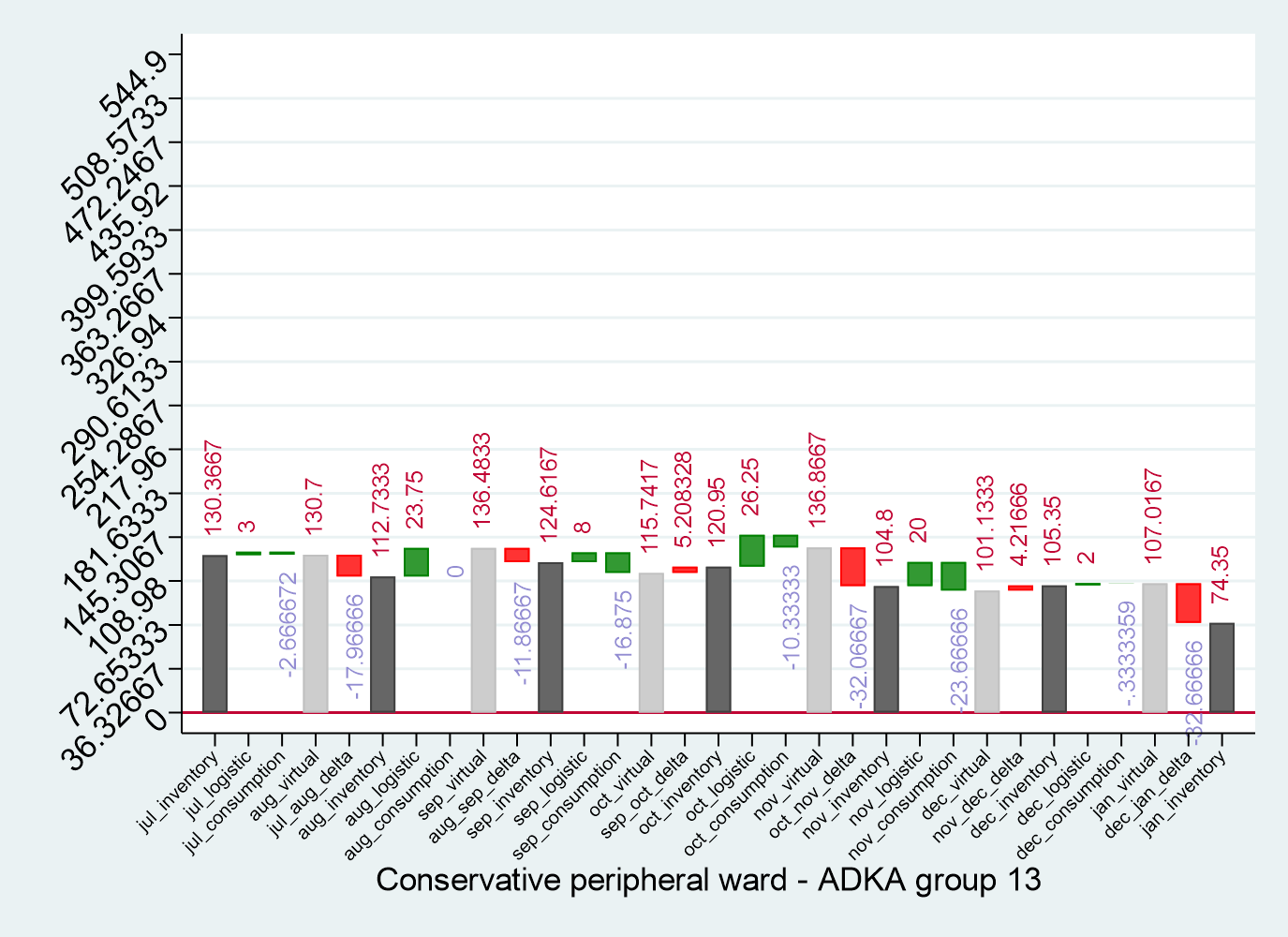

Supplement: Supplementary file 1 [file antibiotics-14-00043-s001.zip › Figure S13 - Conservative peripheral ward - ADKA group 13.png]

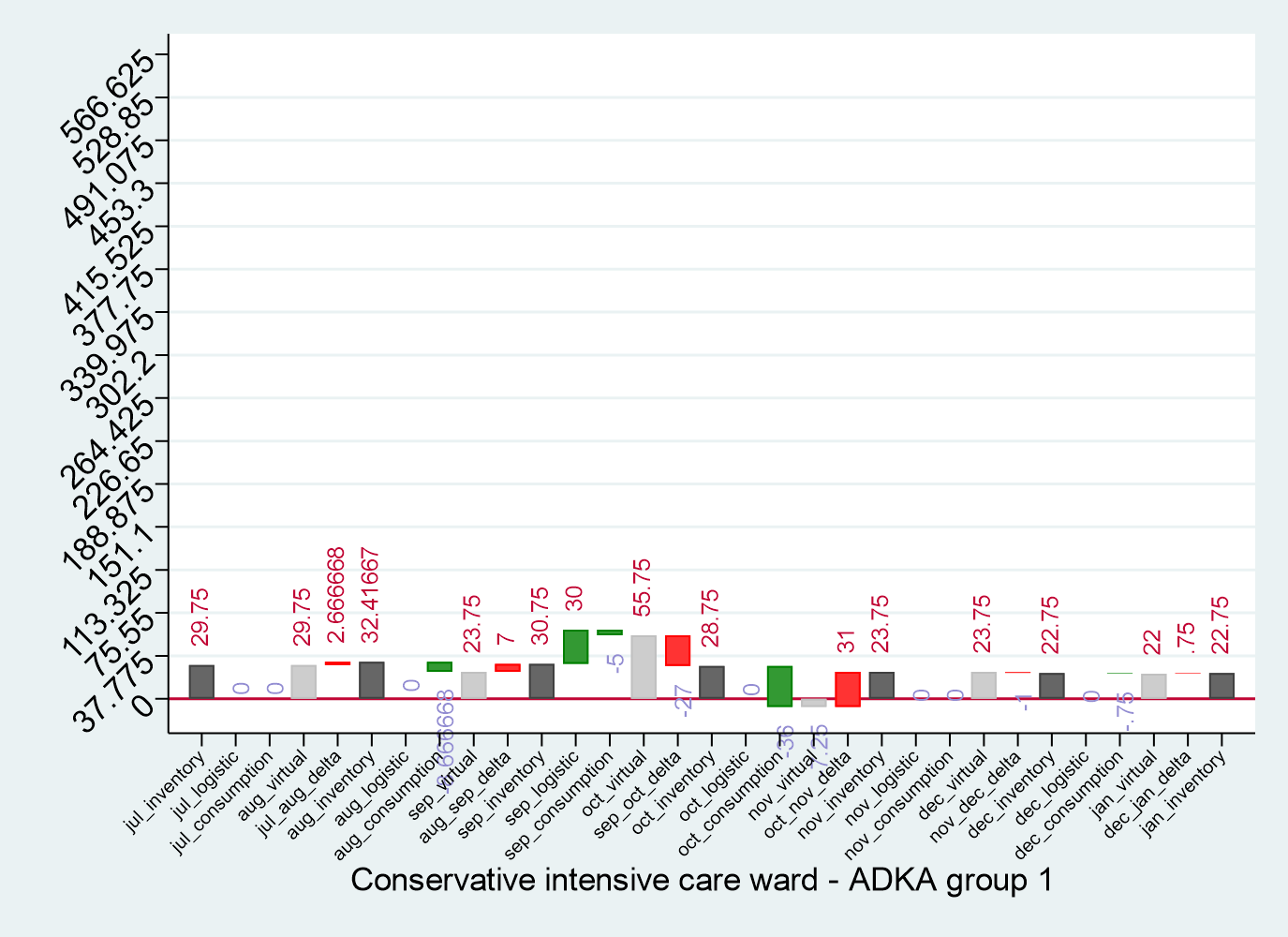

Supplement: Supplementary file 1 [file antibiotics-14-00043-s001.zip › Figure S14 - Conservative intensive care ward - ADKA group 1.png]

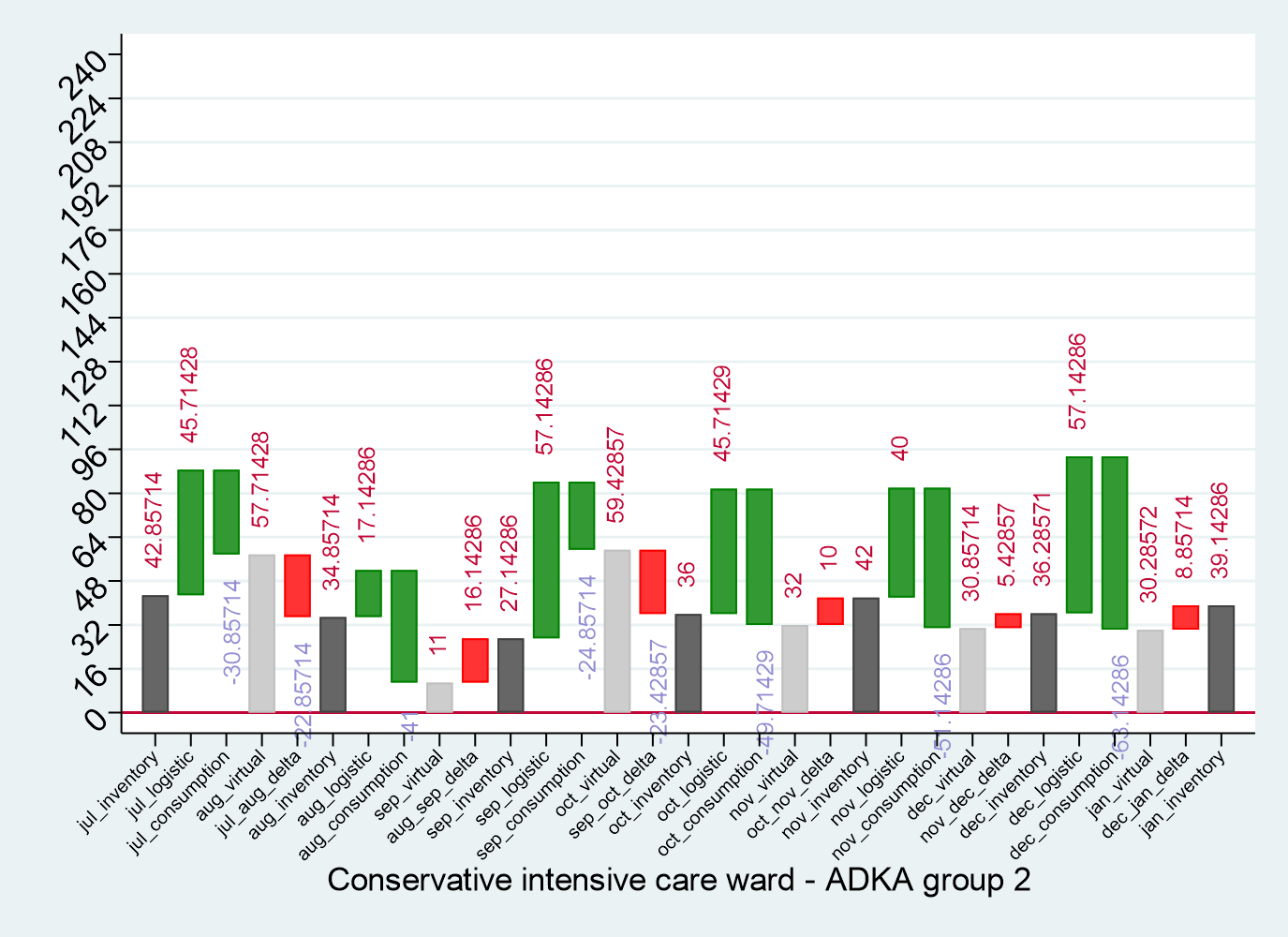

Supplement: Supplementary file 1 [file antibiotics-14-00043-s001.zip › Figure S15 - Conservative intensive care ward - ADKA group 2.png]

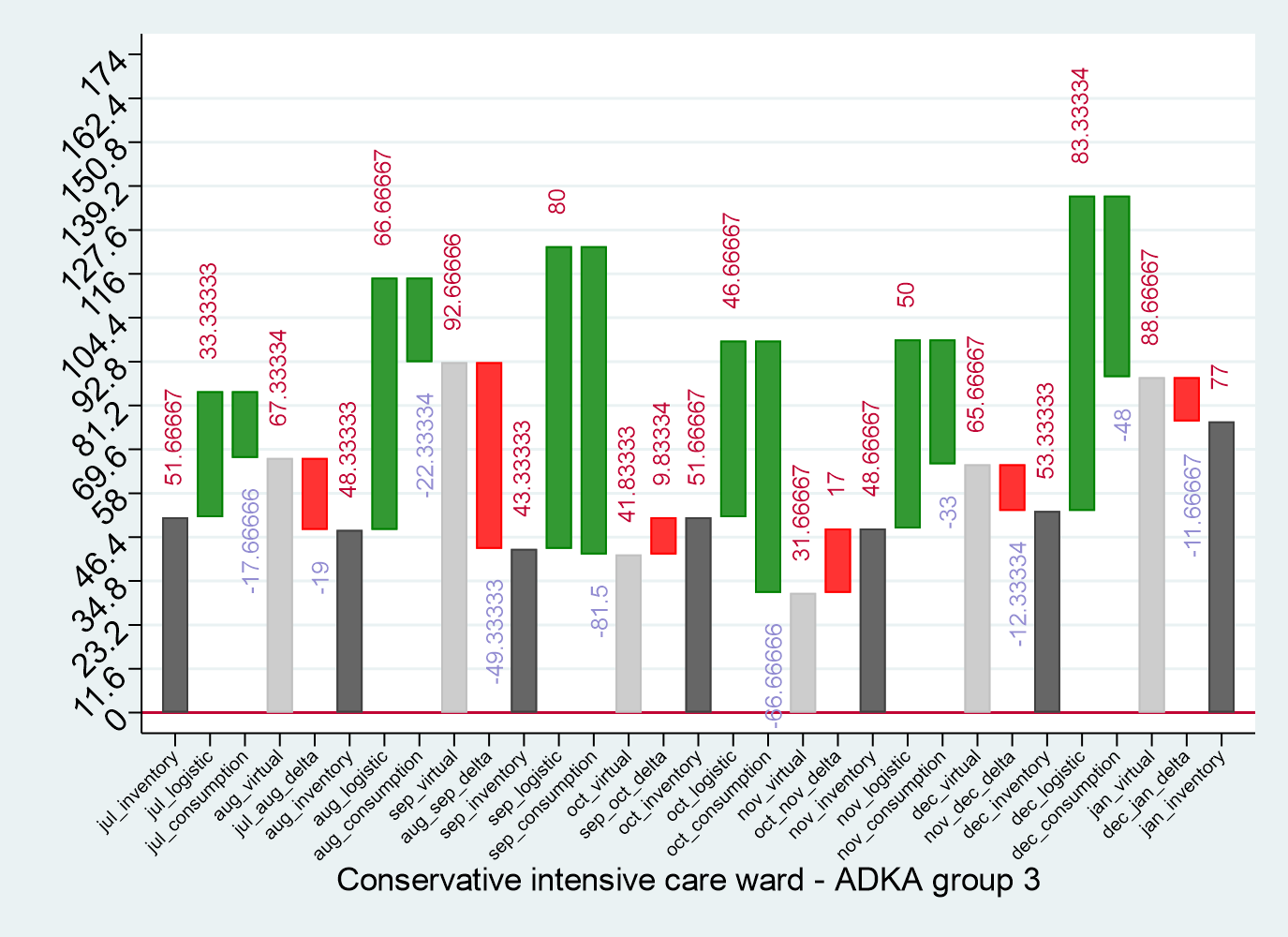

Supplement: Supplementary file 1 [file antibiotics-14-00043-s001.zip › Figure S16 - Conservative intensive care ward - ADKA group 3.png]

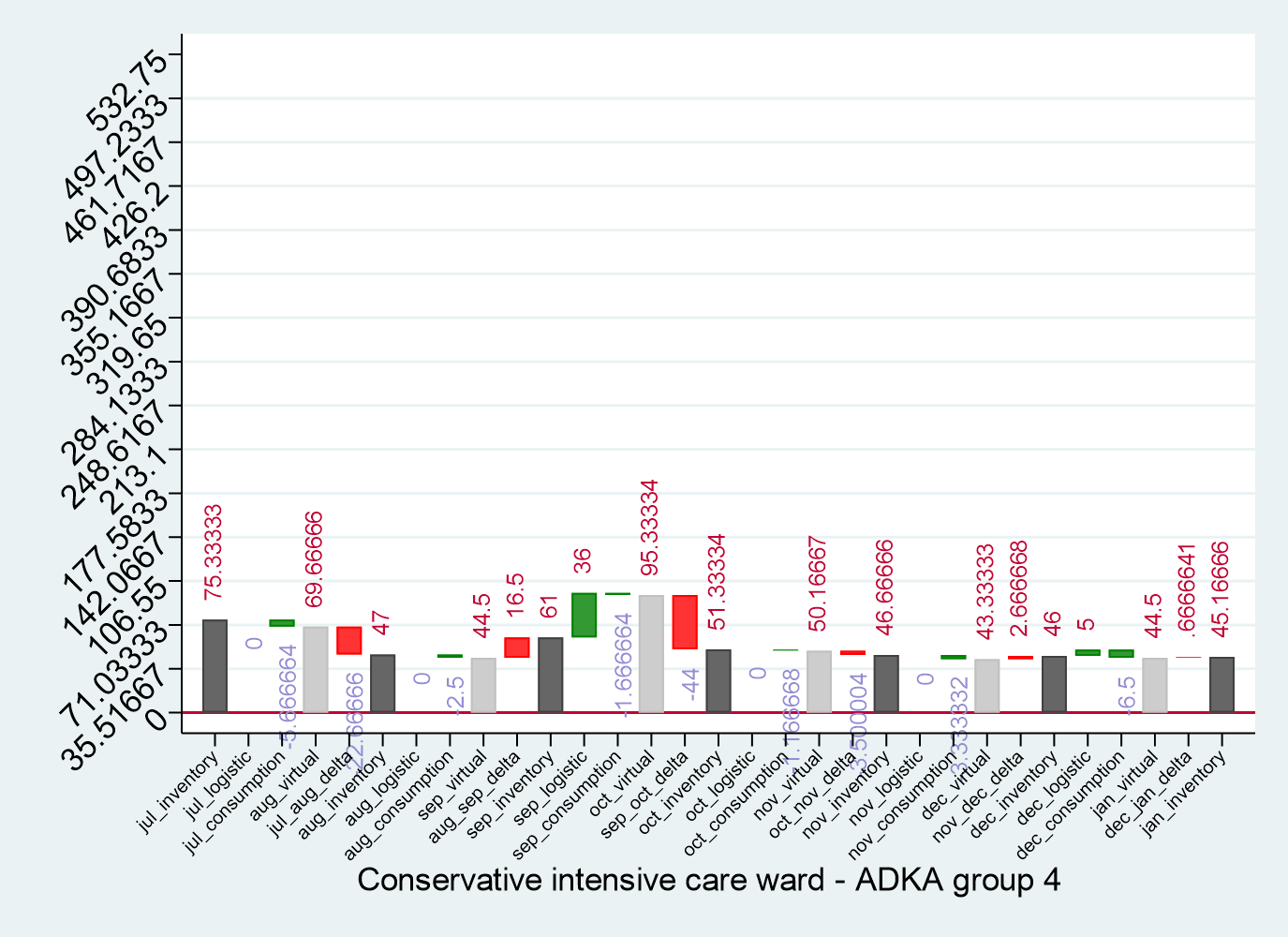

Supplement: Supplementary file 1 [file antibiotics-14-00043-s001.zip › Figure S17 - Conservative intensive care ward - ADKA group 4.png]

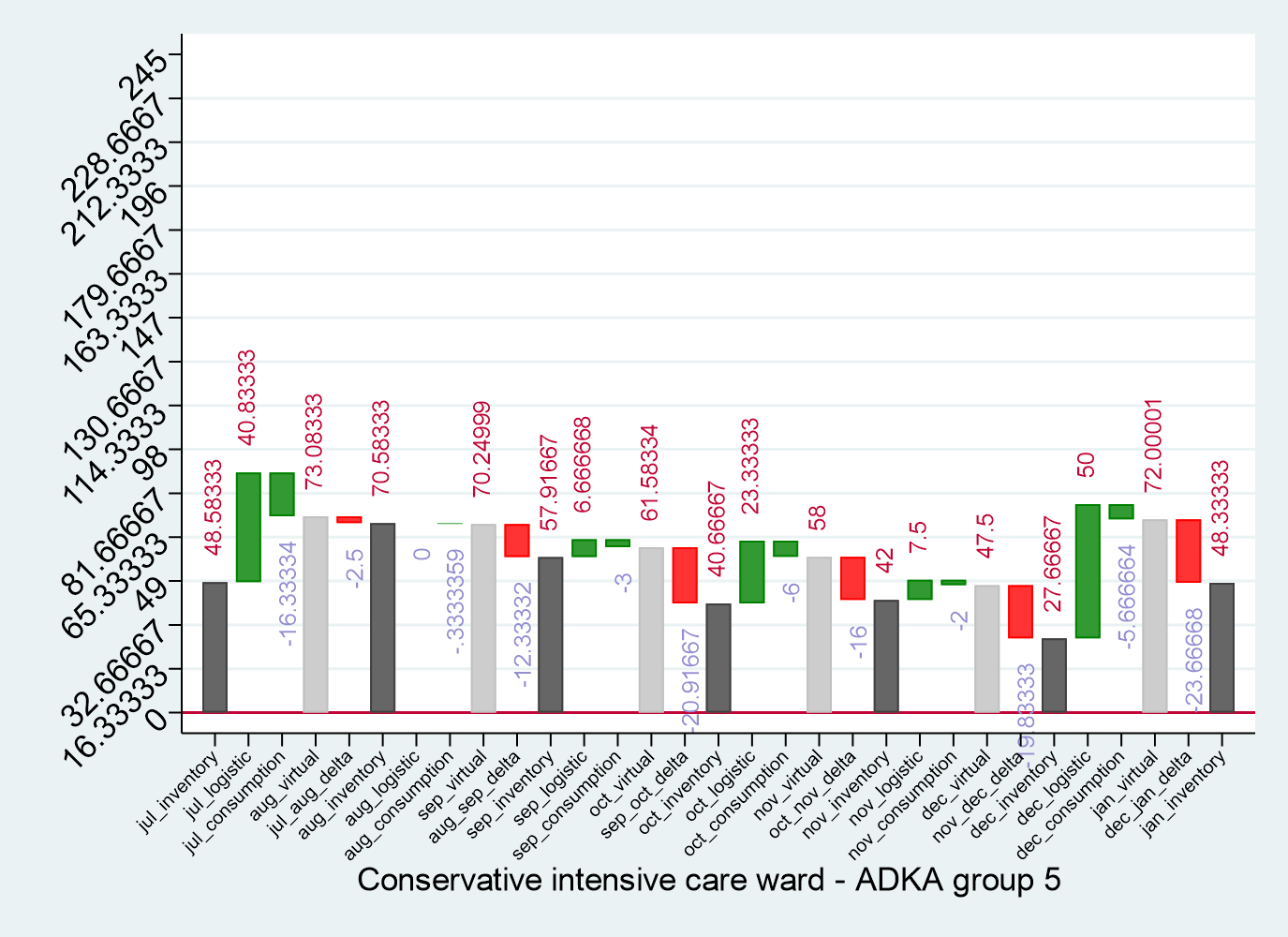

Supplement: Supplementary file 1 [file antibiotics-14-00043-s001.zip › Figure S18 - Conservative intensive care ward - ADKA group 5.png]

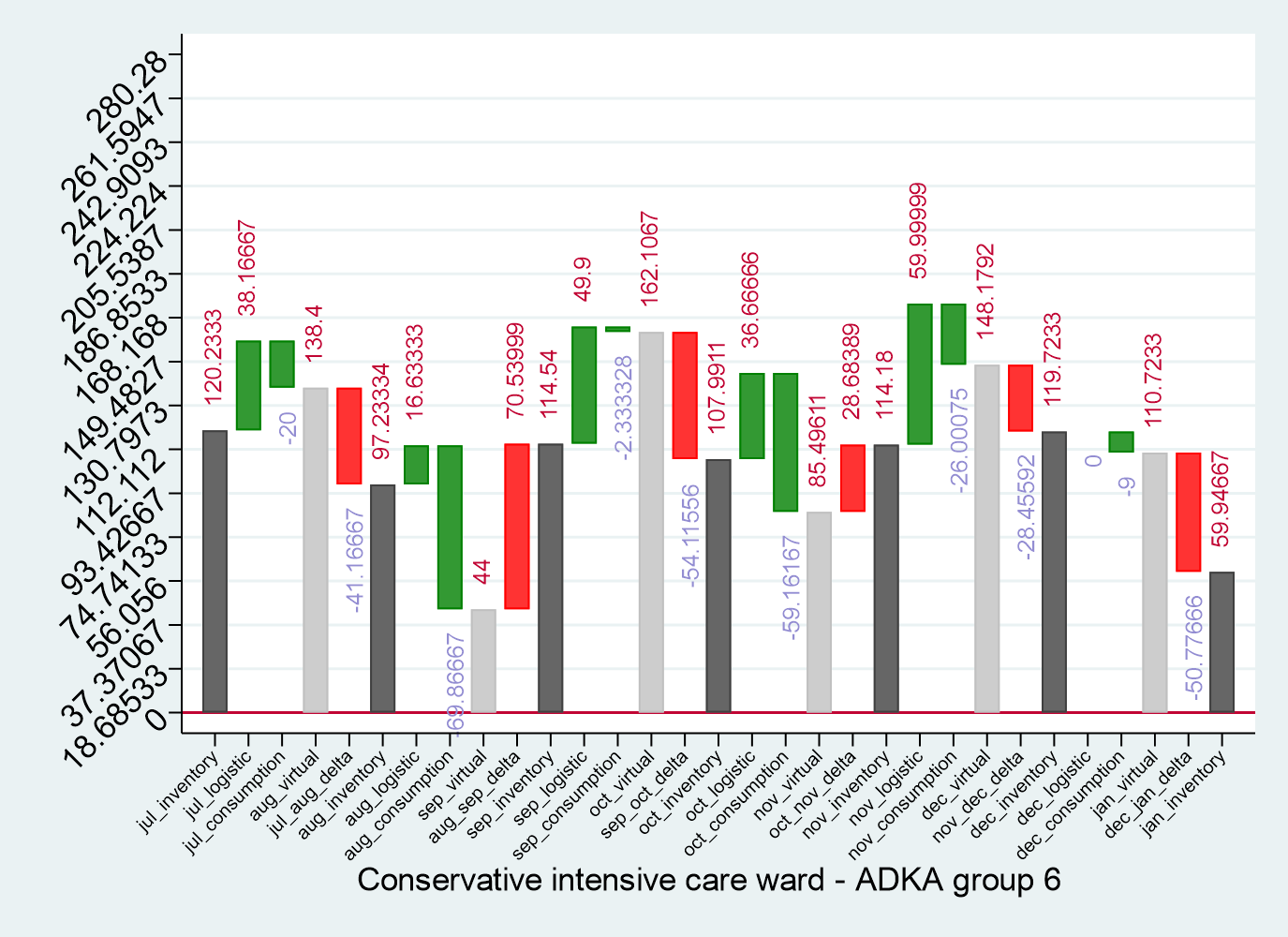

Supplement: Supplementary file 1 [file antibiotics-14-00043-s001.zip › Figure S19 - Conservative intensive care ward - ADKA group 6.png]

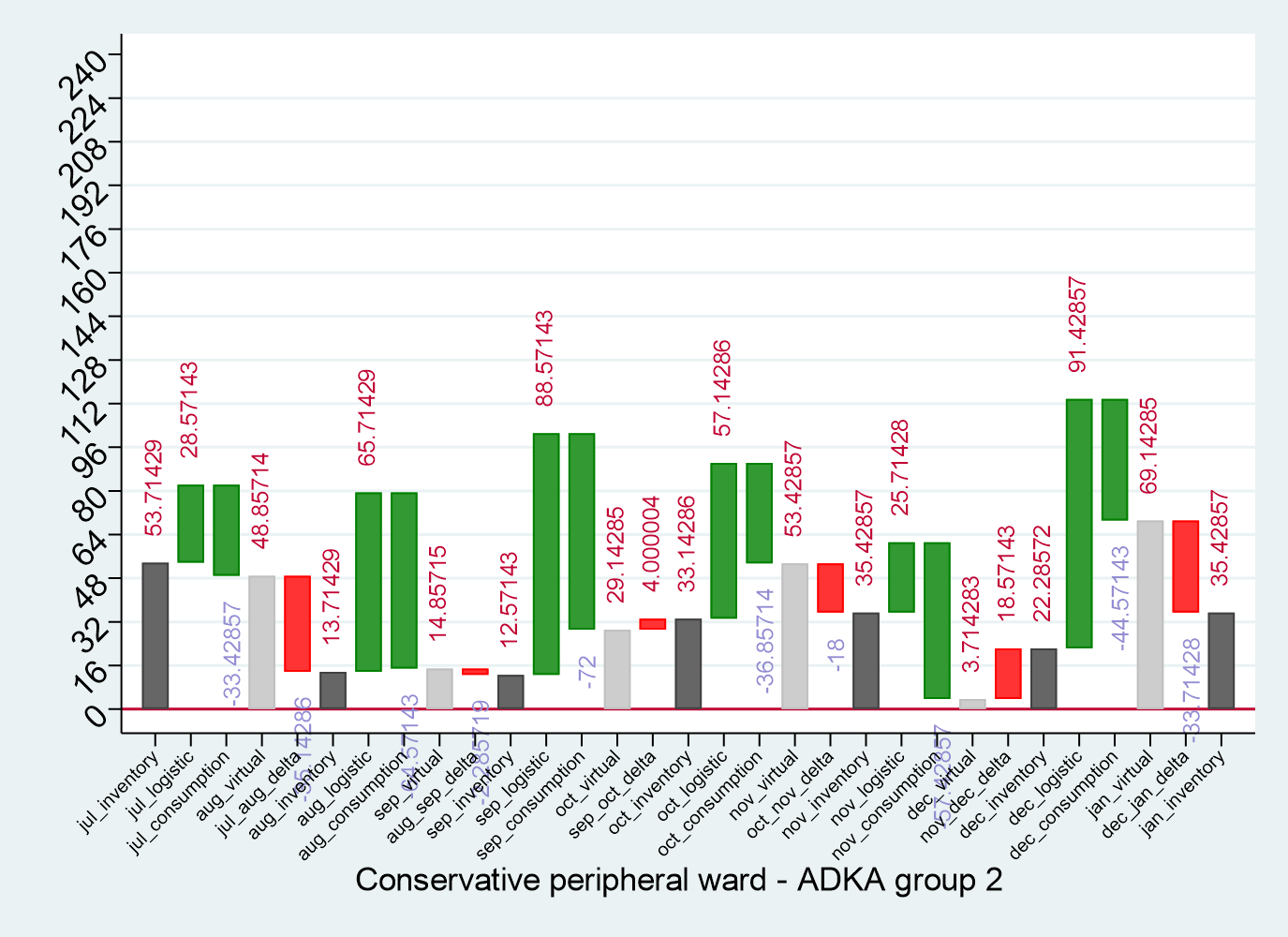

Supplement: Supplementary file 1 [file antibiotics-14-00043-s001.zip › Figure S2 - Conservative peripheral ward - ADKA group 2.png]

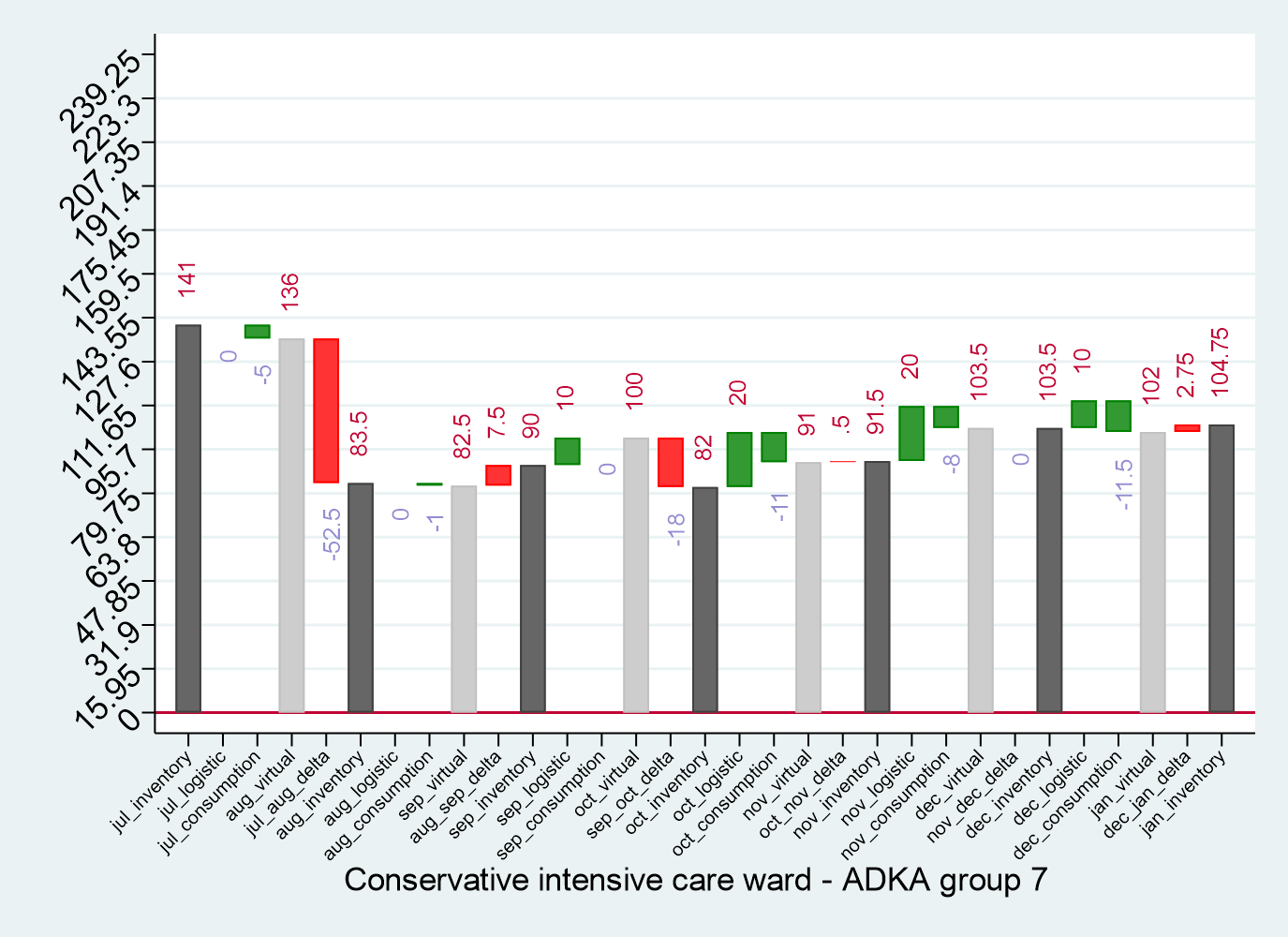

Supplement: Supplementary file 1 [file antibiotics-14-00043-s001.zip › Figure S20 - Conservative intensive care ward - ADKA group 7.png]

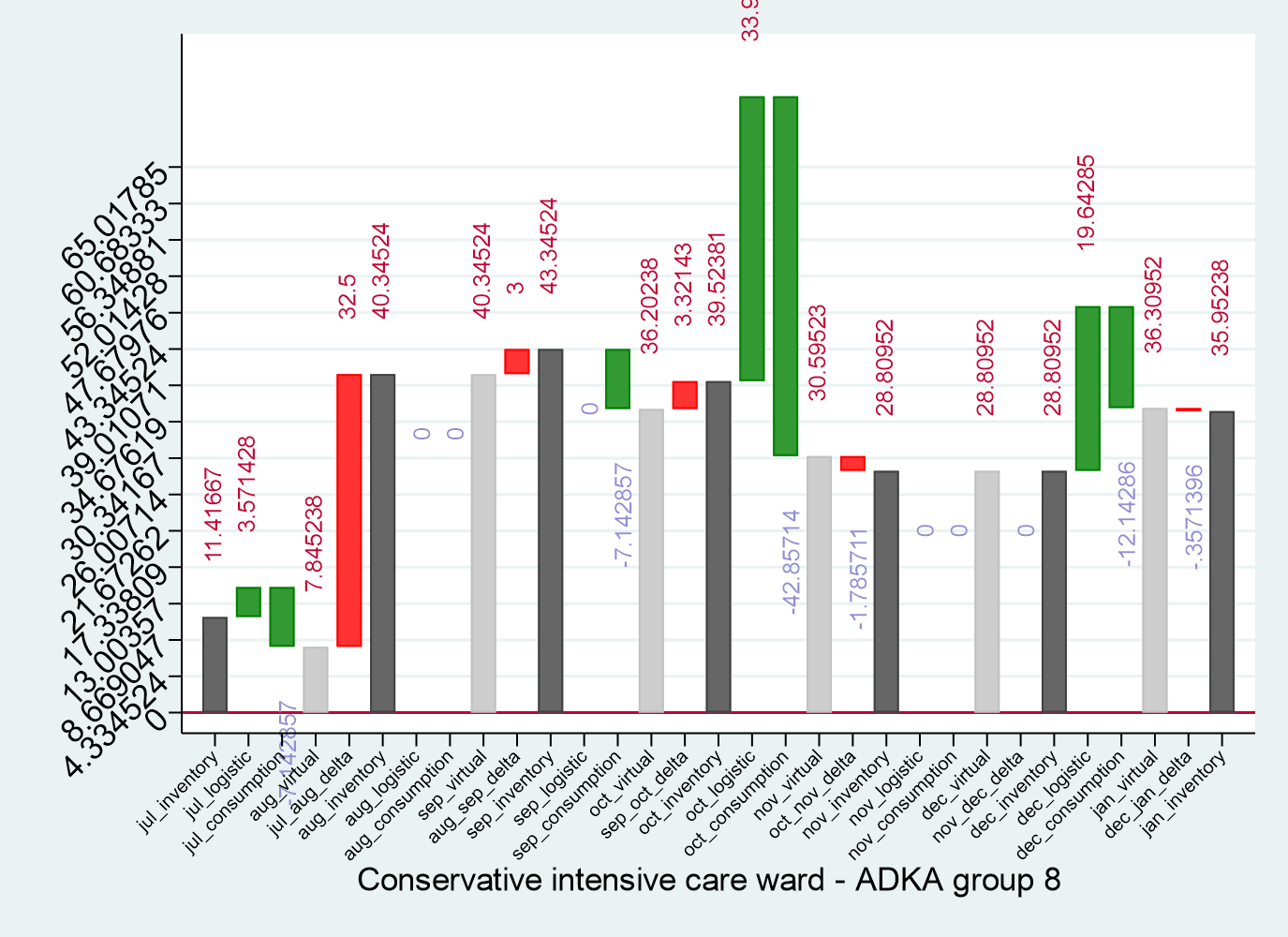

Supplement: Supplementary file 1 [file antibiotics-14-00043-s001.zip › Figure S21 - Conservative intensive care ward - ADKA group 8.png]

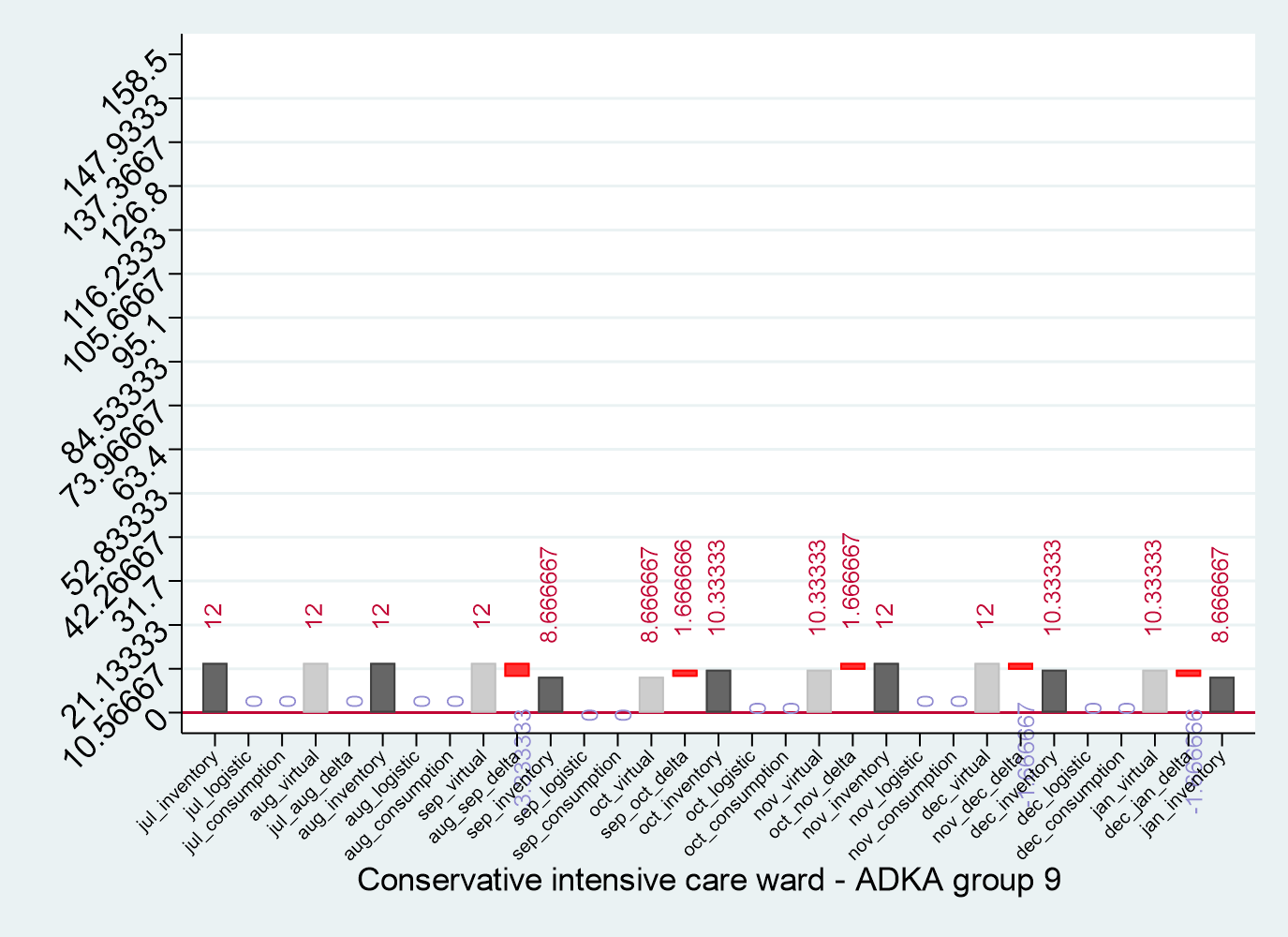

Supplement: Supplementary file 1 [file antibiotics-14-00043-s001.zip › Figure S22 - Conservative intensive care ward - ADKA group 9.png]

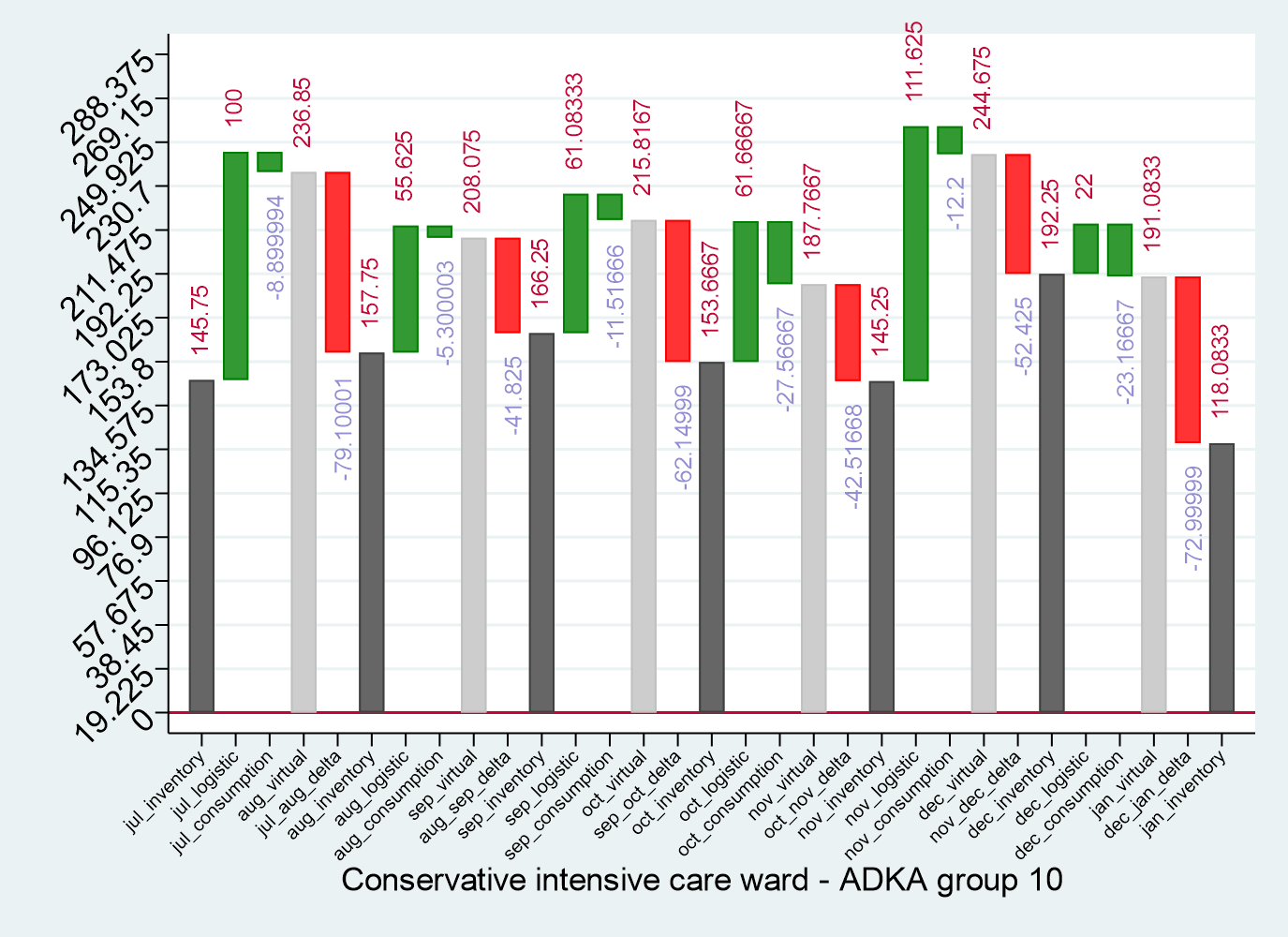

Supplement: Supplementary file 1 [file antibiotics-14-00043-s001.zip › Figure S23 - Conservative intensive care ward - ADKA group 10.png]

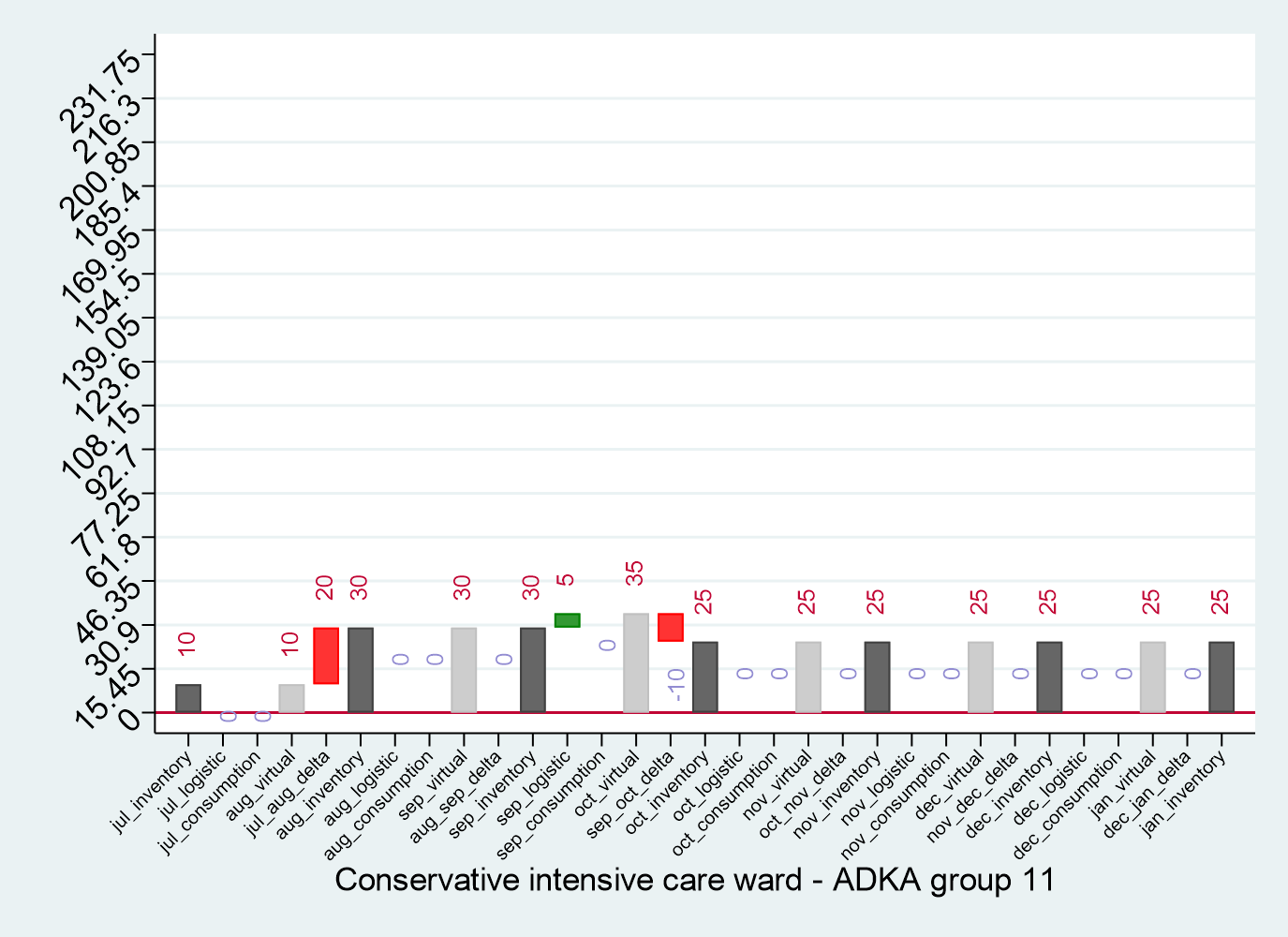

Supplement: Supplementary file 1 [file antibiotics-14-00043-s001.zip › Figure S24 - Conservative intensive care ward - ADKA group 11.png]

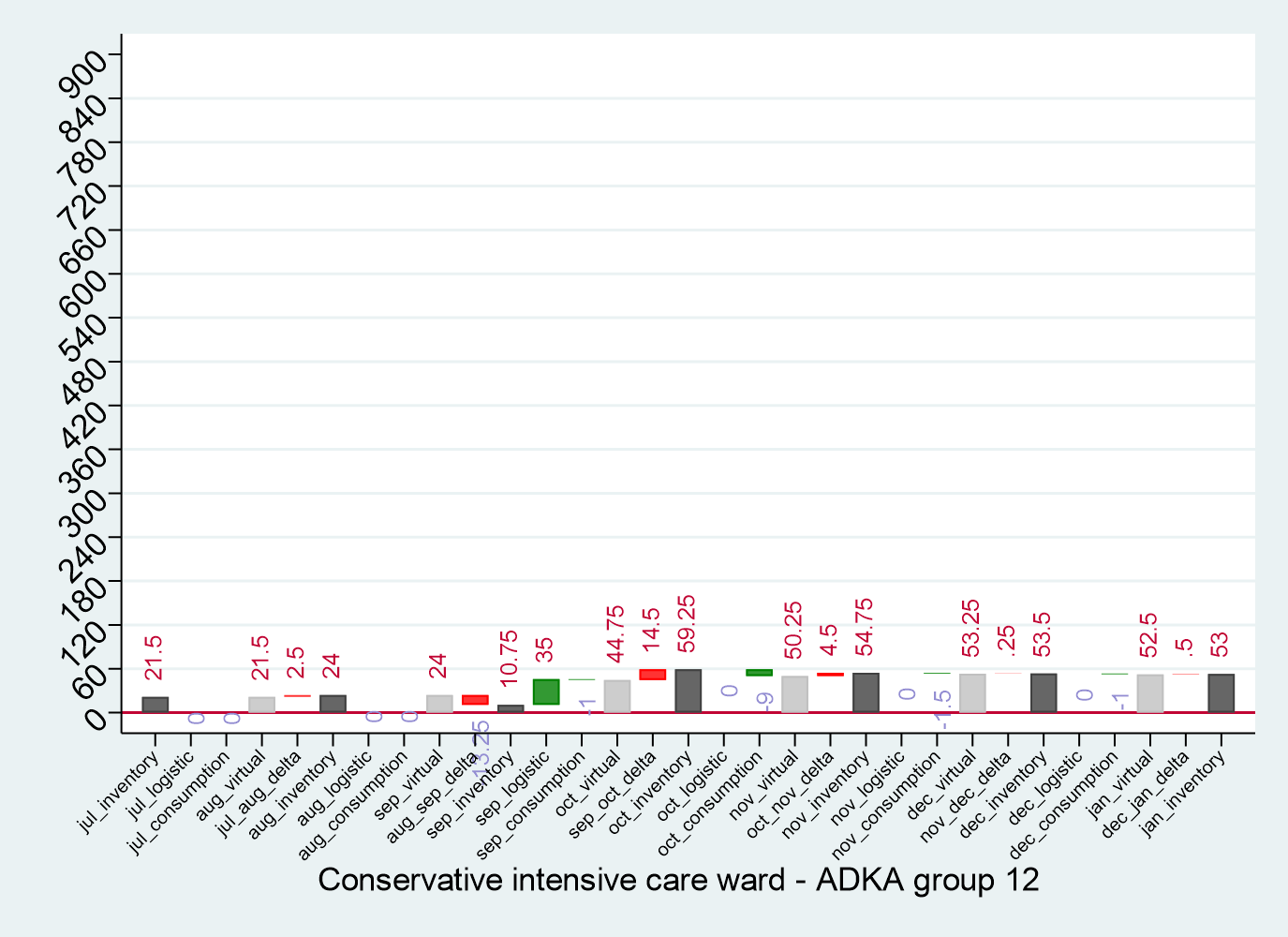

Supplement: Supplementary file 1 [file antibiotics-14-00043-s001.zip › Figure S25 - Conservative intensive care ward - ADKA group 12.png]

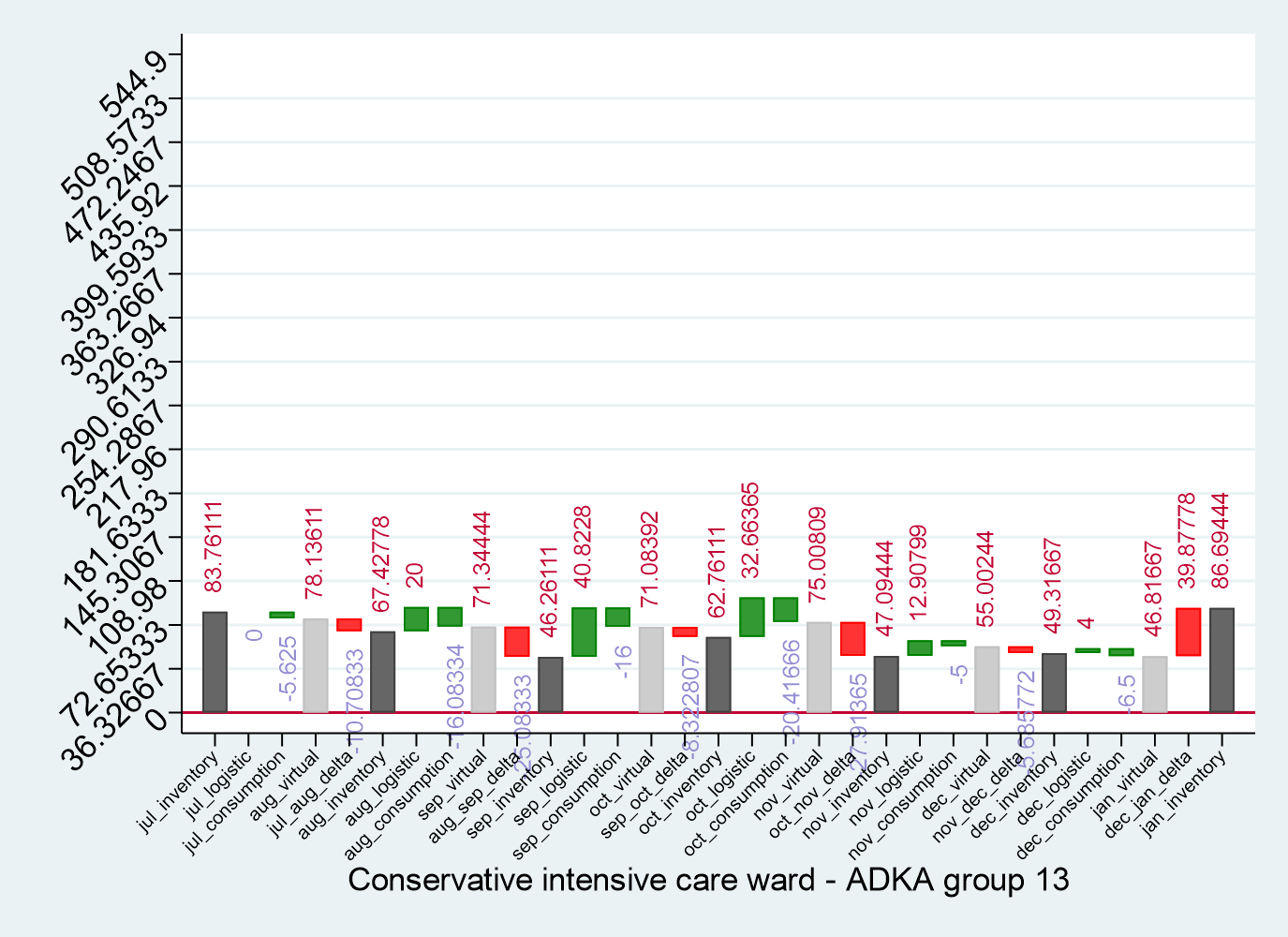

Supplement: Supplementary file 1 [file antibiotics-14-00043-s001.zip › Figure S26 - Conservative intensive care ward - ADKA group 13.png]

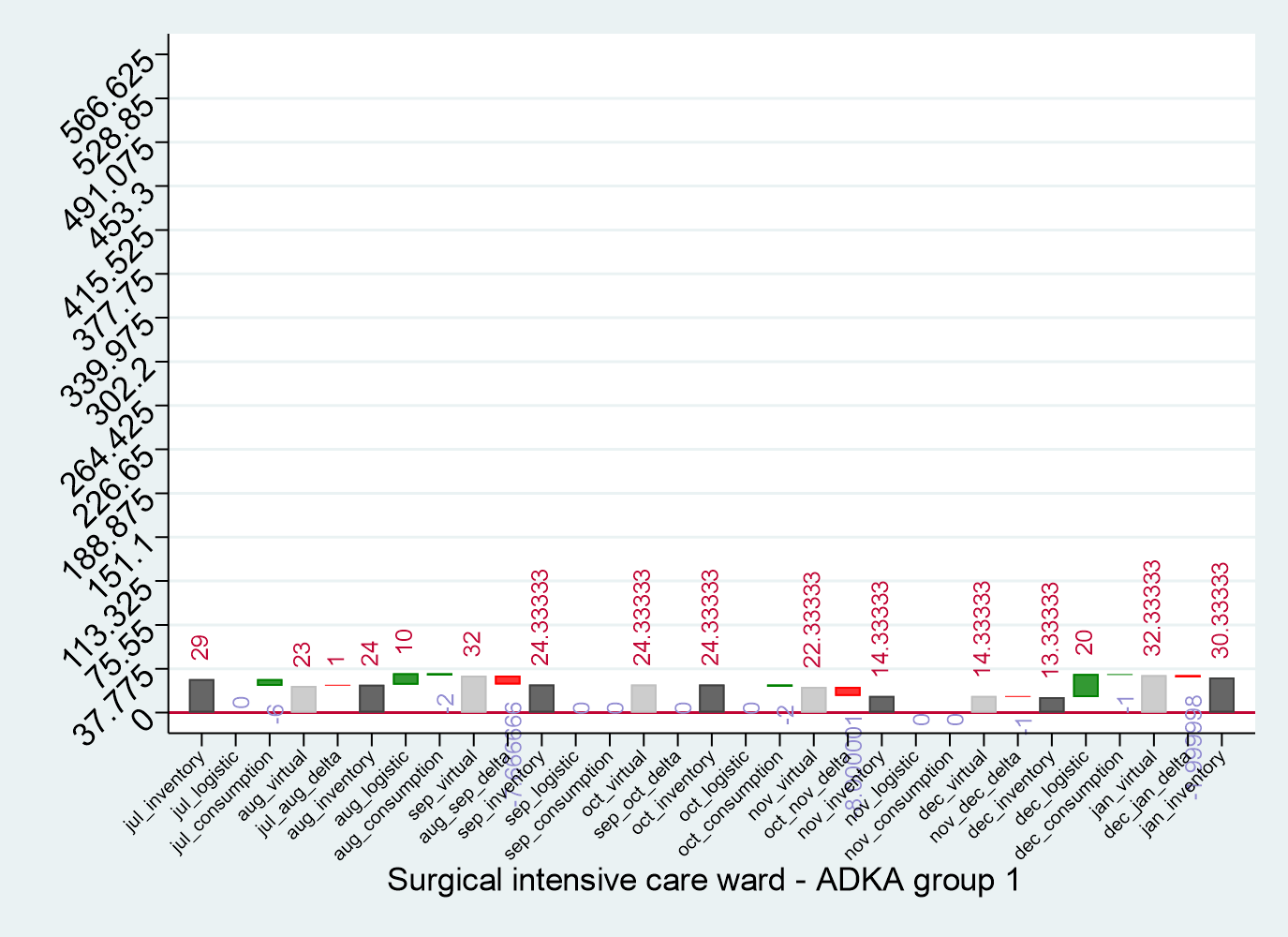

Supplement: Supplementary file 1 [file antibiotics-14-00043-s001.zip › Figure S27 - Surgical intensive care ward - ADKA group 1.png]

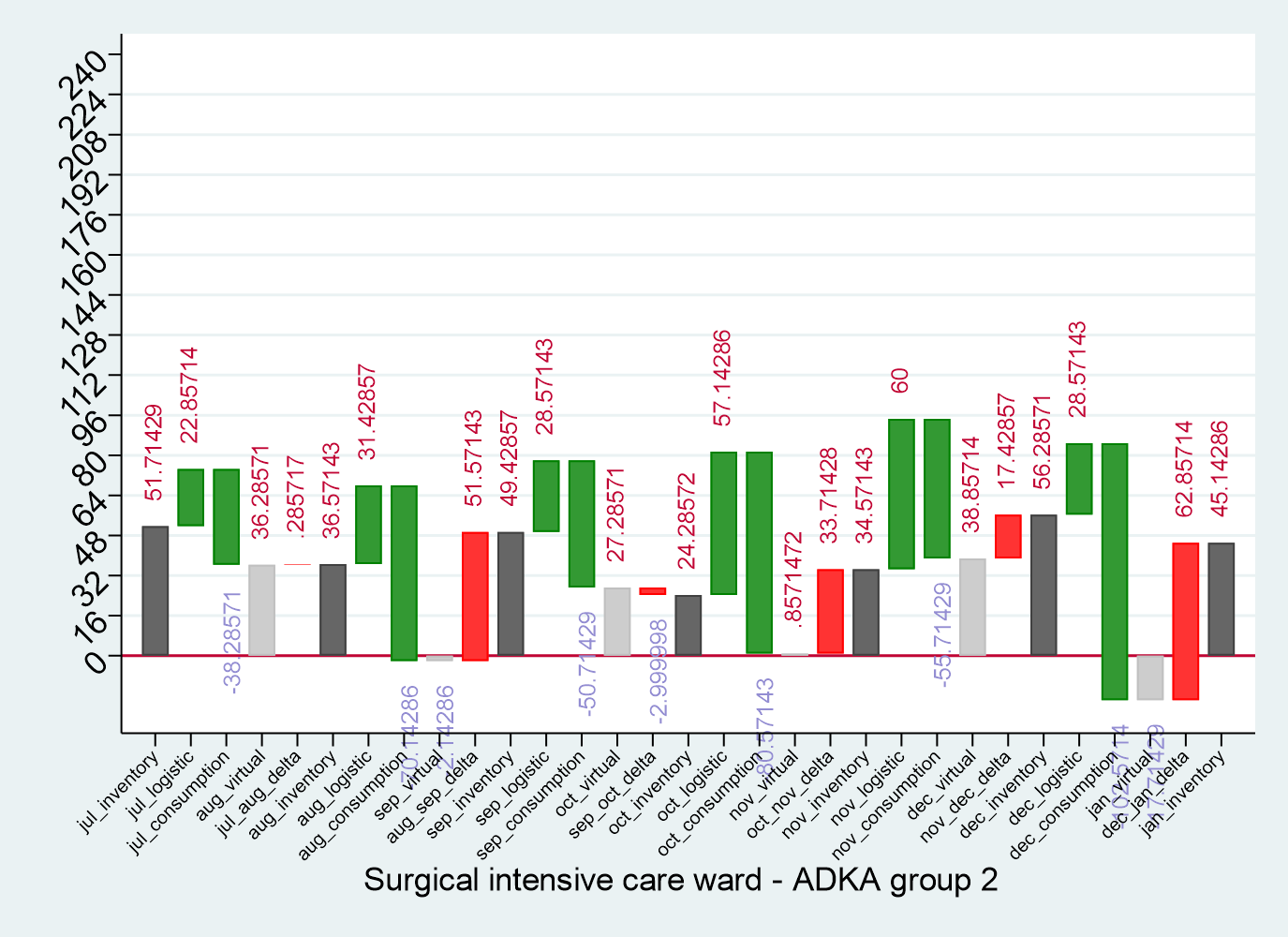

Supplement: Supplementary file 1 [file antibiotics-14-00043-s001.zip › Figure S28 - Surgical intensive care ward - ADKA group 2.png]

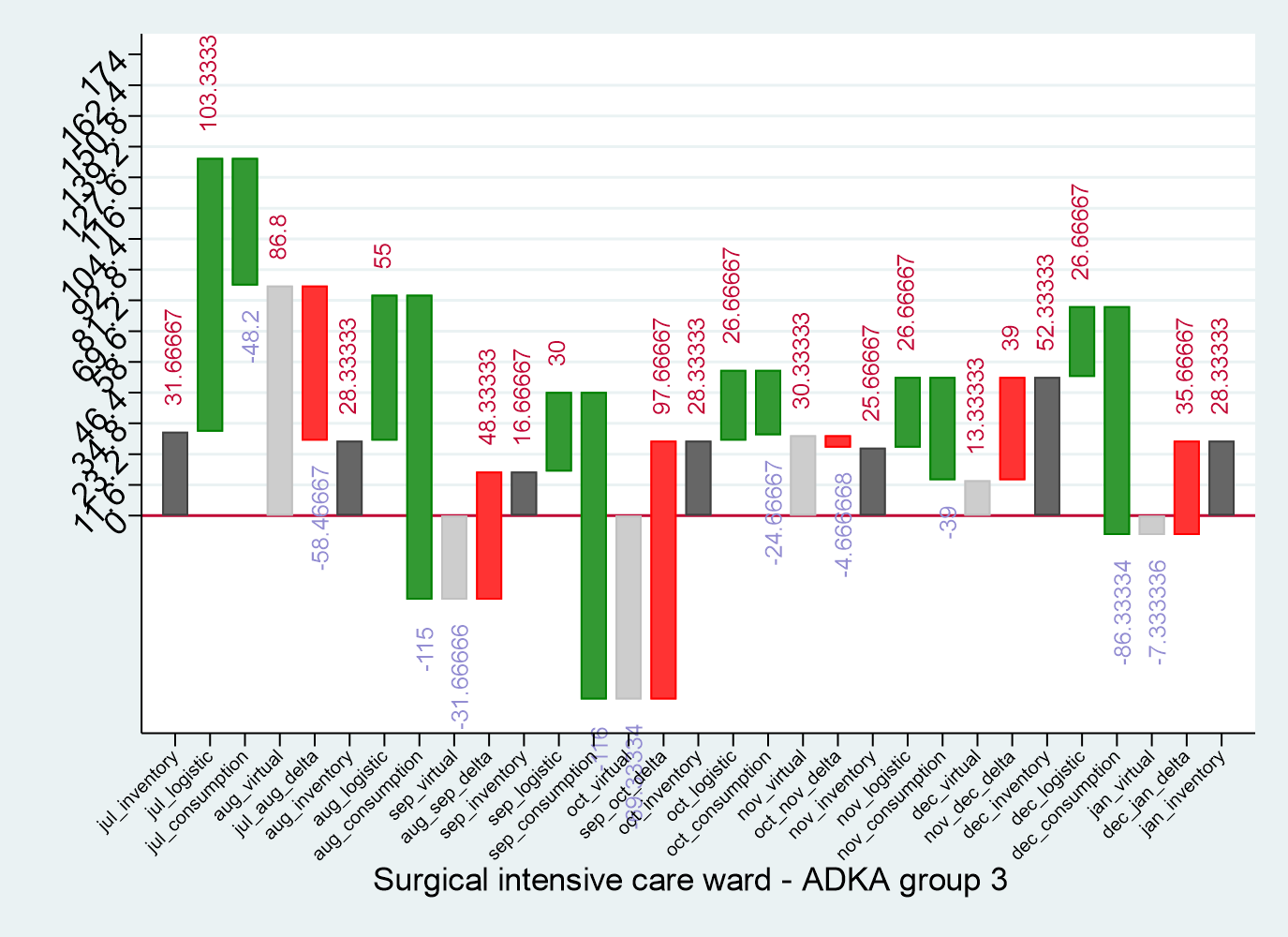

Supplement: Supplementary file 1 [file antibiotics-14-00043-s001.zip › Figure S29 - Surgical intensive care ward - ADKA group 3.png]

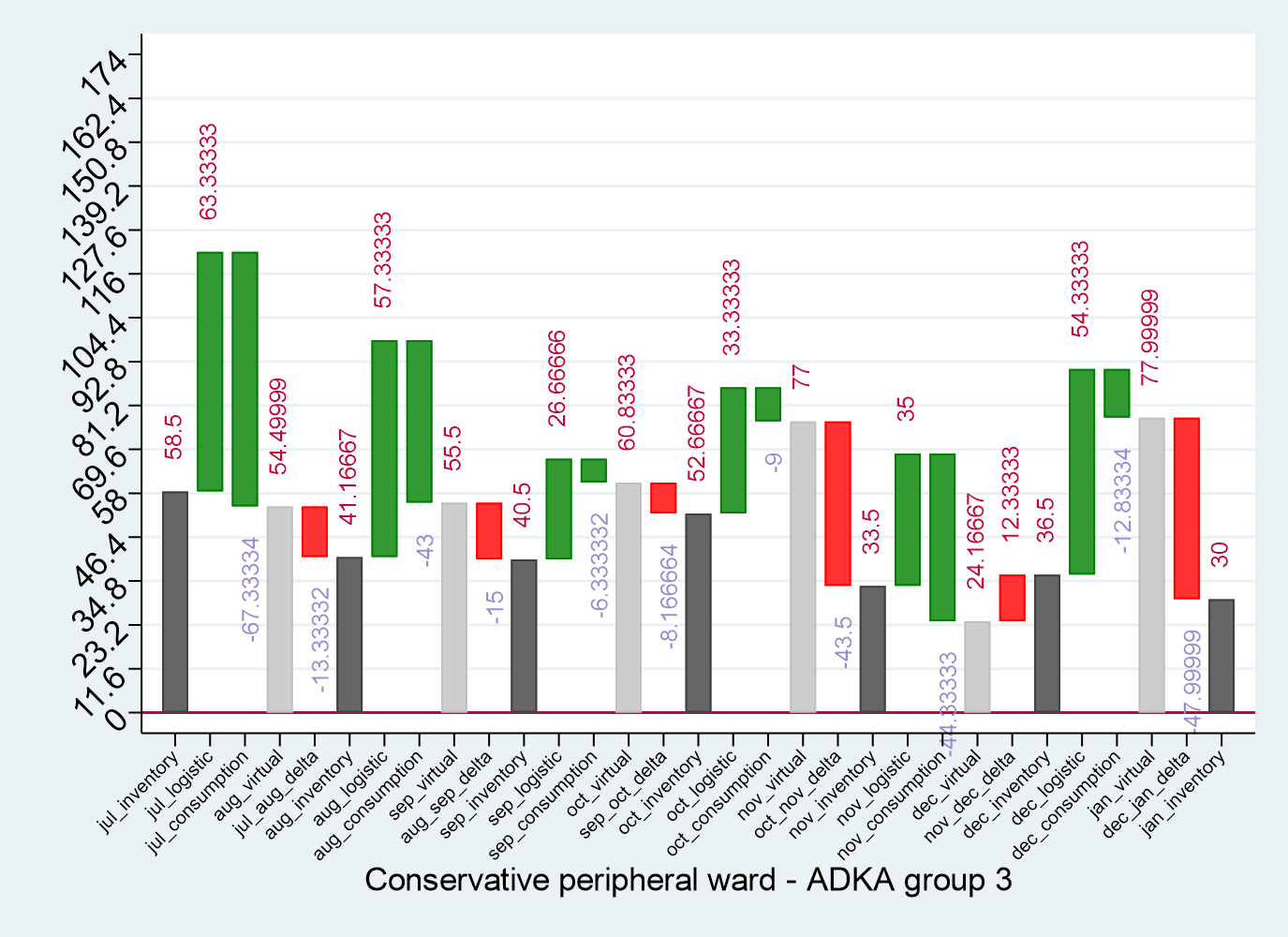

Supplement: Supplementary file 1 [file antibiotics-14-00043-s001.zip › Figure S3 - Conservative peripheral ward - ADKA group 3.png]

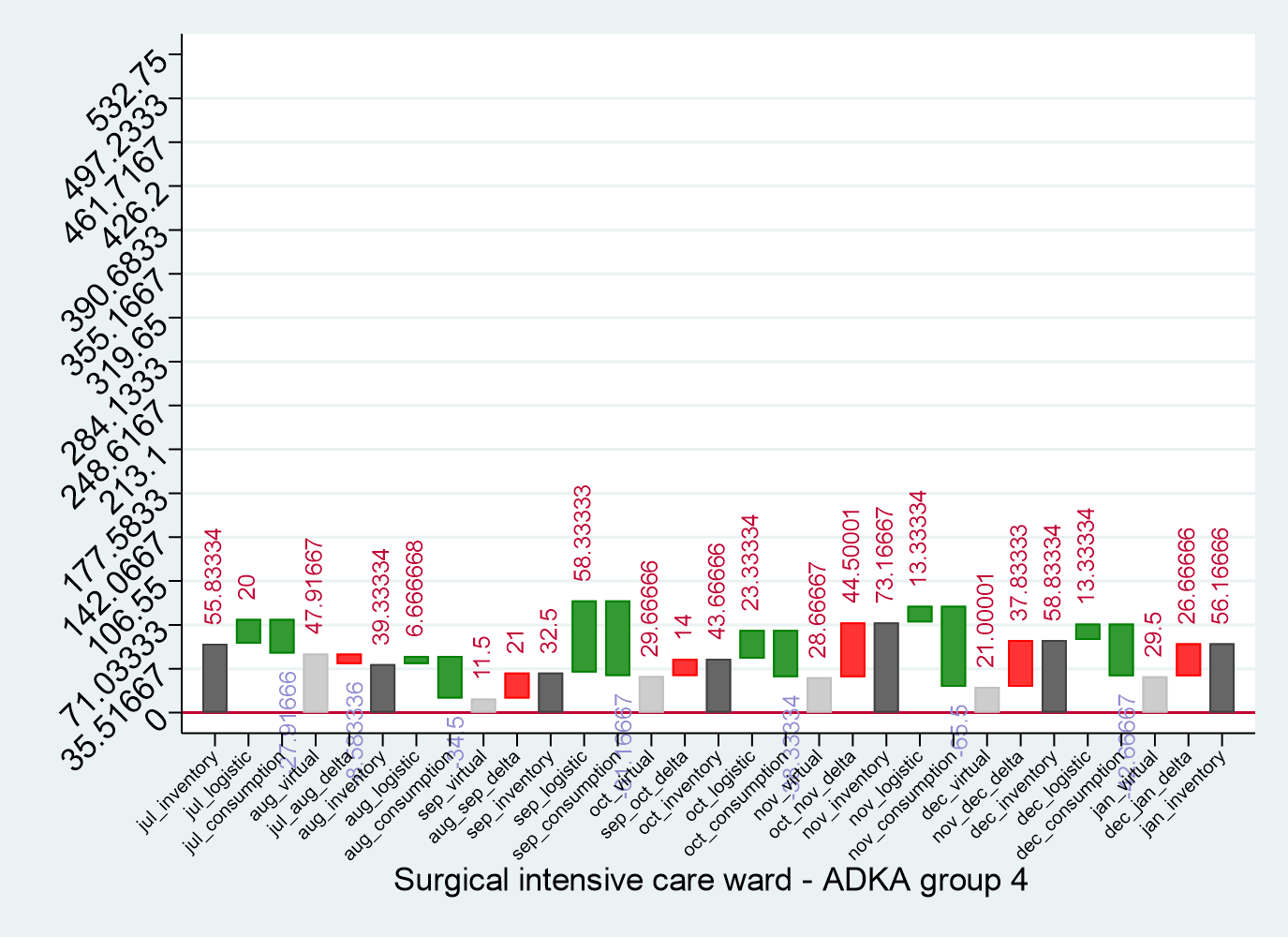

Supplement: Supplementary file 1 [file antibiotics-14-00043-s001.zip › Figure S30 - Surgical intensive care ward - ADKA group 4.png]

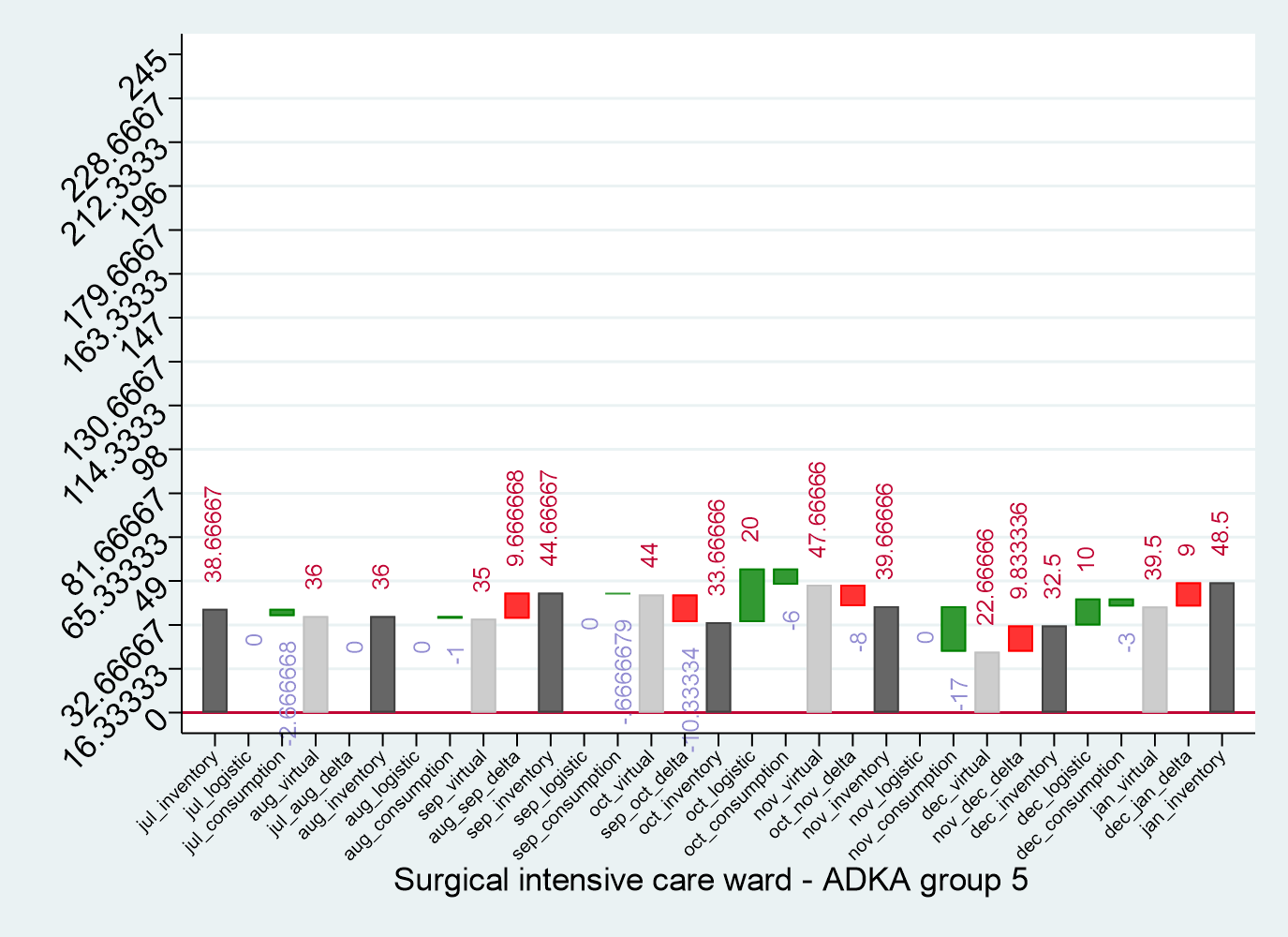

Supplement: Supplementary file 1 [file antibiotics-14-00043-s001.zip › Figure S31 - Surgical intensive care ward - ADKA group 5.png]

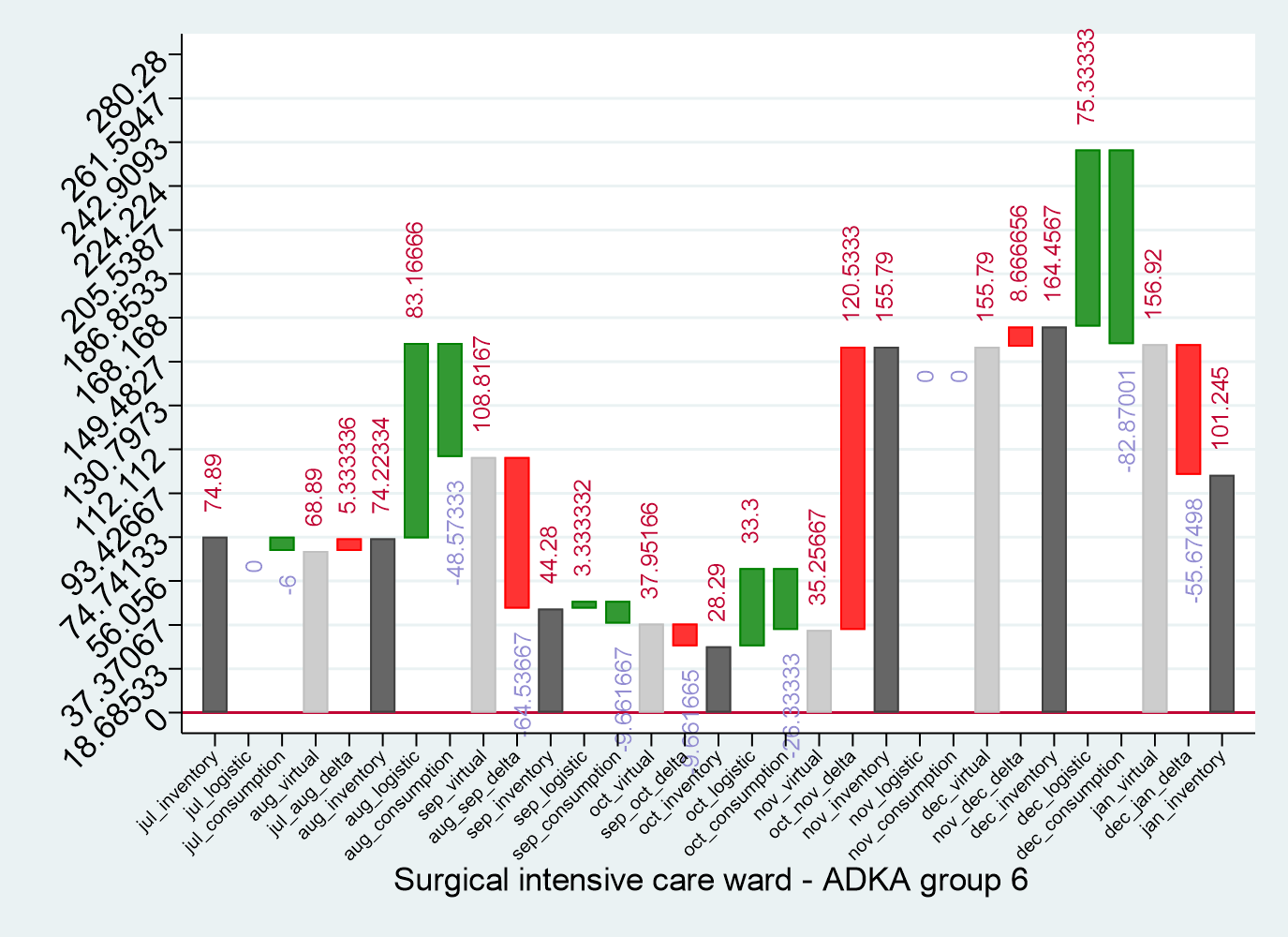

Supplement: Supplementary file 1 [file antibiotics-14-00043-s001.zip › Figure S32 - Surgical intensive care ward - ADKA group 6.png]

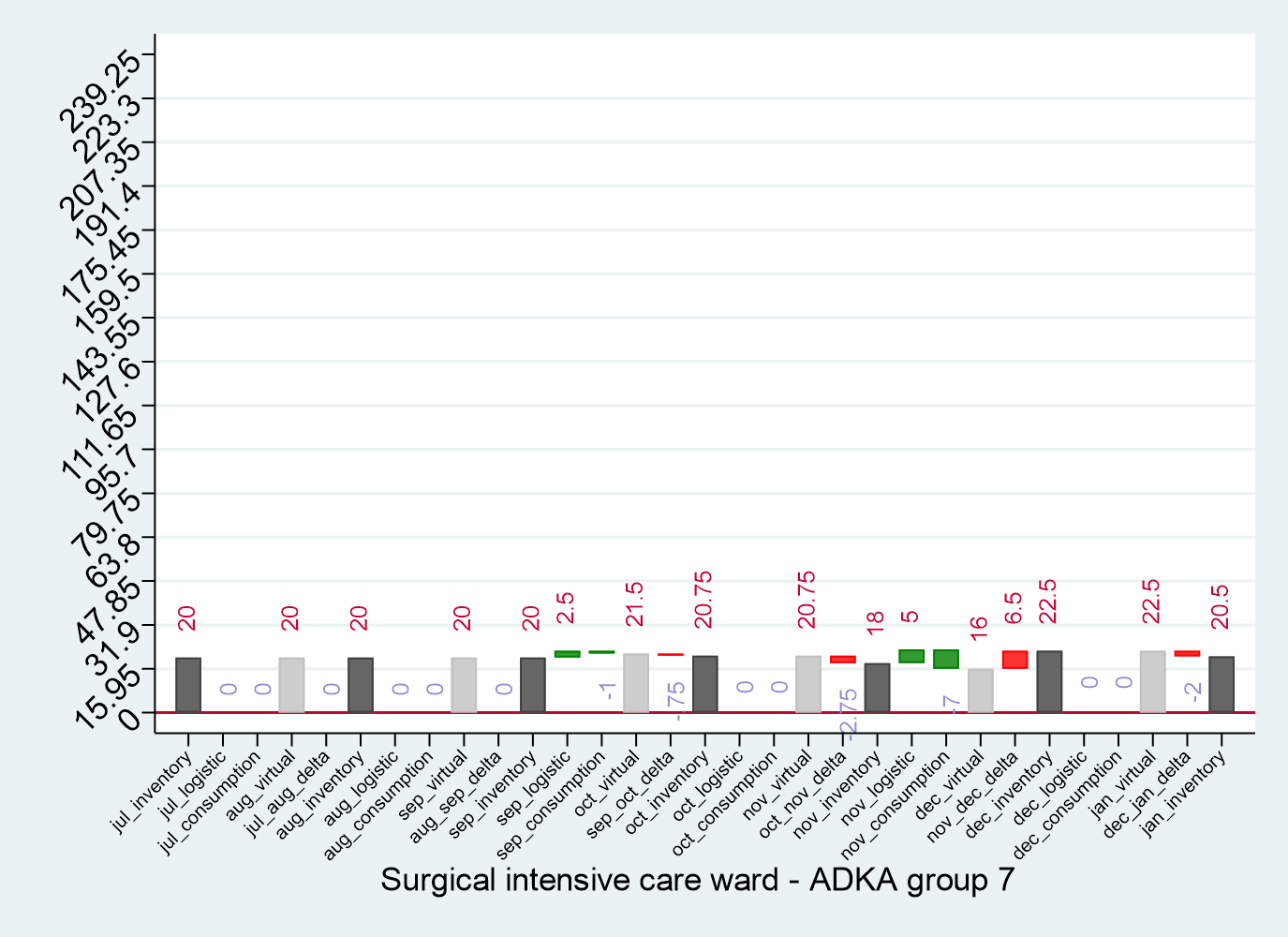

Supplement: Supplementary file 1 [file antibiotics-14-00043-s001.zip › Figure S33 - Surgical intensive care ward - ADKA group 7.png]

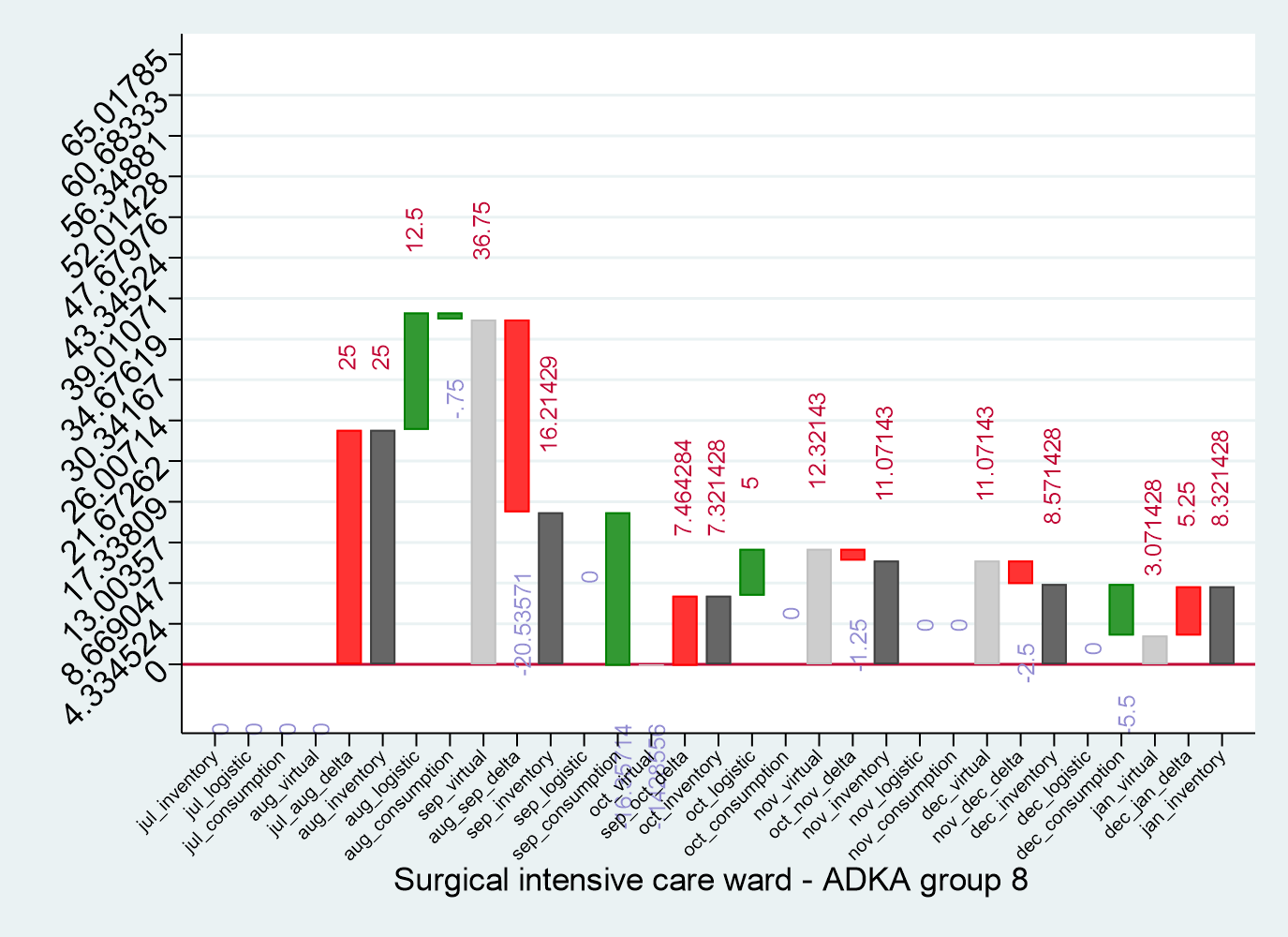

Supplement: Supplementary file 1 [file antibiotics-14-00043-s001.zip › Figure S34 - Surgical intensive care ward - ADKA group 8.png]

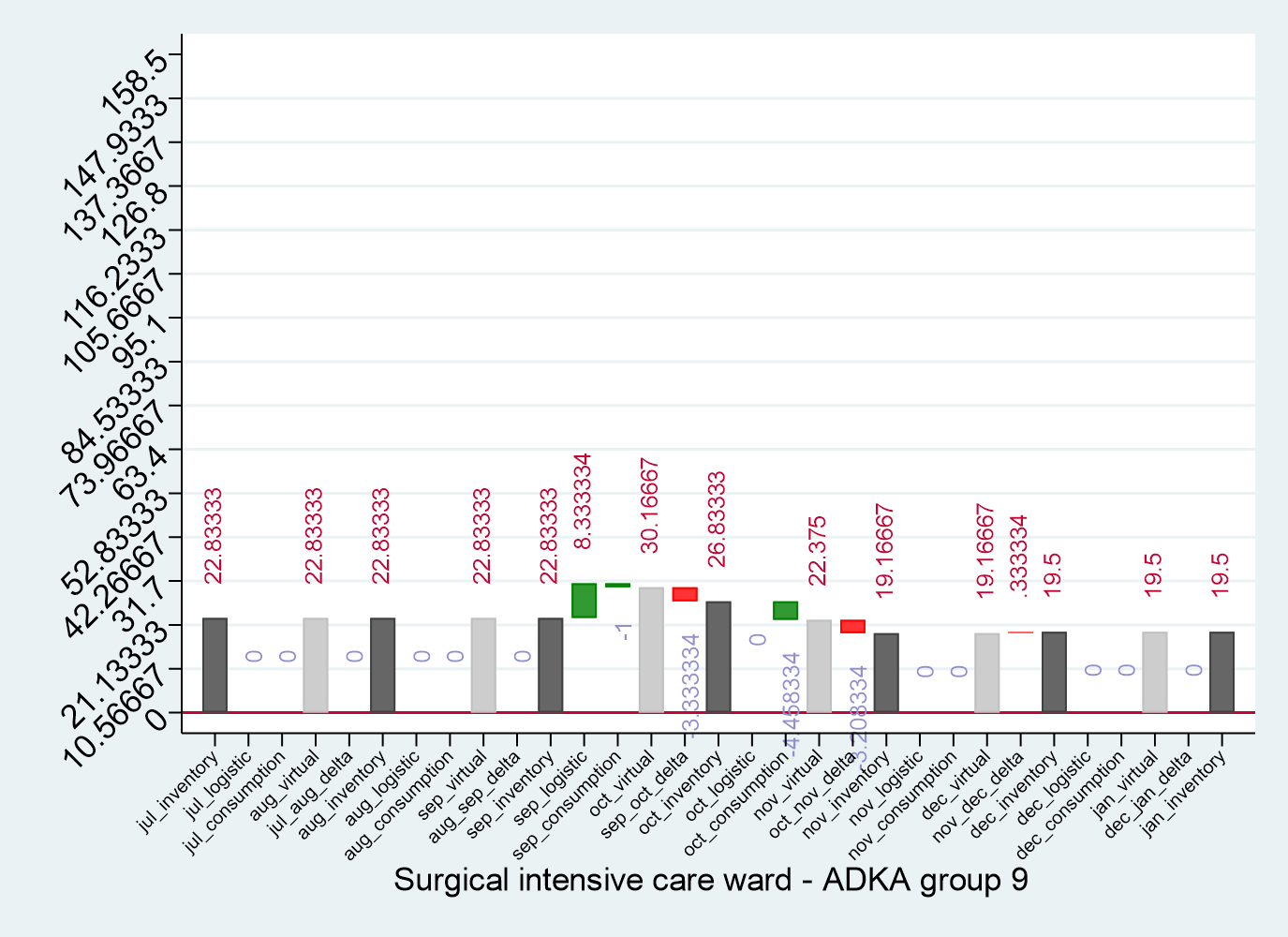

Supplement: Supplementary file 1 [file antibiotics-14-00043-s001.zip › Figure S35 - Surgical intensive care ward - ADKA group 9.png]

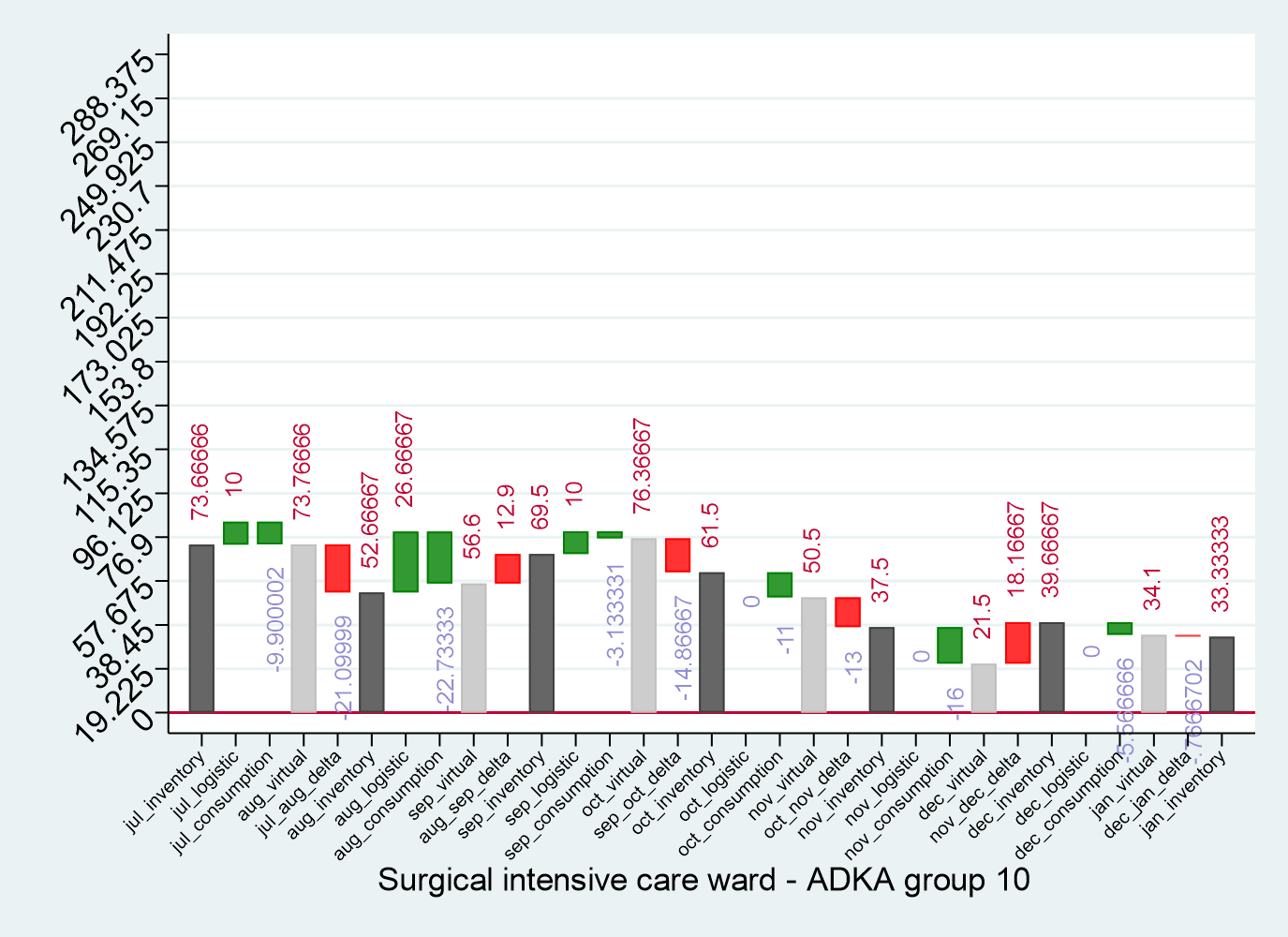

Supplement: Supplementary file 1 [file antibiotics-14-00043-s001.zip › Figure S36 - Surgical intensive care ward - ADKA group 10.png]

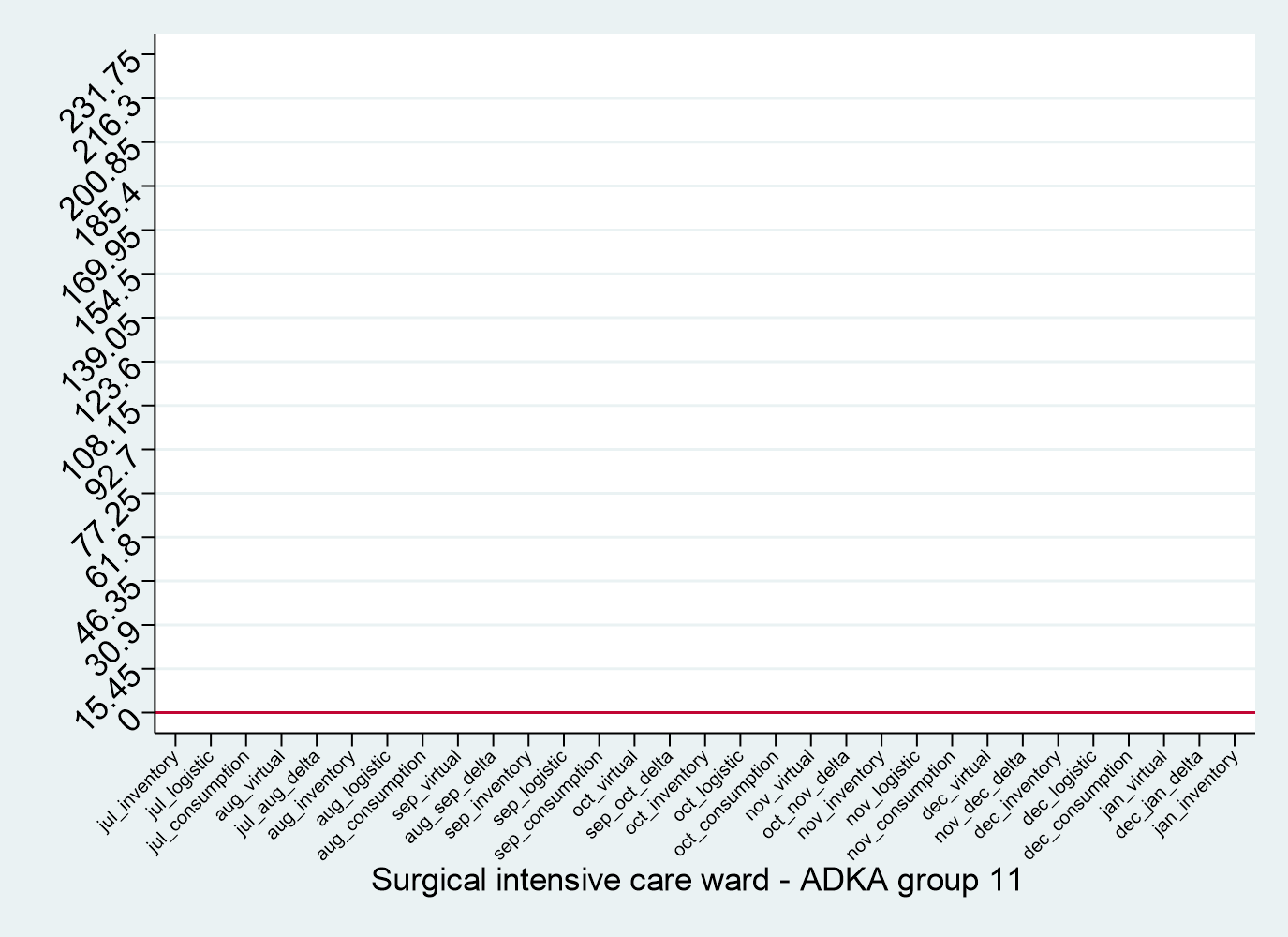

Supplement: Supplementary file 1 [file antibiotics-14-00043-s001.zip › Figure S37 - Surgical intensive care ward - ADKA group 11.png]

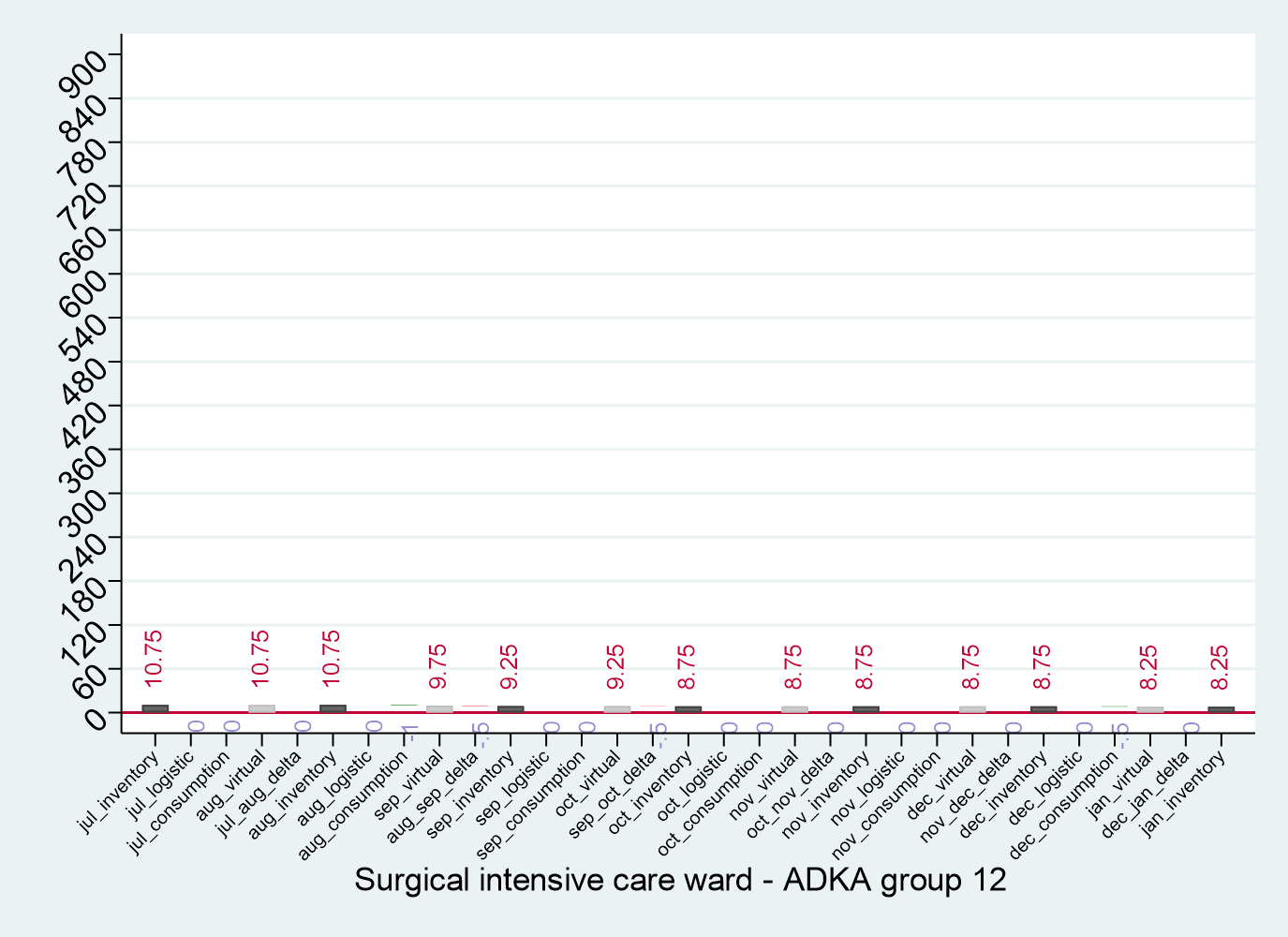

Supplement: Supplementary file 1 [file antibiotics-14-00043-s001.zip › Figure S38 - Surgical intensive care ward - ADKA group 12.png]

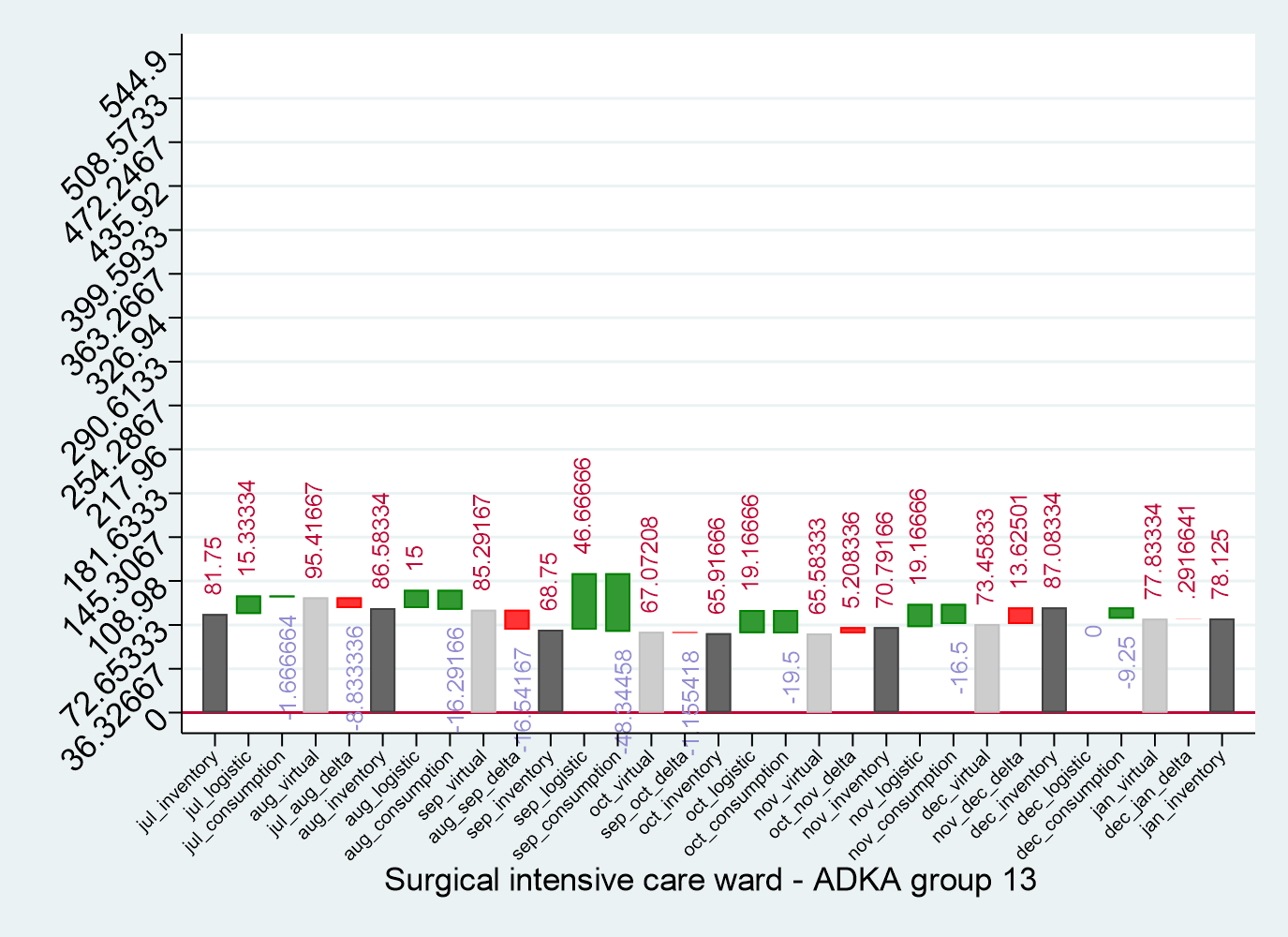

Supplement: Supplementary file 1 [file antibiotics-14-00043-s001.zip › Figure S39 - Surgical intensive care ward - ADKA group 13.png]

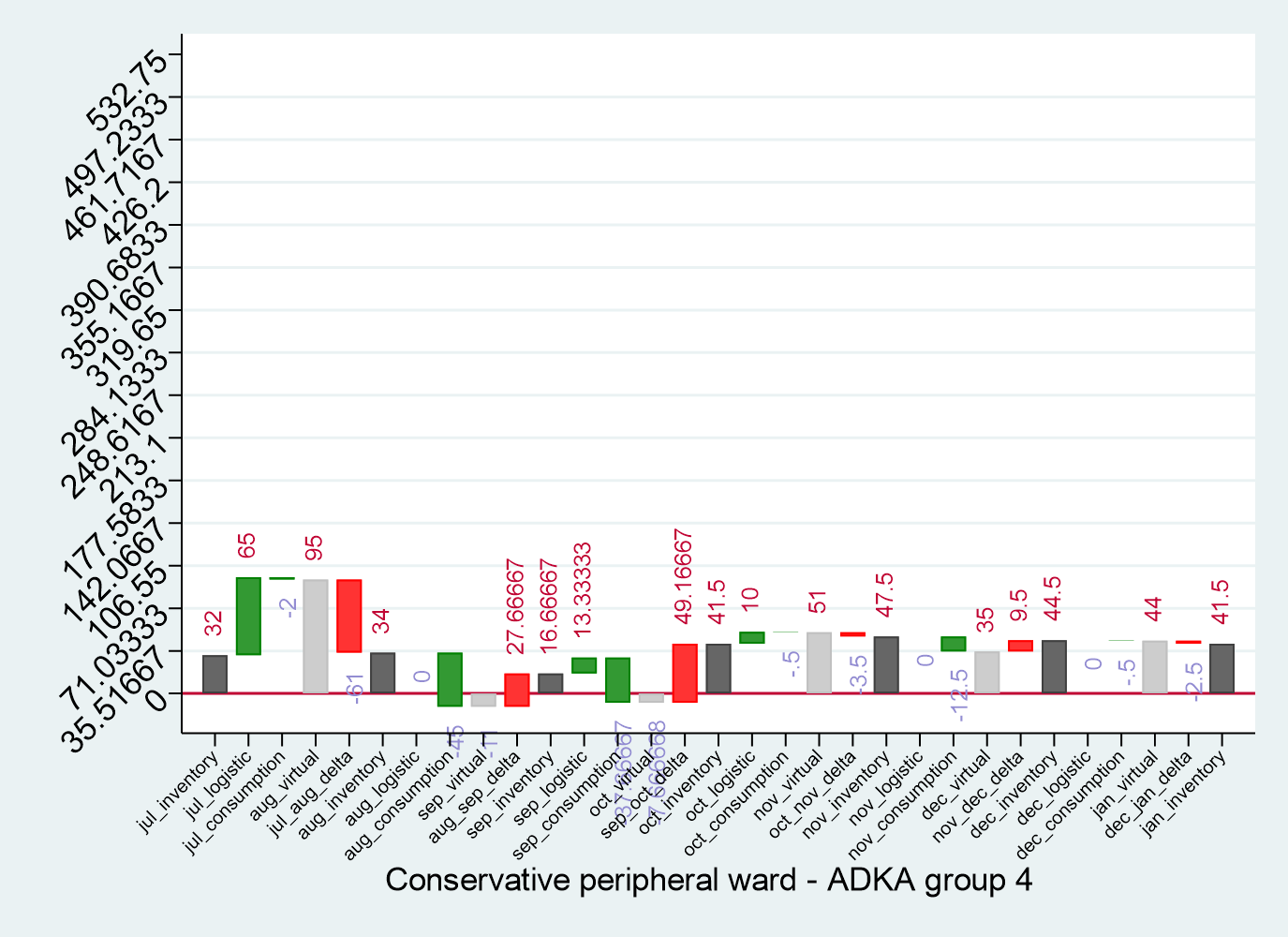

Supplement: Supplementary file 1 [file antibiotics-14-00043-s001.zip › Figure S4 - Conservative peripheral ward - ADKA group 4.png]

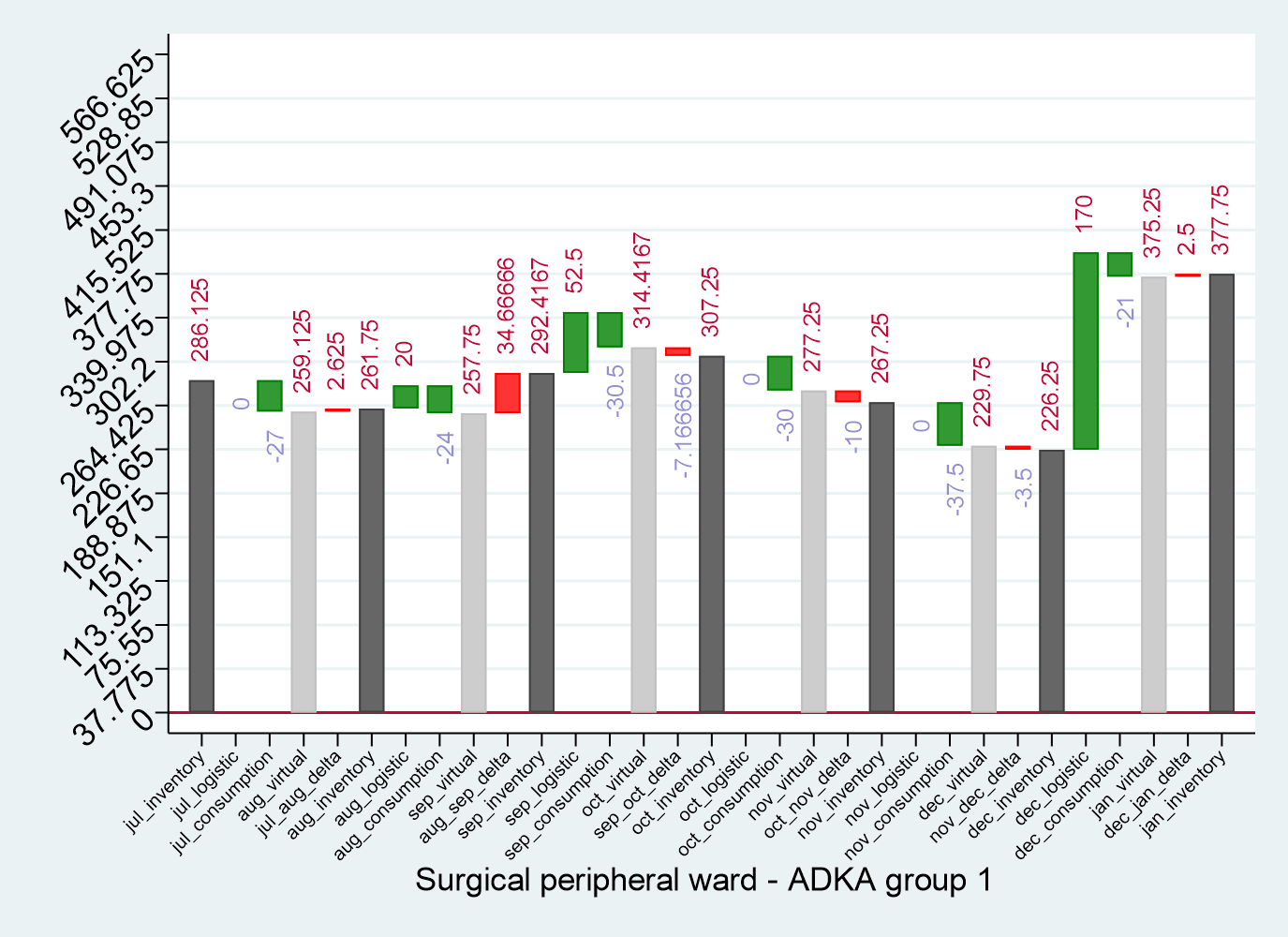

Supplement: Supplementary file 1 [file antibiotics-14-00043-s001.zip › Figure S40 - Surgical peripheral ward - ADKA group 1.png]

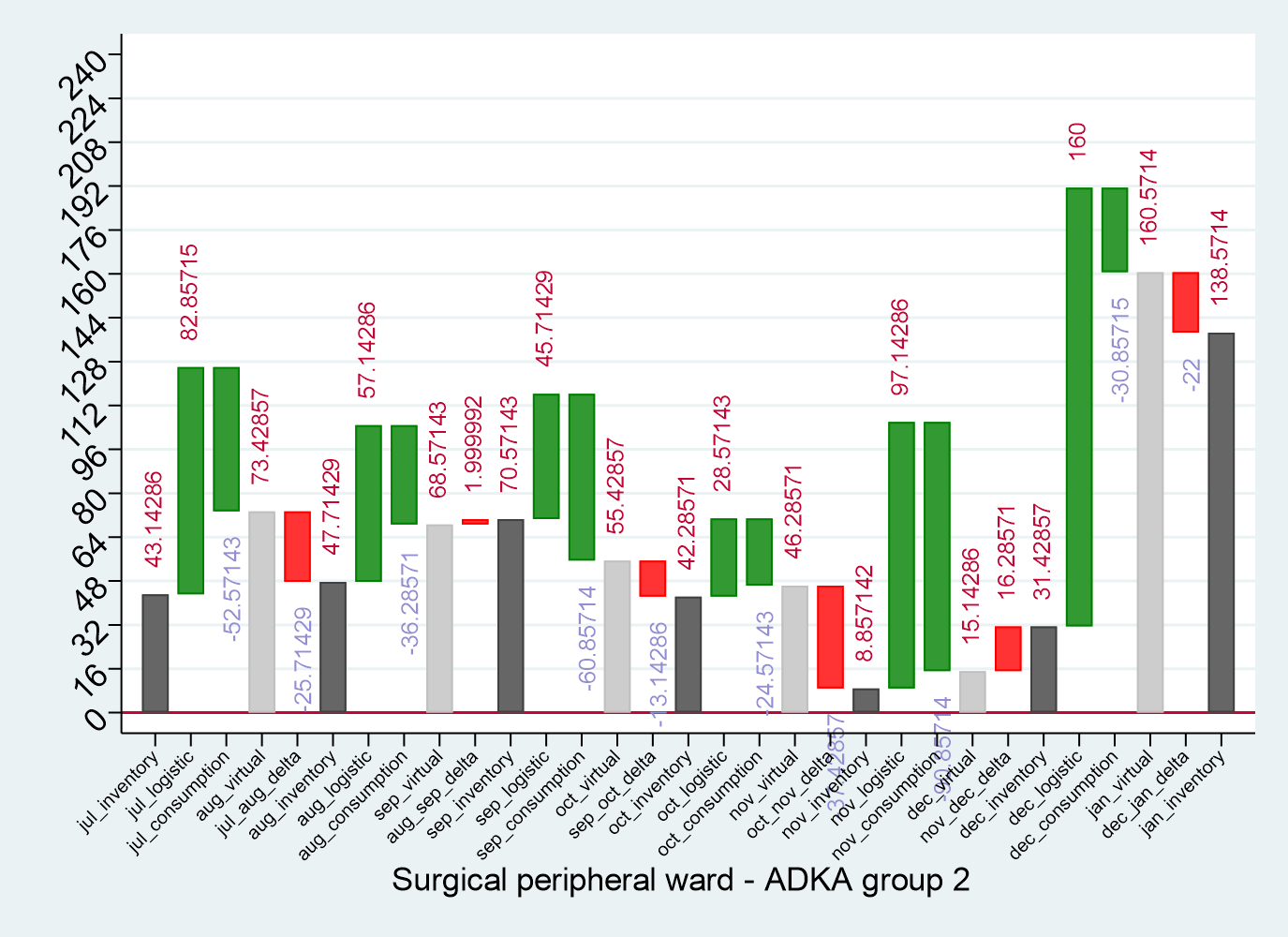

Supplement: Supplementary file 1 [file antibiotics-14-00043-s001.zip › Figure S41 - Surgical peripheral ward - ADKA group 2.png]

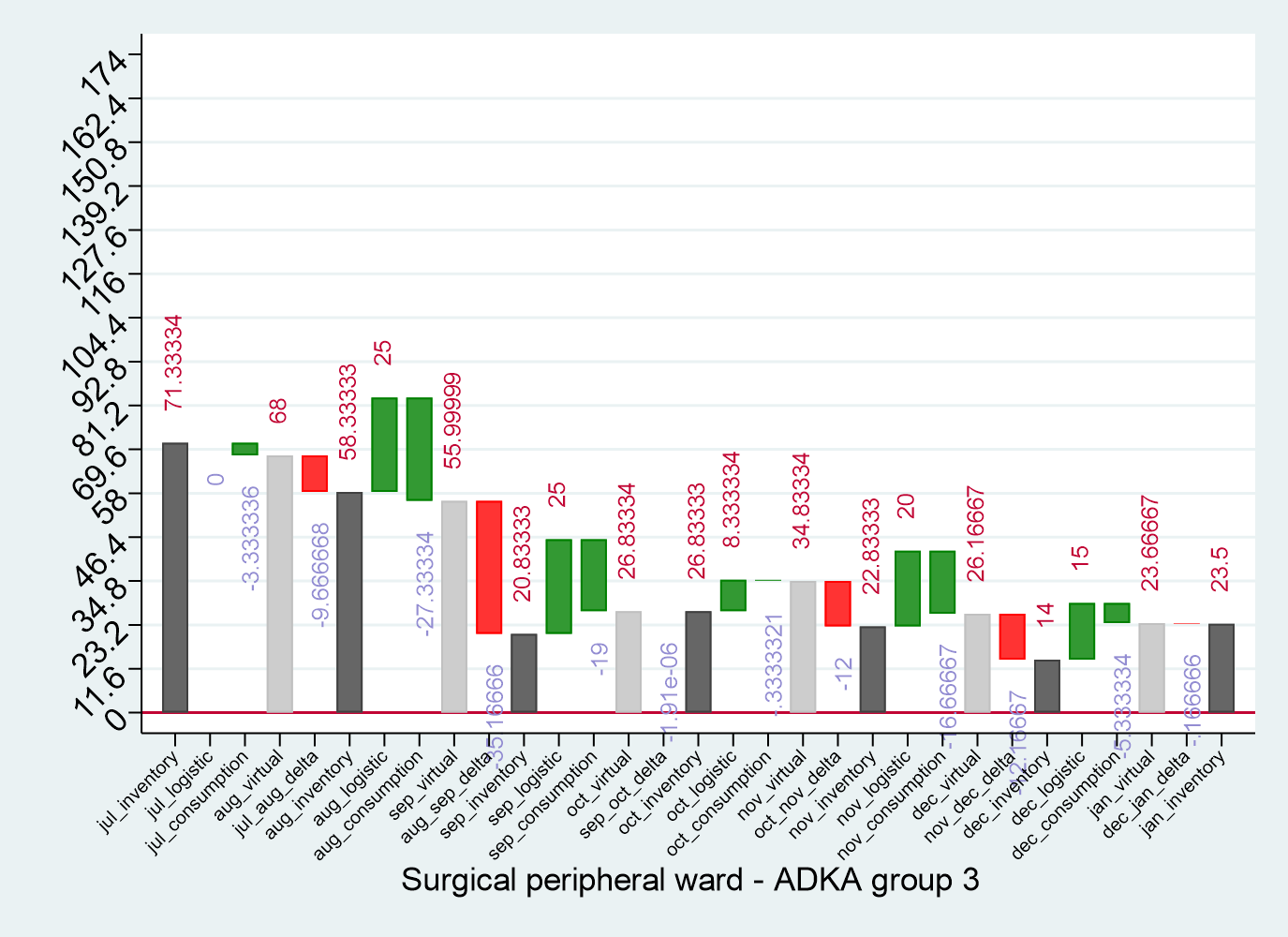

Supplement: Supplementary file 1 [file antibiotics-14-00043-s001.zip › Figure S42 - Surgical peripheral ward - ADKA group 3.png]

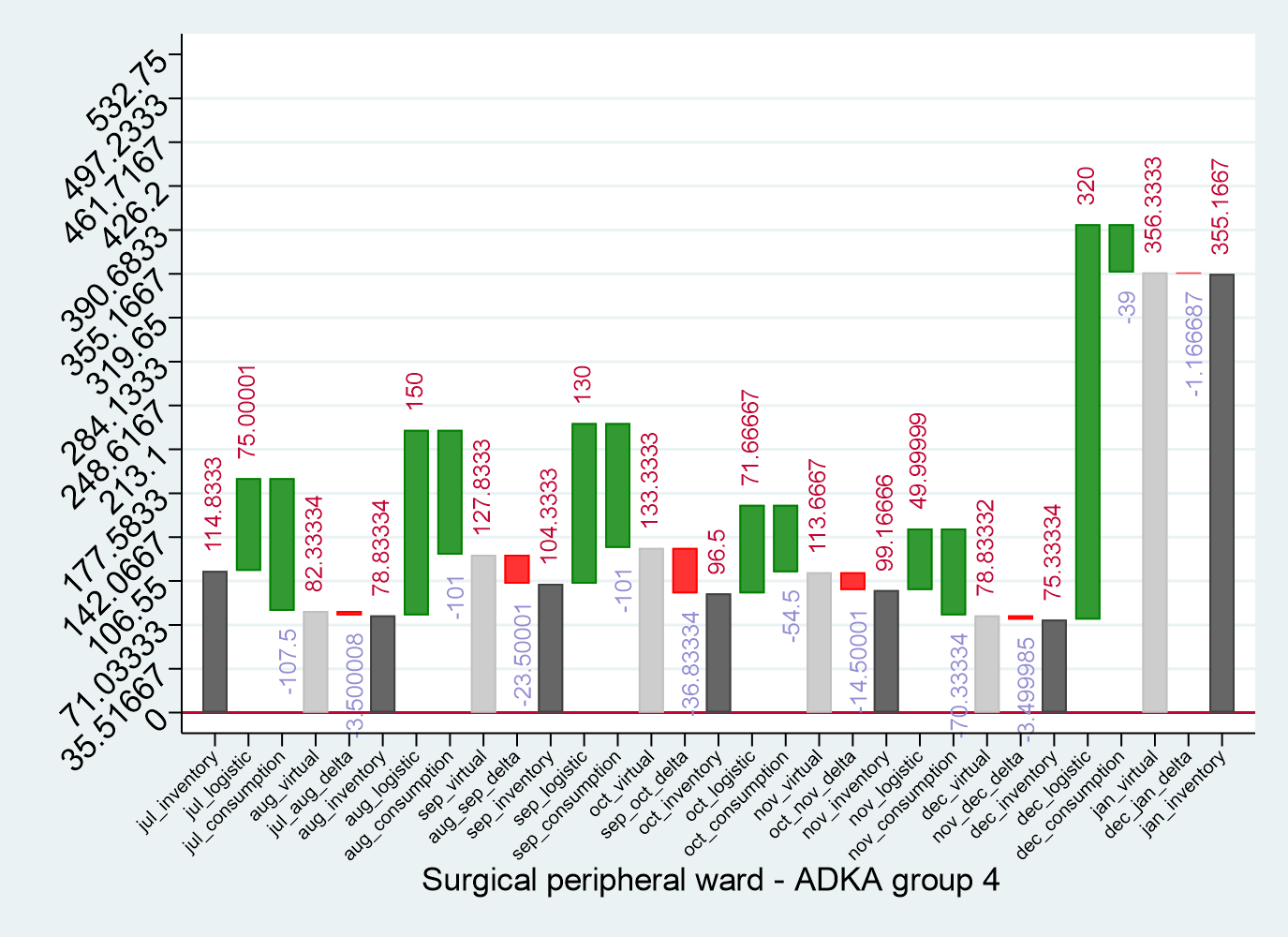

Supplement: Supplementary file 1 [file antibiotics-14-00043-s001.zip › Figure S43 - Surgical peripheral ward - ADKA group 4.png]

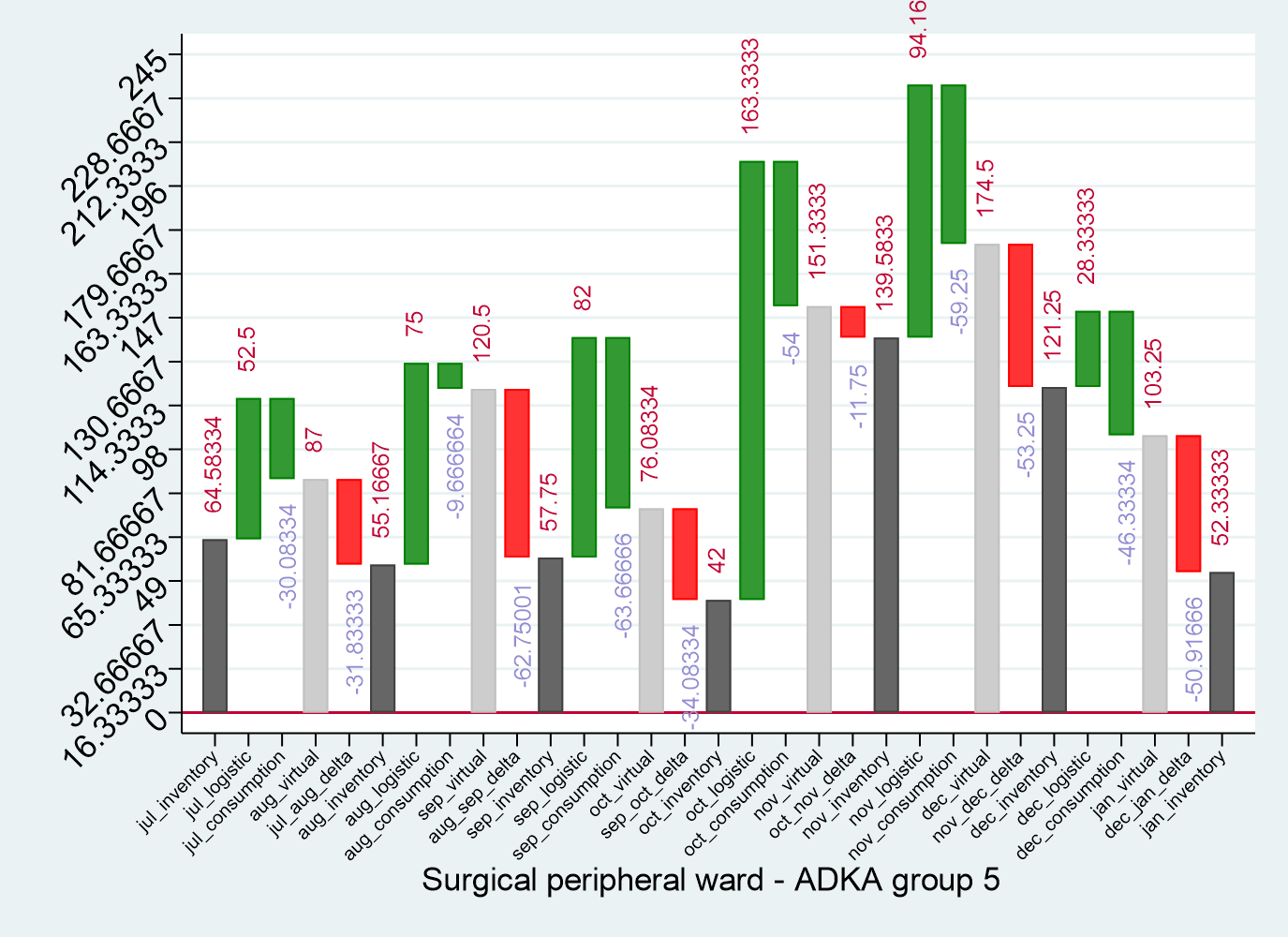

Supplement: Supplementary file 1 [file antibiotics-14-00043-s001.zip › Figure S44 - Surgical peripheral ward - ADKA group 5.png]

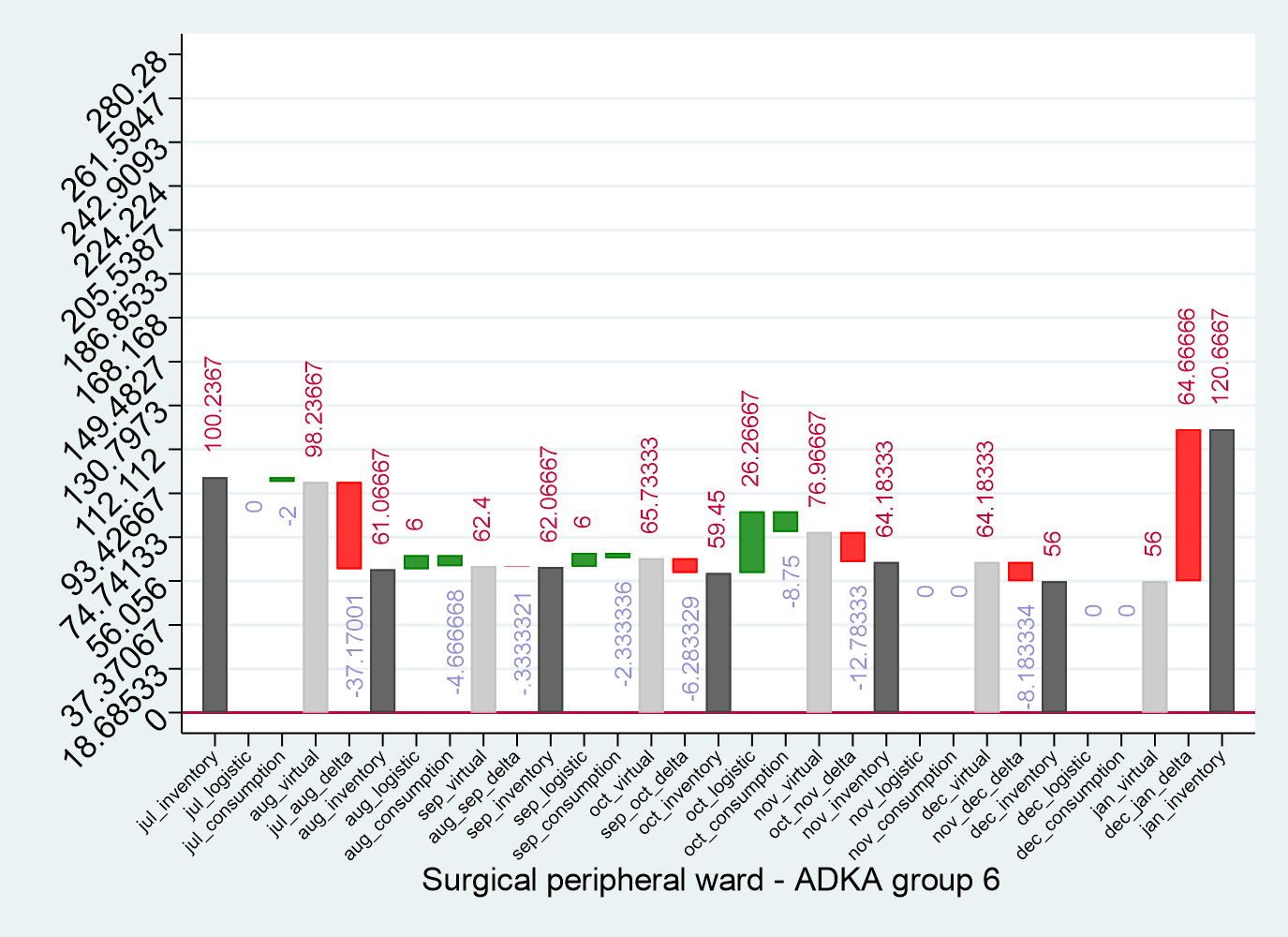

Supplement: Supplementary file 1 [file antibiotics-14-00043-s001.zip › Figure S45 - Surgical peripheral ward - ADKA group 6.png]

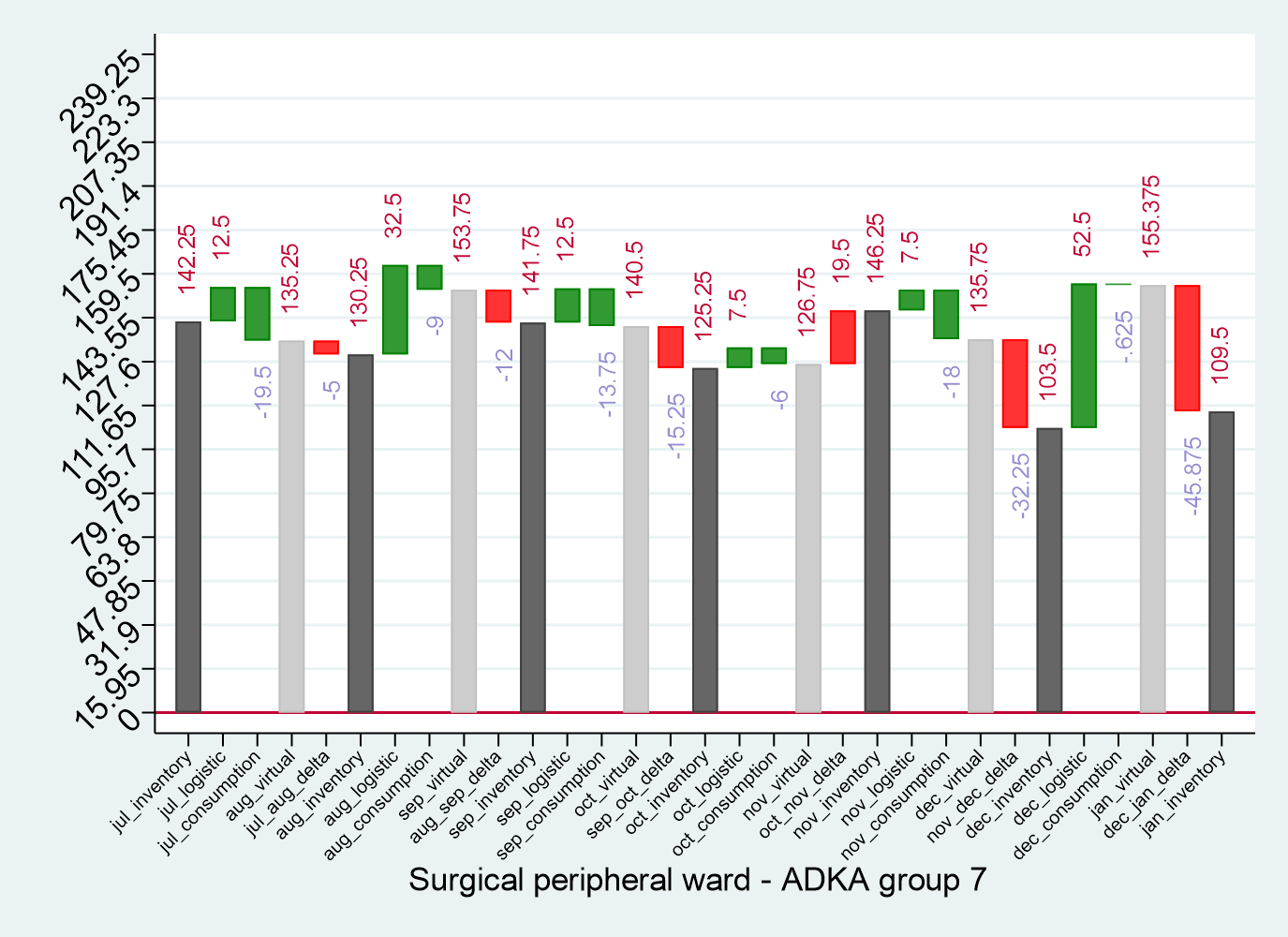

Supplement: Supplementary file 1 [file antibiotics-14-00043-s001.zip › Figure S46 - Surgical peripheral ward - ADKA group 7.png]

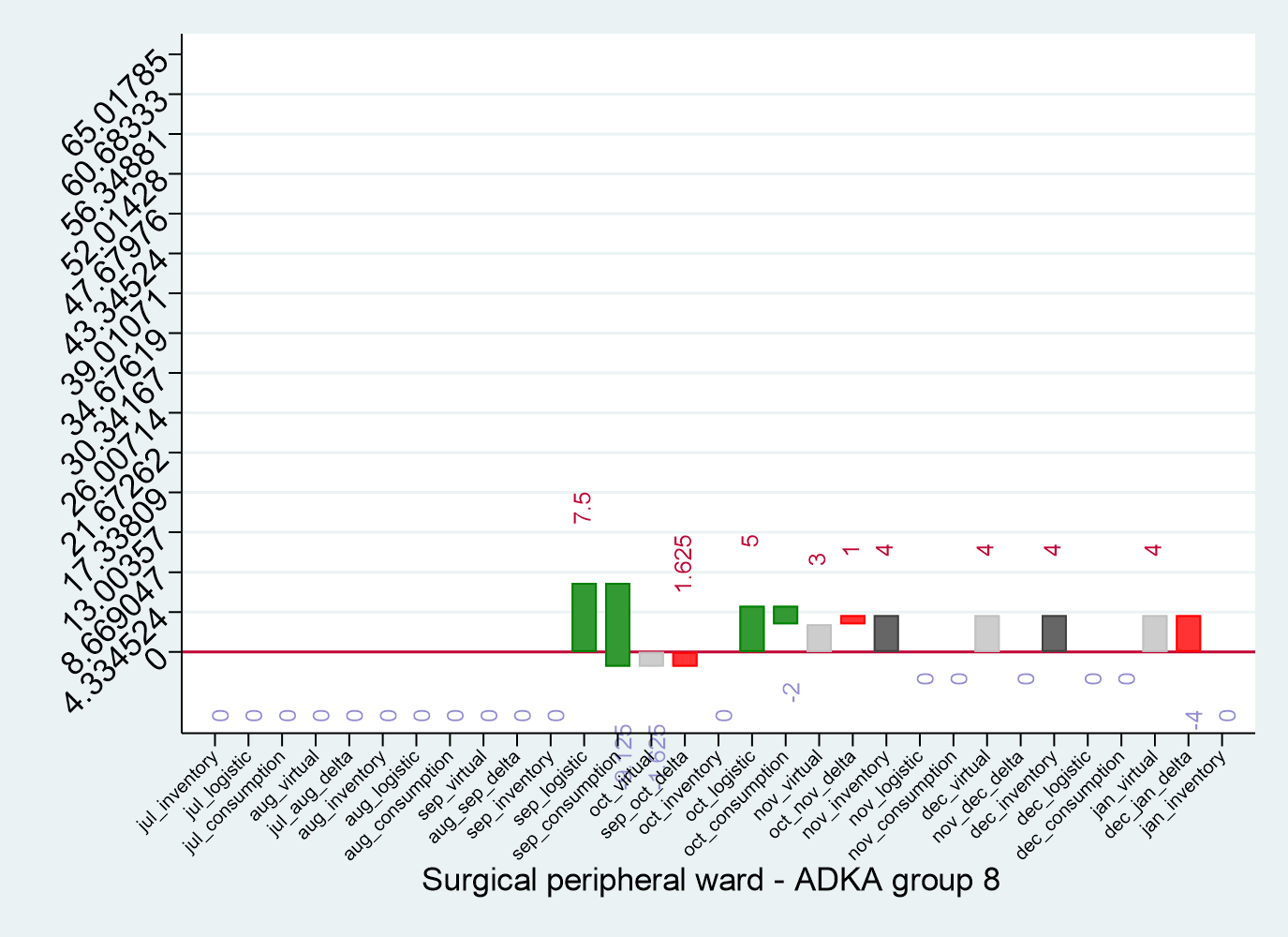

Supplement: Supplementary file 1 [file antibiotics-14-00043-s001.zip › Figure S47 - Surgical peripheral ward - ADKA group 8.png]

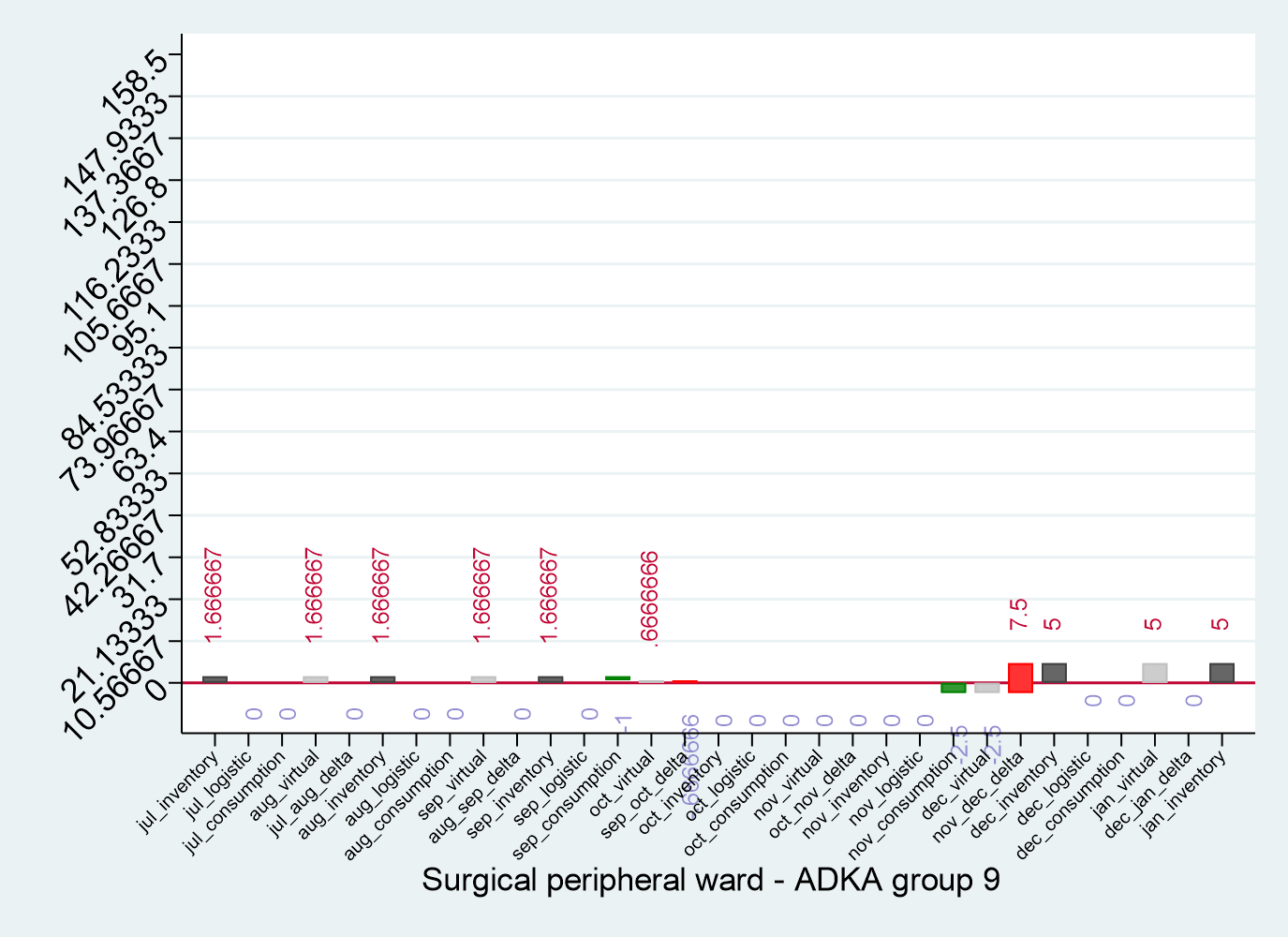

Supplement: Supplementary file 1 [file antibiotics-14-00043-s001.zip › Figure S48 - Surgical peripheral ward - ADKA group 9.png]

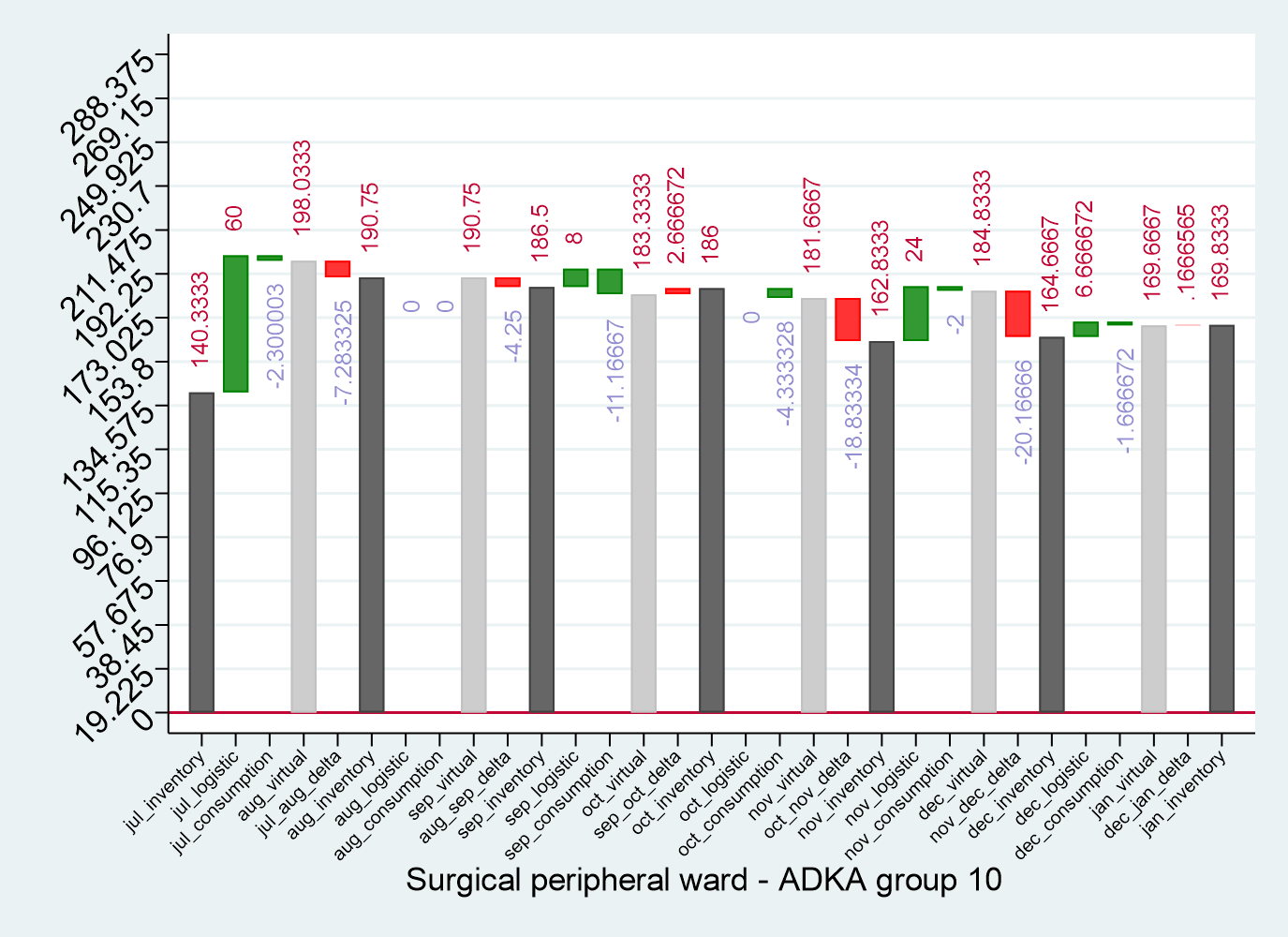

Supplement: Supplementary file 1 [file antibiotics-14-00043-s001.zip › Figure S49 - Surgical peripheral ward - ADKA group 10.png]

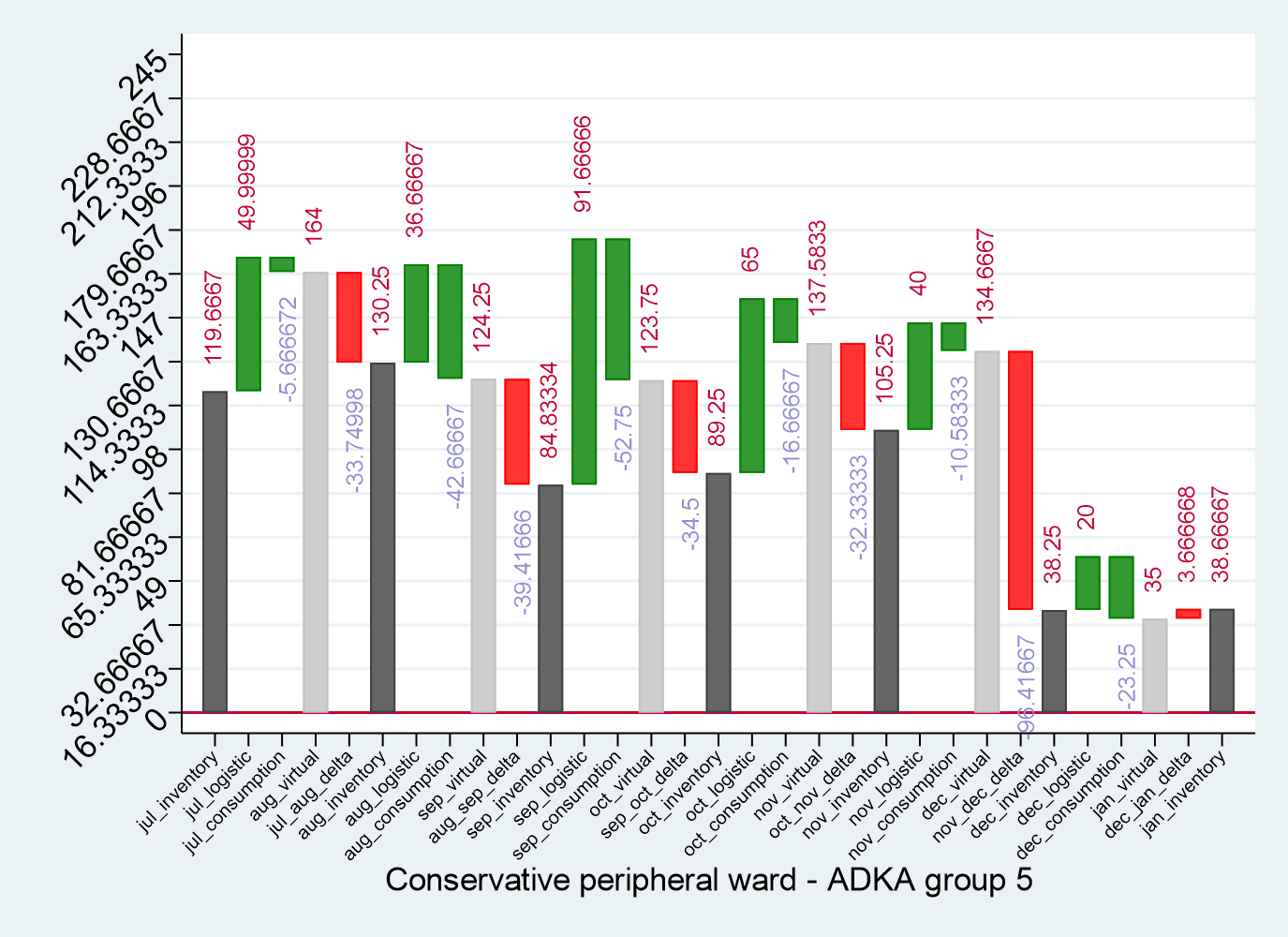

Supplement: Supplementary file 1 [file antibiotics-14-00043-s001.zip › Figure S5 - Conservative peripheral ward - ADKA group 5.png]

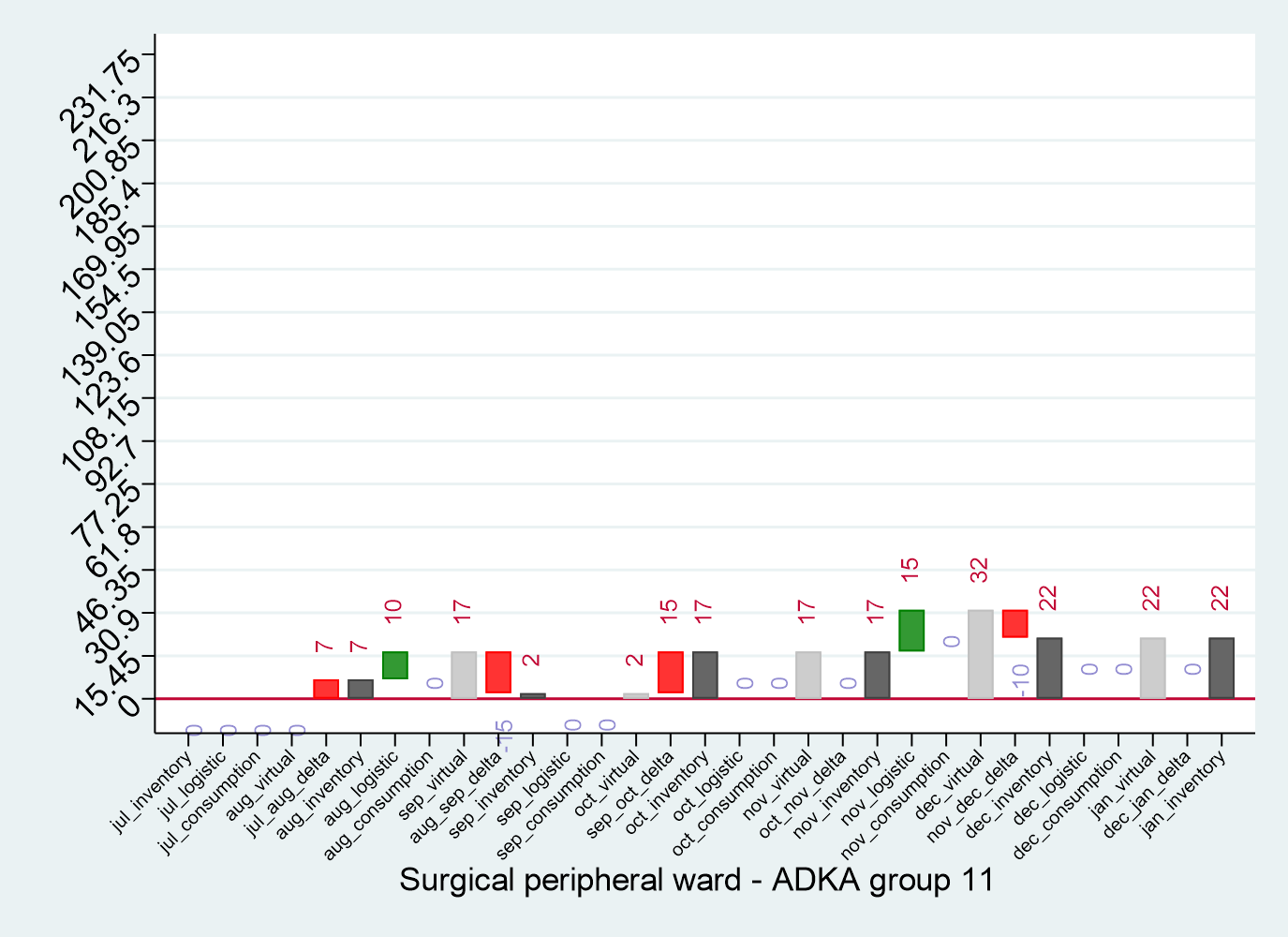

Supplement: Supplementary file 1 [file antibiotics-14-00043-s001.zip › Figure S50 - Surgical peripheral ward - ADKA group 11.png]

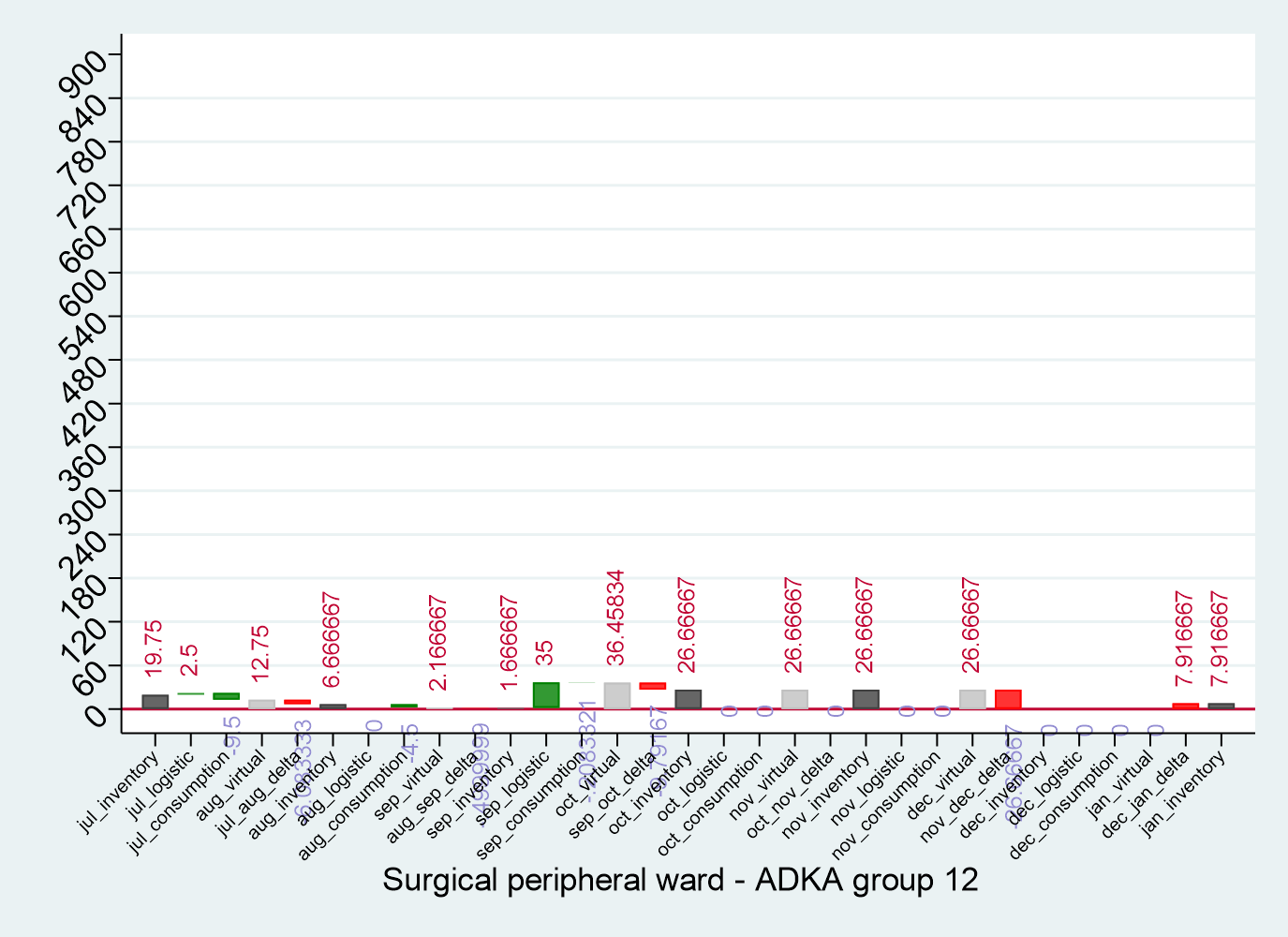

Supplement: Supplementary file 1 [file antibiotics-14-00043-s001.zip › Figure S51 - Surgical peripheral ward - ADKA group 12.png]

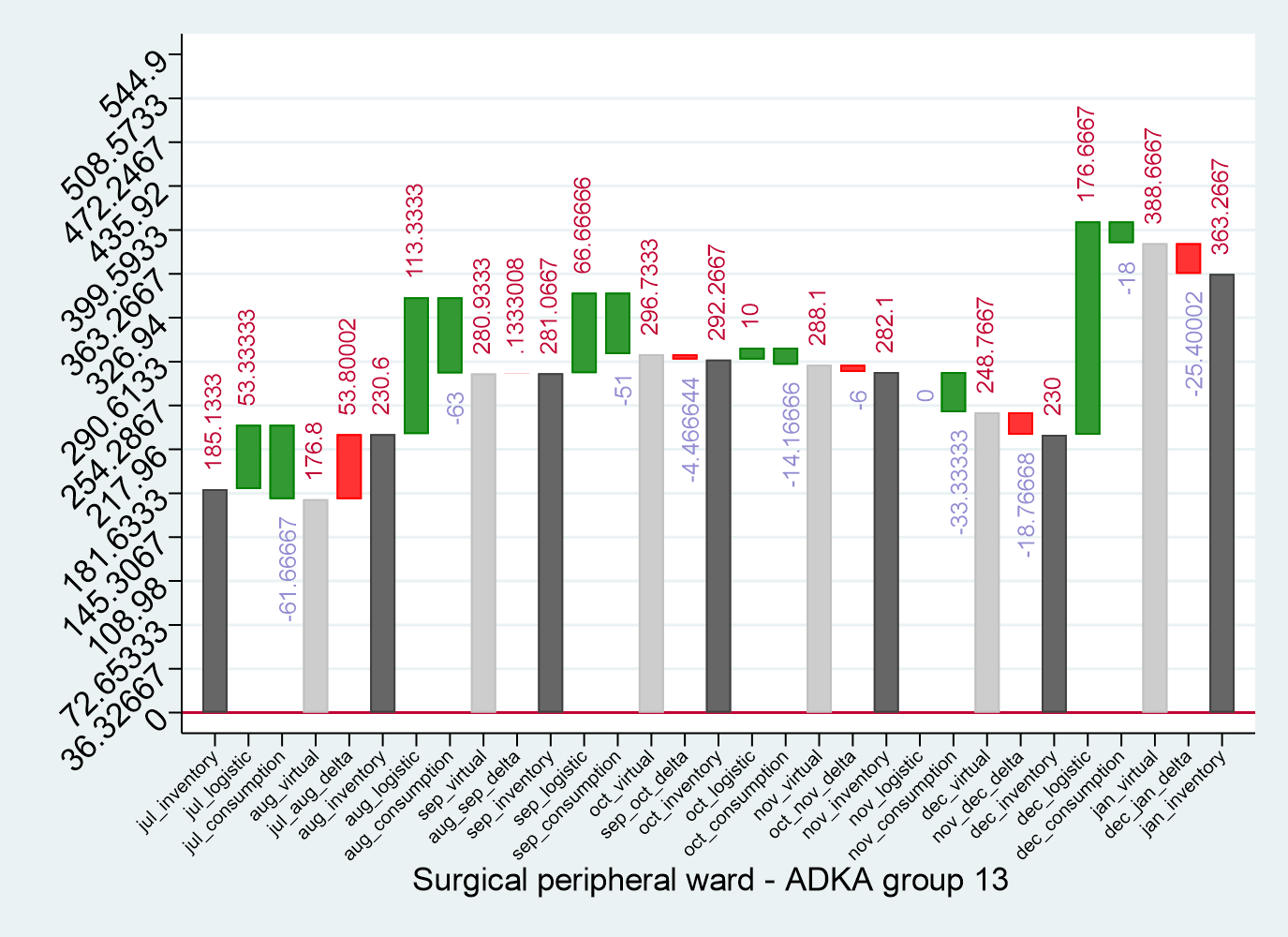

Supplement: Supplementary file 1 [file antibiotics-14-00043-s001.zip › Figure S52 - Surgical peripheral ward - ADKA group 13.png]

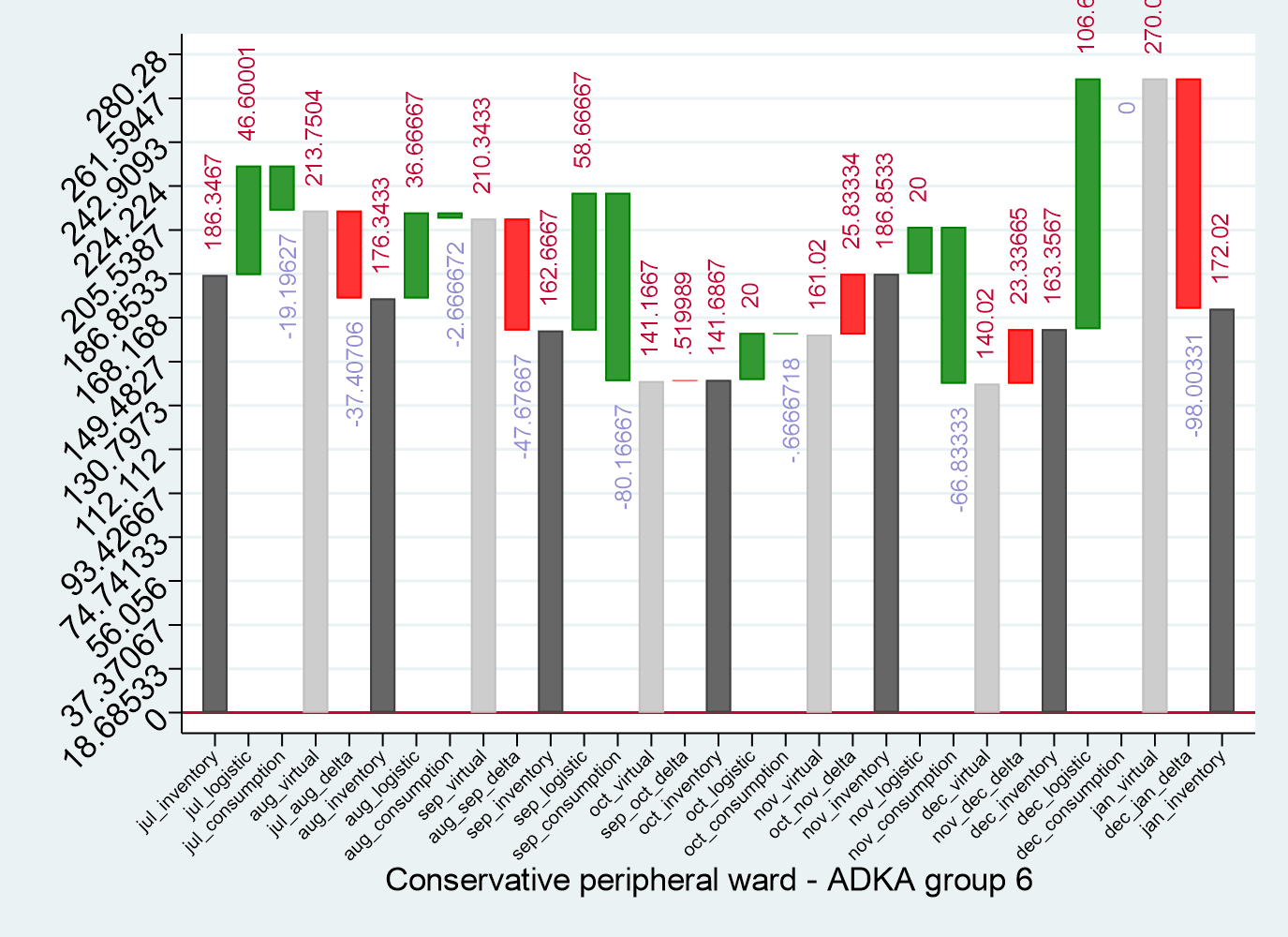

Supplement: Supplementary file 1 [file antibiotics-14-00043-s001.zip › Figure S6 - Conservative peripheral ward - ADKA group 6.png]

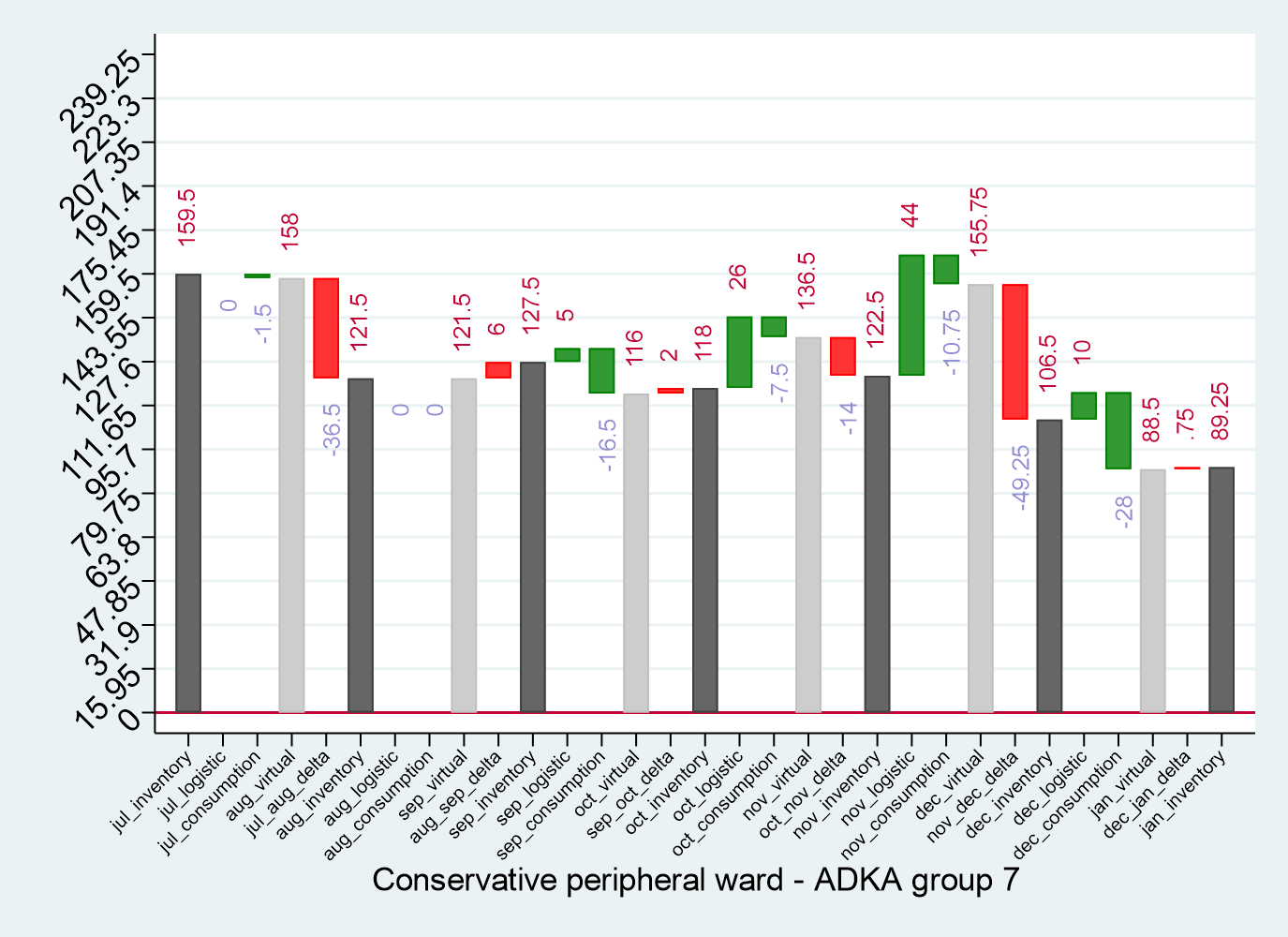

Supplement: Supplementary file 1 [file antibiotics-14-00043-s001.zip › Figure S7 - Conservative peripheral ward - ADKA group 7.png]

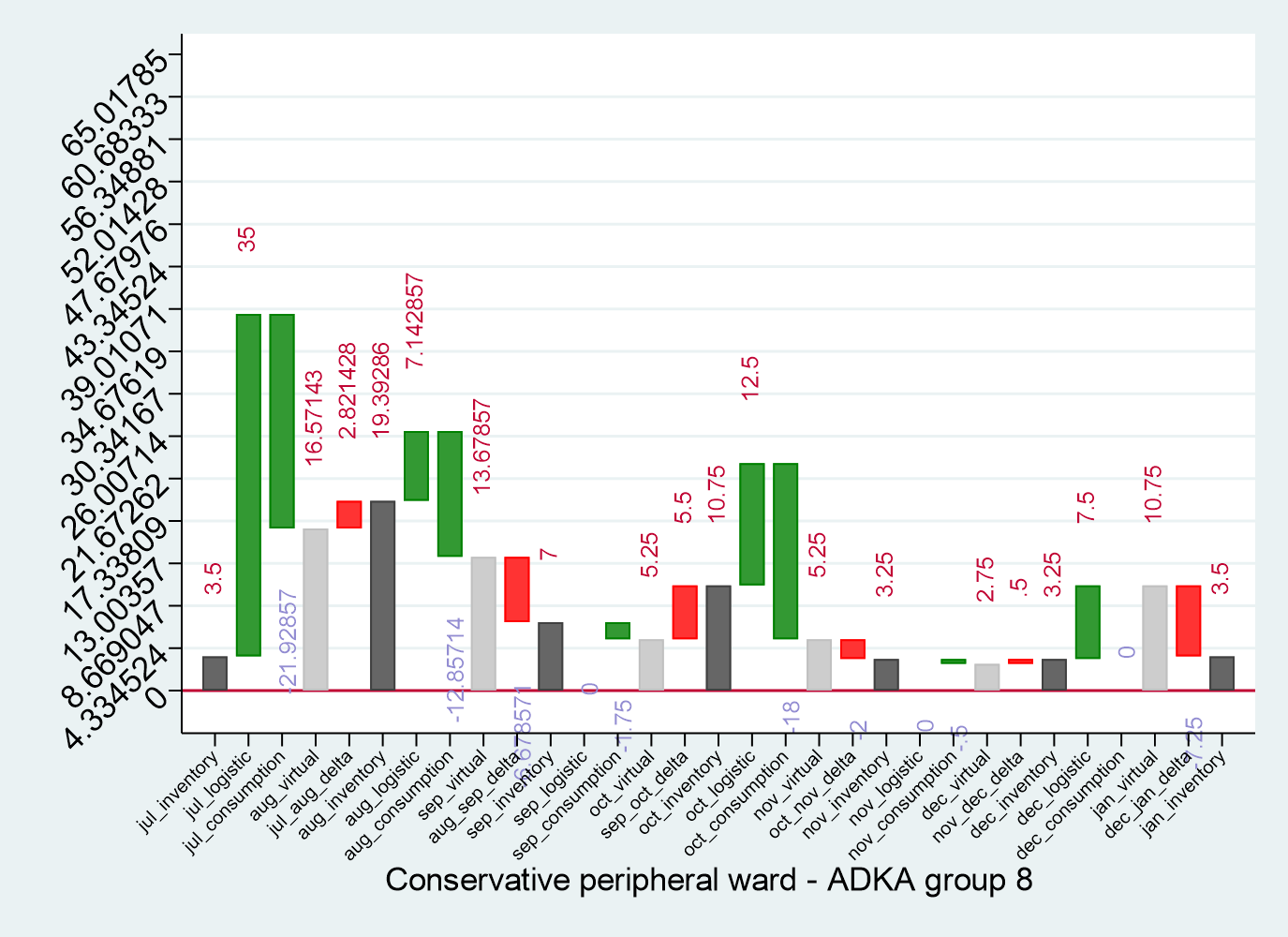

Supplement: Supplementary file 1 [file antibiotics-14-00043-s001.zip › Figure S8 - Conservative peripheral ward - ADKA group 8.png]

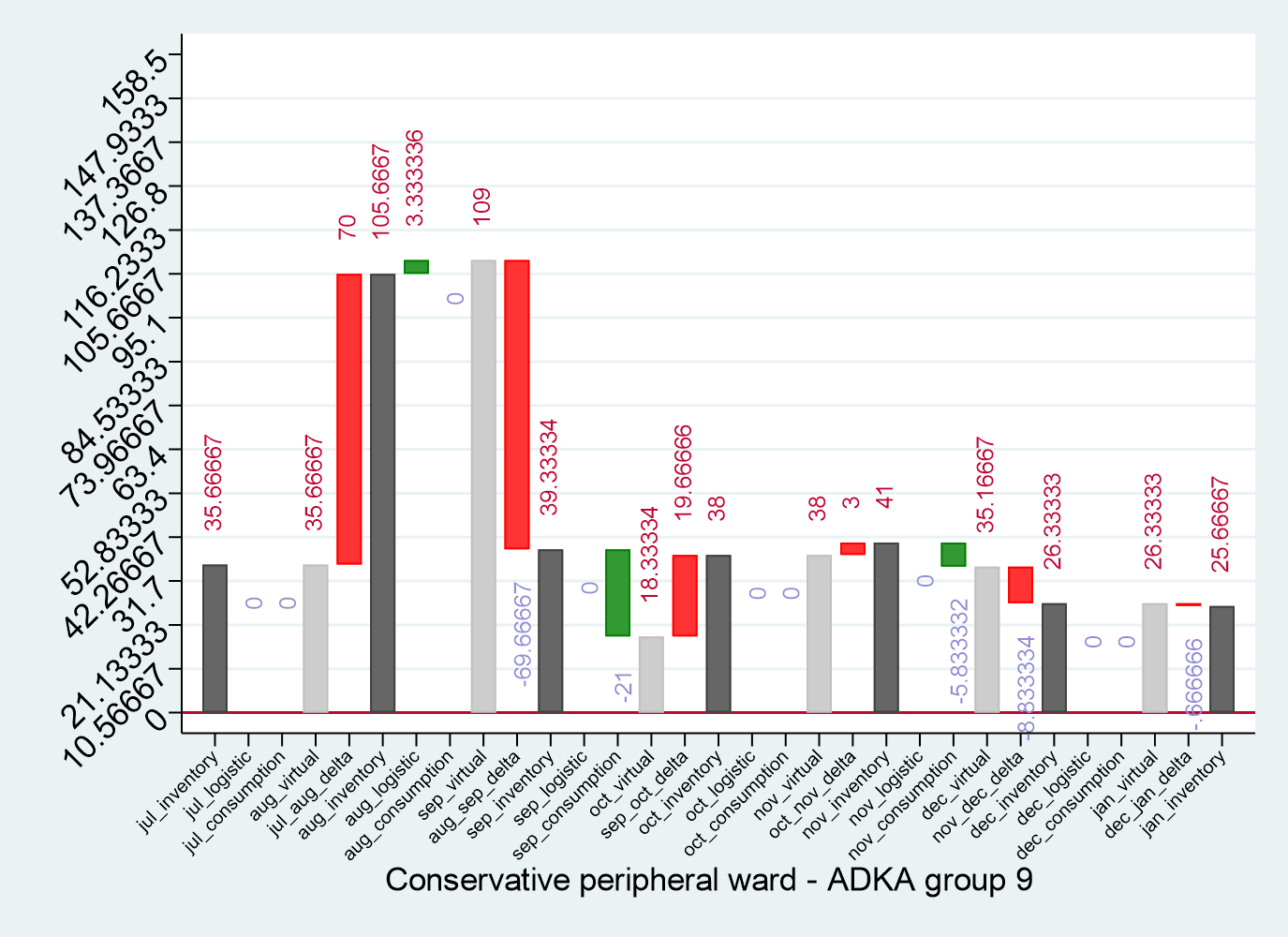

Supplement: Supplementary file 1 [file antibiotics-14-00043-s001.zip › Figure S9 - Conservative peripheral ward - ADKA group 9.png]
